# Supplementary material for: RAS-independent ERK activation by constitutively active KSR3 in non-chordate metazoa
Source: Nat Commun. 2023 Jul 5;14:3970. doi: 10.1038/s41467-023-39606-y (PMC10322840; doi:10.1038/s41467-023-39606-y)
Supplement: Supplementary file 1 — Supplementary Information [file 41467_2023_39606_MOESM1_ESM.pdf]

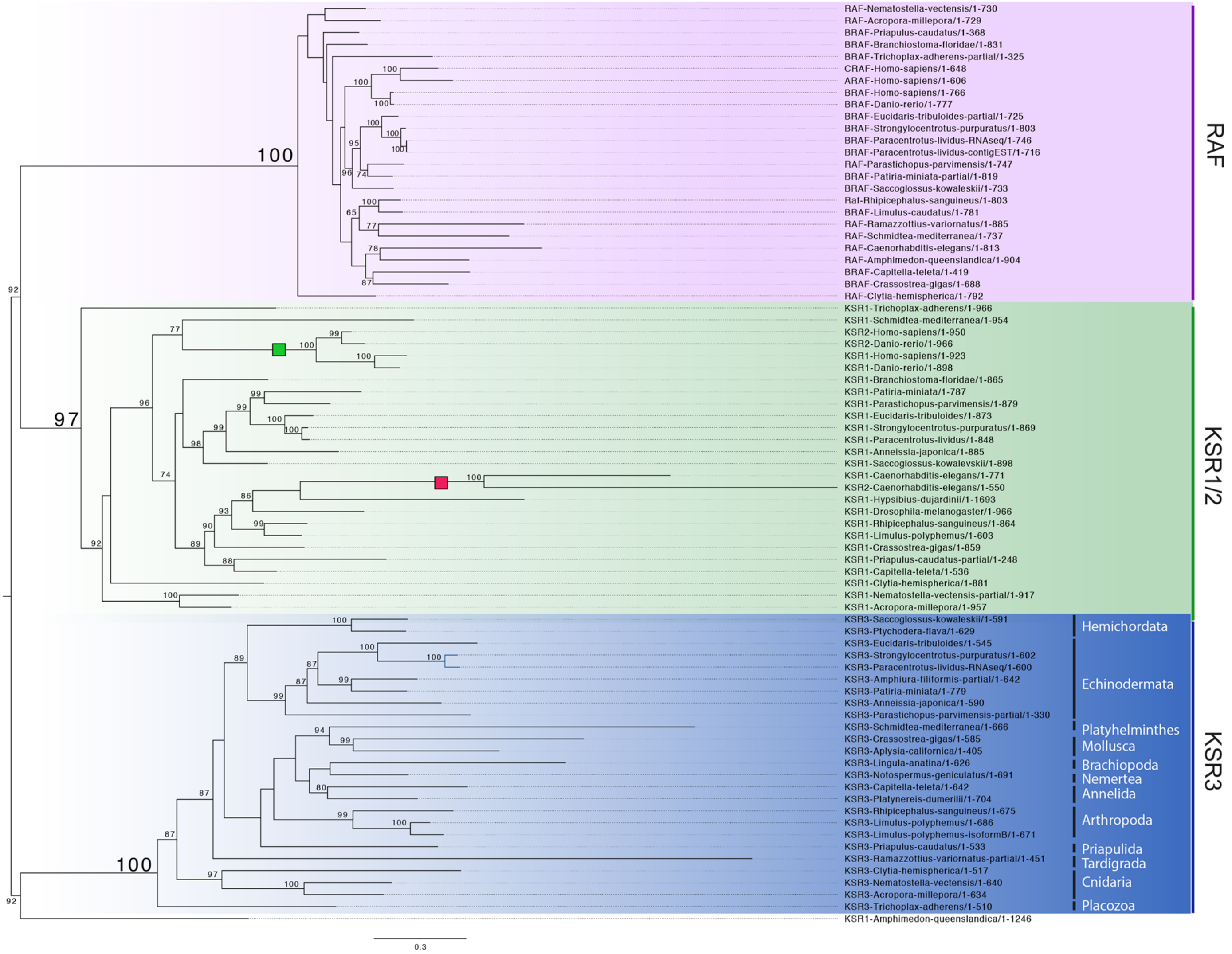


**Supplementary Figure 1.**

***ksr3* belongs to an ancient gene family present in the genome of animals representative of most non-chordate phyla.** Phylogenetic analysis using maximum likelihood of KSR and RAF sequences. RAF and KSR sequences from various phyla were retrieved from Genebank and aligned using ClustalW. Trees were built using the maximum likelihood method. A consensus tree with 70% cut off value was derived from 1000 replicates of ultrafast Bootstrap approximation. Numbers above branches represent bootstrap values, calculated from this consensus. The topology of the tree reveals that *ksr3* genes are ancient genes, present in the genomes of various phyla comprising both diploblastic and triploblastic animal and covering both protostomia and deuterostomia. Note however, that *ksr3* has been lost in chordates as well as in nematodes and insects, and that a recent duplication of *ksr1* gene occurred in nematodes and vertebrates (represented by a red and a green square, respectively).

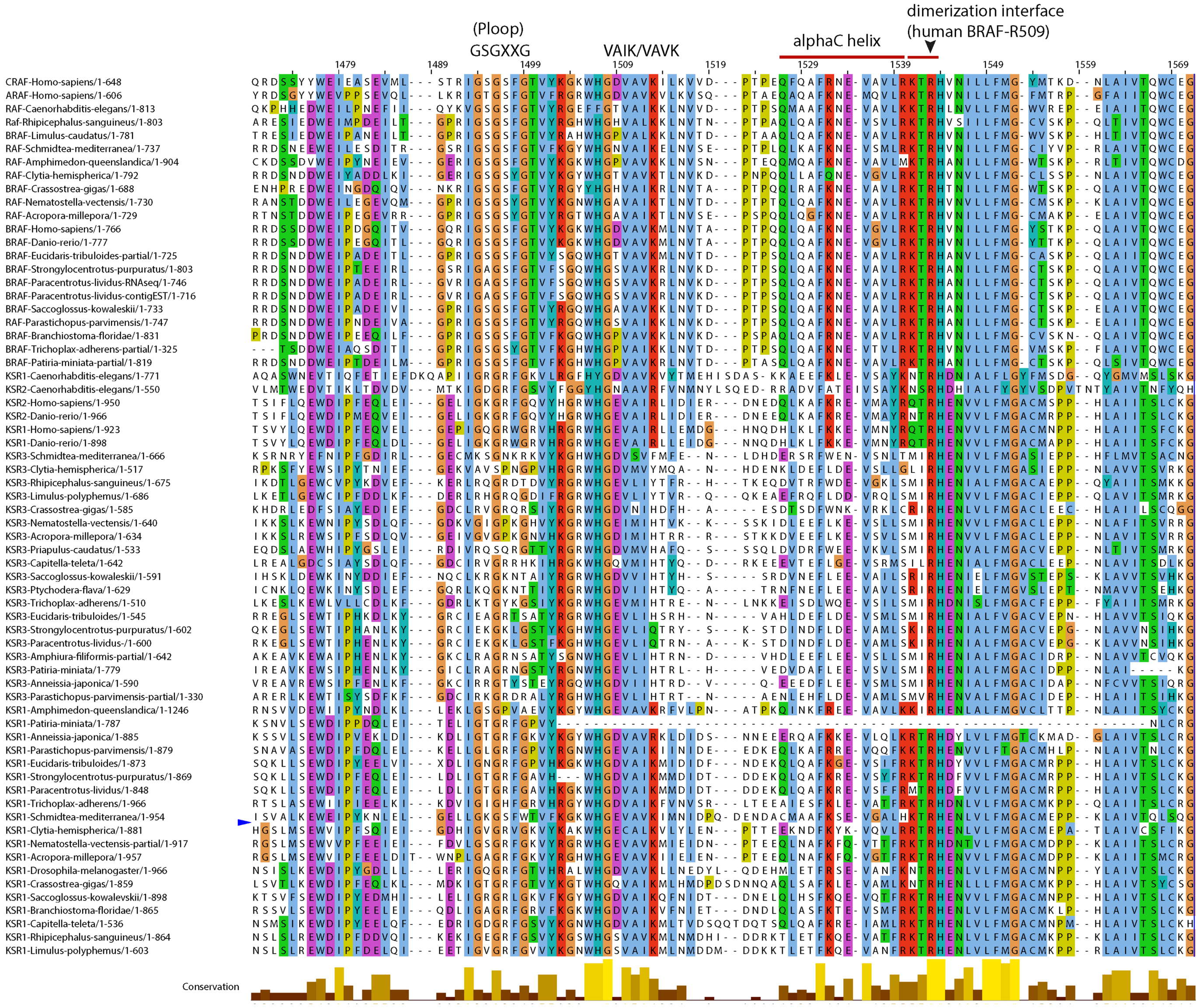


**Supplementary Figure 2**

**Sequence alignment of the N-terminal lobe of the kinase domain of 21 RAF, 19 KSR3 and 22 KSR1/2 cDNA sequences factors from different phyla reveals the existence of an ancient family of KSR proteins with a highly remodelled ATP-binding site, alpha helix and dimerization interface**. Amino acids are coloured according to their biochemical properties to visualize conservation and similarities. The position of the GXGXXG and of the VAV/IK motifs, which are highly conserved and are part of the ATP-binding site, is indicated at the top of the sequences. Note the absence of the GXGXXG motif involved in binding of the ATP molecule in most KSR3 family members. Within cnidarians, Nematostella and Acropora RAF appear to have conserved the GXGXXG motif but they lack the conserved VAV/IK which is replaced by IMIH. RAF from the medusa *Clytia hemispherica* also lacks the GXGXXG. The position of the alpha helix is indicated by a horizontal line. Compared to the sequence of the alpha helix of RAF and KSR1family members, which contains several basic residues, the alpha helix of KSR3 is relatively acidic. The position of the arginine required for dimerization of RAF and KSR factors and equivalent to Arg509 of human BRAF is indicated by an asterisk. Note that this arginine is conserved in 100% of RAF and KSR sequences.

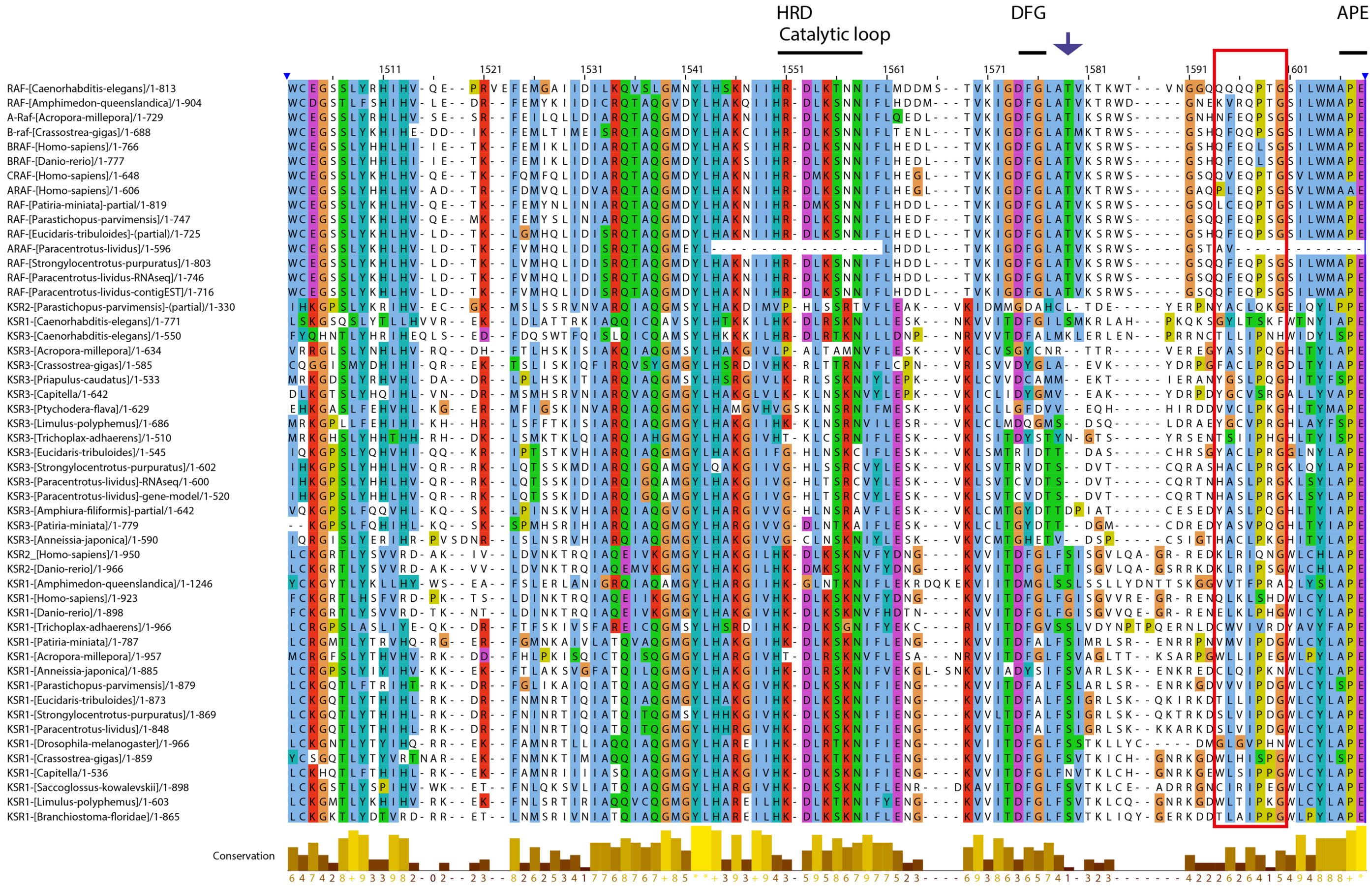


**Supplementary Figure 3.**

**KSR3 factors lack the highly conserved HRD motif involved in binding of the substrate as well as the DFG motif of the activation loop.**

Sequence alignment of RAF and KSR members covering the C-terminal region of the kinase domain and the activation loop. The position of the highly conserved HRD motif, which is part of the catalytic loop is indicated on the top. Note the absence of this motif in KSR3 family members. The DFG motif, located at the basis of the activation loop and that binds to the $Mg^{2+}$ ion that interacts with the ATP molecule and is highly conserved in all kinases is also absent from KSR3 family members, as are absent the serine and threonine residues of the activation segment that are phosphorylated in RAF proteins (Thr491, Ser493 in human C-RAF). Acidic residues present in the activation loop of KSR3 family members and that were mutated to alanine in sea urchin KSR3 are boxed in blue. The region involved in MEK binding in RAF and KSR factors is boxed in red.

.

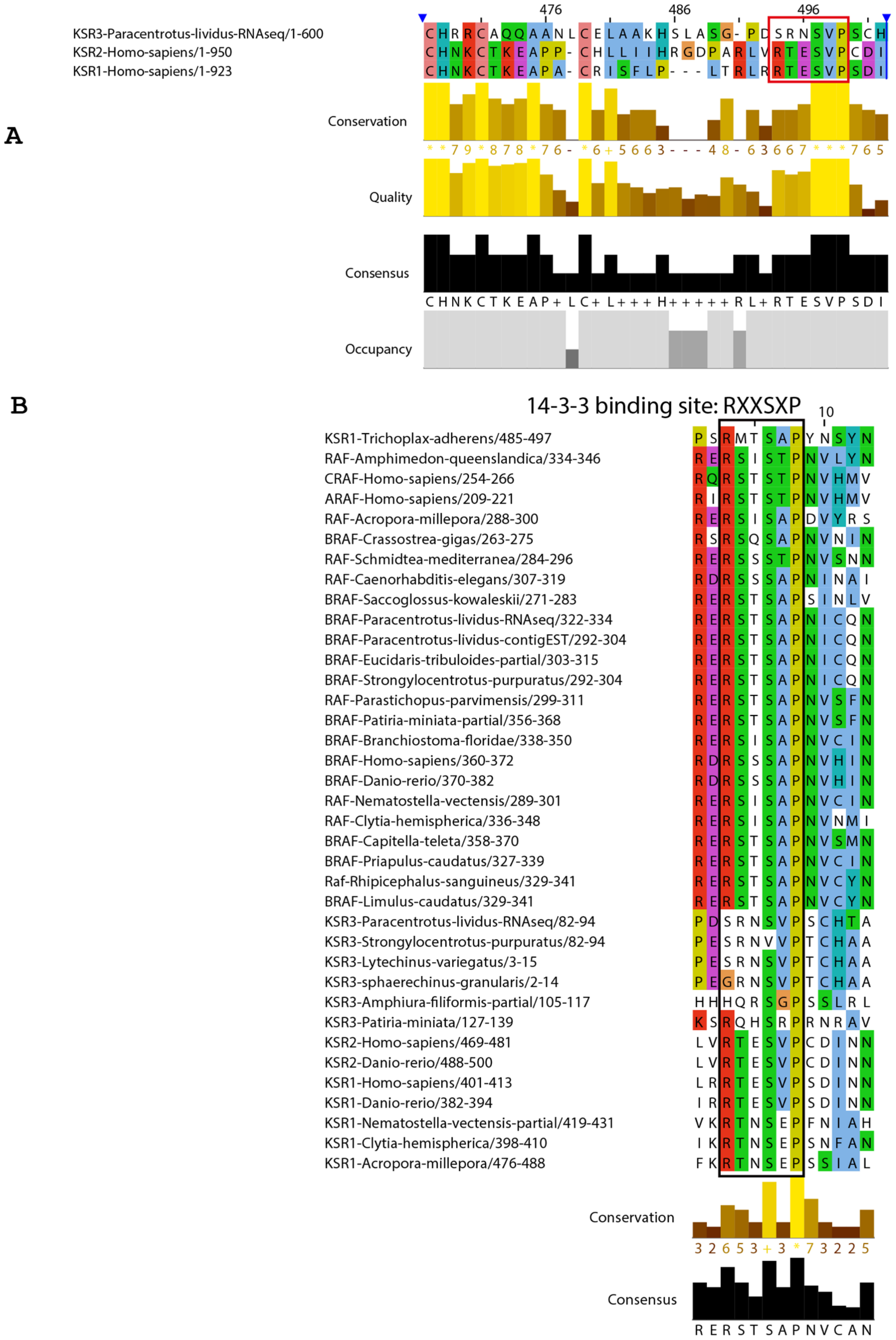


**Supplementary Figure 4.**

**KSR3 proteins contain a putative 14-3-3 binding site after the Cysteine-rich domain.**

A, Sequence alignment between sea urchin KSR3, and human KSR3 and KSR1. The sea urchin sequence contains an atypical 14-3-3 binding RXSXP compared to the canonical 14-3-3 binding site consensus RXXSXP. B, Sequence alignment of the N-terminal 14-3-3 binding site from RAF, KSR1 and KSR3 sequences. Although the N-terminal 14-3-3 binding site appears to be present in RAF sequences from all species, it is only present in KSR3 from echinoderms and in KSR1 from cidarians and vertebrates.

## Consensus for 14-3-3 binding site: RXXSXP

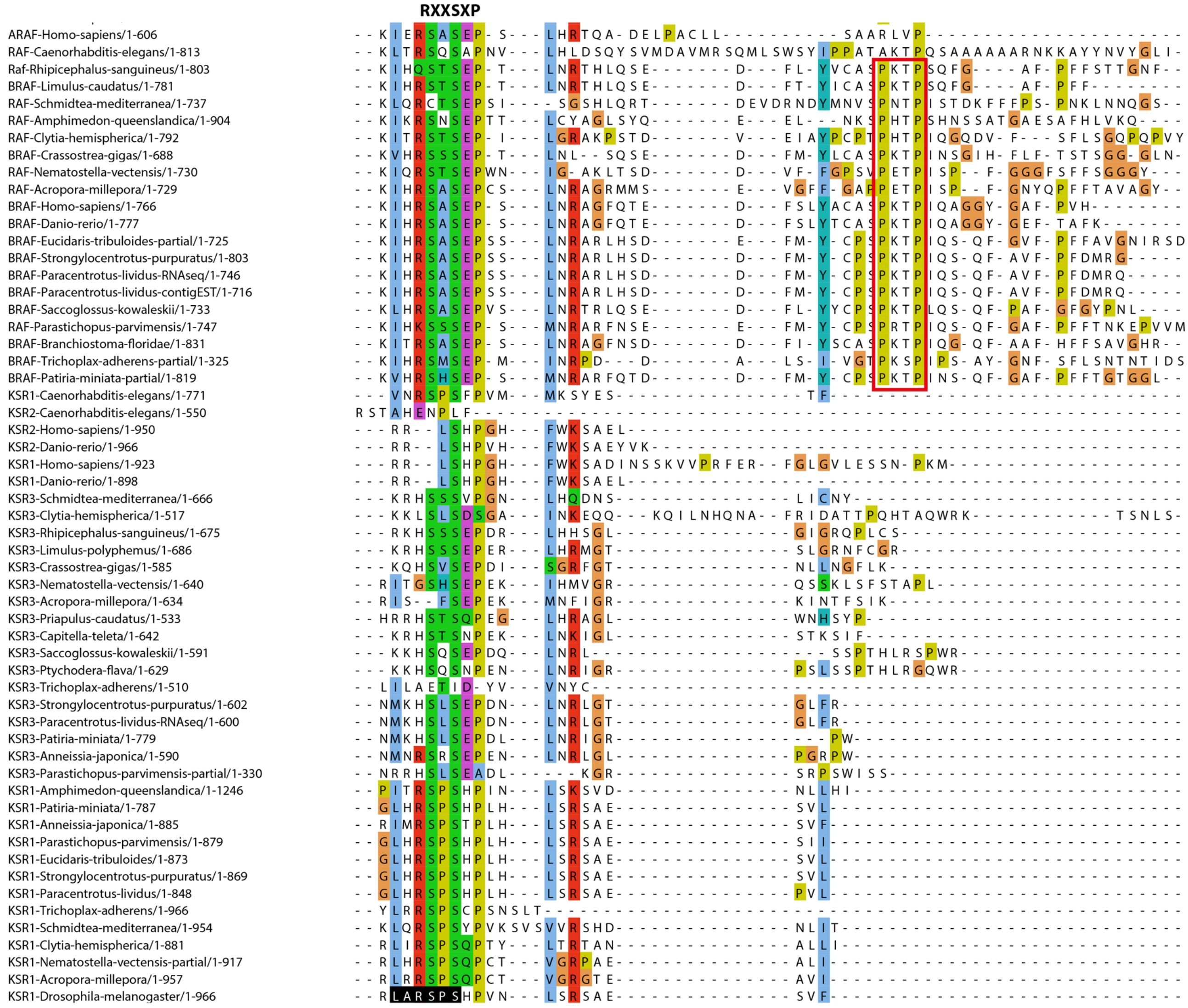


**Supplementary Figure 5.**

**KSR3 factors possess an atypical 14-3-3 binding site near the C-terminus.**

Sequence alignment of the C-terminal region of RAF, KSR3 and KSR1 factors. While all RAF and KSR1 sequences contain a prototypical 14-3-3 binding site (RXXSXP), most KSR3 proteins, with the exception of KSR3 from *Trichoplax*, appear to possess a modified HXXSXP. RAF proteins also contain an ERK MAPK consensus phosphorylation site near the C-terminus (boxed in red) that is absent from KSR proteins.

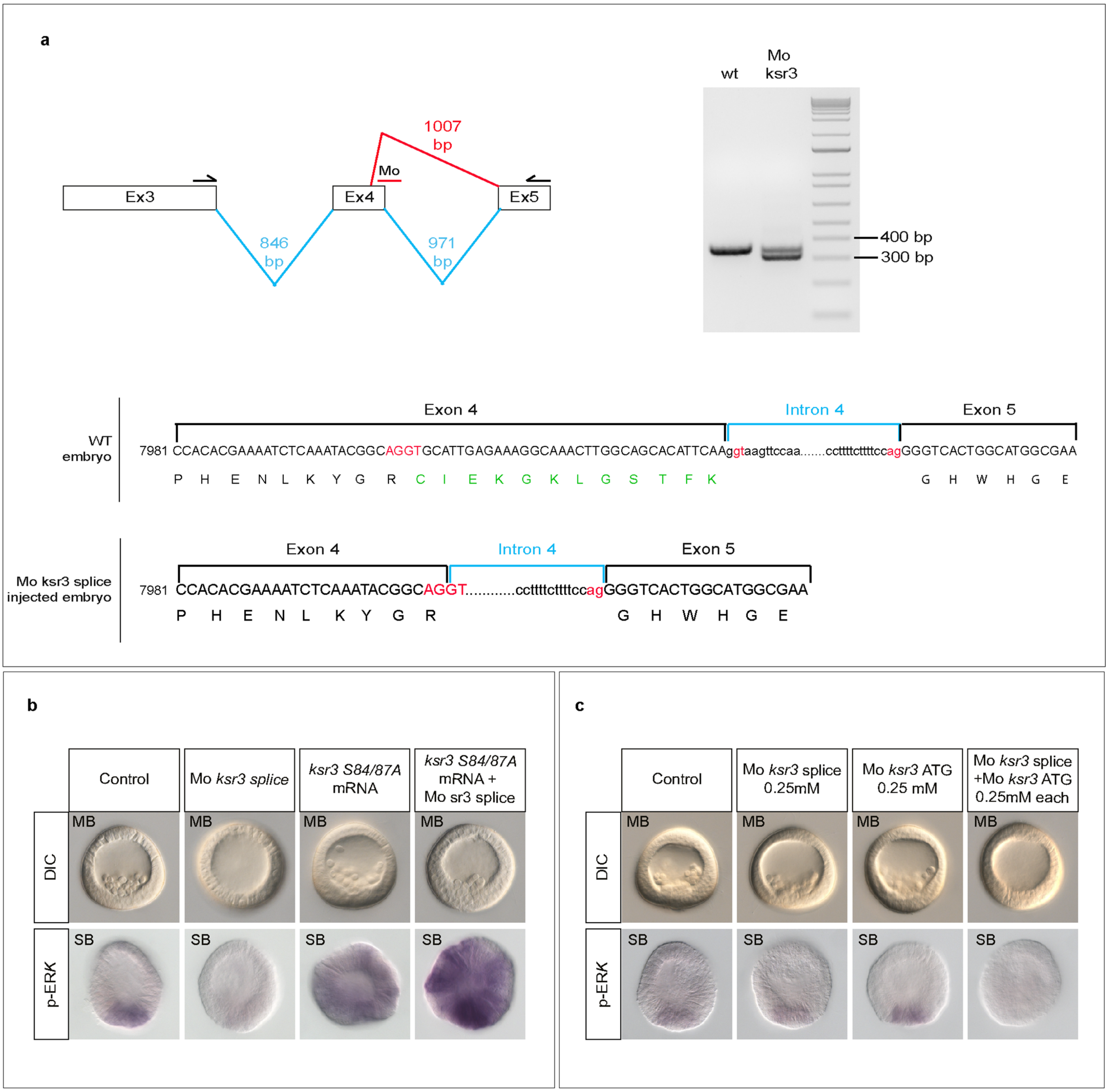


**Supplementary Figure 6**

**Molecular analysis of the effects caused by the splice blocking antisense morpholino oligonucleotide and specificity of the splice blocking and translation blocking morpholinos**

**a,** partial genomic structure of *Paracentrotus lividus ksr3***.** The exon4-intron4 (E4I4) junction targeted by the morpholino is indicated. RT-PCR analysis of *ksr3* mRNA from wild type or morpholino injected embryos. The positions of the forward and reverse primers used for the analysis are indicated. Agarose gel electrophoresis revealed that in the splice blocking morpholino injected embryos, a variant *ksr3* transcript shorter than the wild type fragment is produced. Sequence analysis revealed that this shorter transcript is produced by the utilization of a cryptic donor splice site 36 bp upstream of the normal E4I4 splice junction. This aberrant splicing does not change the reading frame but it causes deletion of the entire P-loop (highlighted in green) including four highly conserved residues (highlighted in

bold).**b**, rescue experiment. While injection of the splice blocking morpholino blocks ERK activation in the PMCs, co-injection of the splice blocking oligonucleotide with mRNA encoding an activated KSR3 protein, rescues ERK activation. **c**, Synergy between the two morpholino oligonucleotides injected at low doses. While single injection of the translation blocking and splice blocking morpholinos at low doses does not block activation of ERK in the PMCs, co-injection of these two morpholinos at these same doses abrogates ERK activation in these cells.

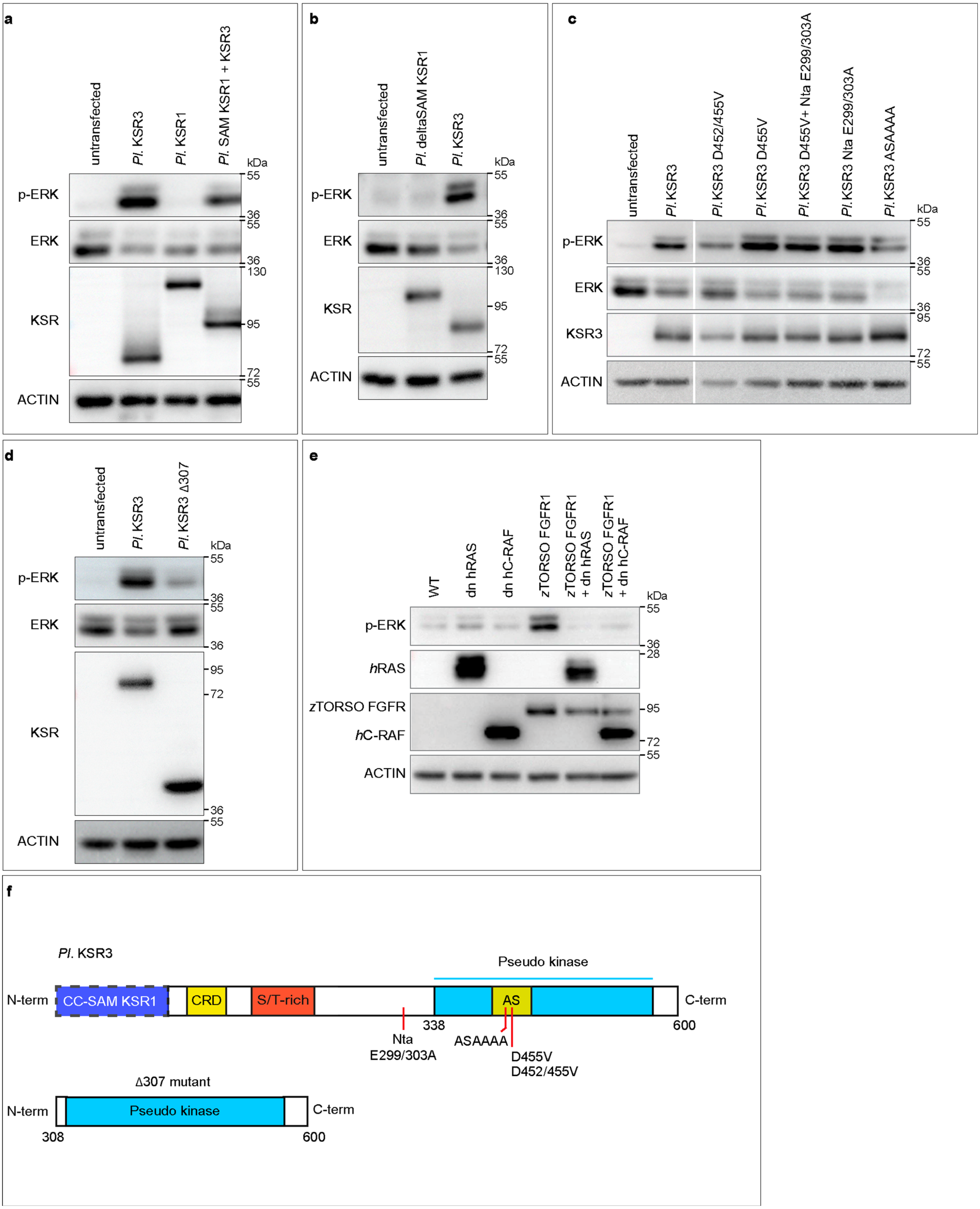


**Supplementary Figure 7.**

**Structural determinants responsible for the constitutive activity of *Paracentrotus lividus* (Pl) KSR3.**

**a**, While KSR3 strongly activates ERK signalling when overexpressed, overexpression of KSR1 does not activate ERK-signalling. Adding back a CC-SAM domain on the N-terminus of KSR3

reduces about 2-fold but does not abrogate the ability of KSR3 to activate ERK signalling suggesting that the absence of the CC-SAM domain in KSR3 is not the main determinant of its constitutive activity [5].

**b**, Deletion of the N-terminal CC-SAM domain of Ksr1 is not sufficient to create a form of KSR1 that activates ERK signalling when overexpressed. **c**, Phosphorylation of the activation segment of B-RAF by a cis-autophosphorylation mechanism is thought to be required in the receiver kinase for its activation [8,49]. Since sea urchin KSR3 contains acidic residues in the activation segment as well as four serine and threonine residues (Extended data Fig. 3), we tested the effects of substituting these residues with valine or alanine. Substitution of the two acidic residues with valine did not block the ability of KSR3 to activate ERK signalling. (Pl KSR3 D452,455V). Similarly, substitution of the four serine or threonine residues present in the activation segment of KSR3 (PL KSR3 AS (AAAA)) with alanine did not suppress the activity of KSR3 consistent with the idea that KSR3 functions as a kinase dead allosteric activator that does not require phosphorylation of the activation segment to be active. The two parts of the blot shown in c originate from the same blot. Phosphorylation of the NtA region is also thought to be important for the activity of RAF and KSR factors. Substitution of two glutamates residues present upstream of the NtA region E299, 303A) for alanine did not eliminate the activity of KSR3. **d**, in contrast deletion of the first 307 amino acids of KSR3 which almost removed the NtA region, leaving only one glutamic acid before the conserved tryptophan at the beginning of the kinase domain, reduced by about 50 % the activity of KSR3 suggesting that phosphorylation of the NtA motif as well as of the region further upstream of the NtA motif is crucial for the ability of KSR3 to activate ERK signalling.

**e**, control experiment showing that the dominant negative mutant form of RAS (Ser17Asn) that was used efficiently blocks activation of ERK mediated by a constitutively active FGFR1 from zebrafish [5] but that it does not block activation ERK mediated by sea urchin KSR3.

A constitutively active chimeric receptor made of the extracellular and transmembrane domains of the *Drosophila* mutant Torso protein (Torso[4021] dominant allele) and the intracellular domain of the zebrafish FGFR1 receptorFGFR1 efficiently activated ERK signaling when overexpressed[10] (lane 4). The constitutive activity of this receptor comes from a mutation the extracellular domain of the Torso protein (Y323C) that results in constitutive dimerization. Co-expression of this mutant Torso-FGFR1 with a dominant negative RAS or dominant negative RAF largely suppressed activation of ERK (lane 5). **f**, scheme describing the position of the different mutants used in this analysis.

| Figure | Experiment | Condition | Number of experiment | Number of embryos | Comments |
|---|---|---|---|---|---|
| **1b** | | dn ras S17N mRNA/ dn raf S621A mRNA | >3 | 15-25 | For each experiment |
| **1b** | Immunostaining P-Erk | dn ras S17N mRNA/dn raf S621A mRNA | 2 | 50 | For each experiment |
| **1c** | Immunostaining P-Erk | time course | >5 | >50 | For each time point/experiment |
| **1c** | ISH ets1 probe | time course | >5 | >50 | For each time point/experiment |
| **1c** | ISH alx1 probe | time course | >5 | >50 | For each time point/experiment |
| **1d** | | Mo ets1 | >5 | 74-100 | For each experiment |
| **1d** | | Mo alx1 | 3 | 50 | For each experiment |
| **1d** | Immunostaining P-Erk | Mo ets1/Mo alx1 | 2 | 25-50 | For each experiment |
| **1d** | ISH msp130 probe | Mo ets1/Mo alx1 | 1 | 20 | |
| **1d** | ISH dri probe | Mo ets1/Mo alx1 | 1 | 30 | |
| **1e** | | *pmar1* mRNA/*ets1* mRNA | >5 | 15-25 | For each experiment |
| **1e** | Immunostaining P-Erk | *pmar1* mRNA | 3 | 15-40 | For each experiment |
| **1e** | Immunostaining P-Erk | *ets1* mRNA | 1 | 20 | |
| **1e** | ISH dri probe | *pmar1* mRNA /*ets1* mRNA | 1 | 35 | |
| **3a** | ISH ksr3 probe | time course | 3 | >50 | For each time point/experiment |
| **3c** | Immunostaining P-Erk | time course | 2 | >50 | For each time point/experiment |
| **3d** | ISH ksr1 probe | time course | 2 | >50 | For each time point/experiment |
| **4a** | | *pmar1* mRNA / Mo hesC/ *dn cadherin* mRNA | >5 | 15-25 | For each experiment |
| **4a** | | *hesC* mRNA | 4 | 15-25 | For each experiment |
| **4a** | ISH ksr3 probe | Mo hesC/*pmar1*/*hesC* / dn cadherin mRNAs | 2 | 50 | For each experiment |
| **4b** | | Mo ksr3 ATG | >5 | >50 | For each experiment |
| **4b** | | Mo ksr3 Splice | 4 | >50 | For each experiment |
| **4b** | | *pmar1/ ksr1 R608H /ksr3 S84/87A* mRNAs | >5 | 15-50 | For each experiment |
| **4b** | | *pmar1* mRNA + Mo ksr3 splice | 3 | 15-50 | For each experiment |
| **4b** | | Mo ksr3 splice + Mo hesC | 2 | 15-50 | For each experiment |
| **4b** | | *ksr3 R364H* mRNA | 5 | 15-50 | For each experiment |
| **4b** | Immunostaining P-Erk | Mo ksr3 ATG | >5 | 50 | For each experiment |
| **4b** | Immunostaining P-Erk | Mo ksr3 splice | 5 | 40 | For each experiment |
| **4b** | Immunostaining P-Erk | *pmar1* mRNA | 2 | 40 | For each experiment |
| **4b** | Immunostaining P-Erk | *pmar1* mRNA + Mo ksr3 splice/Mo ksr3 splice + Mo hesC | 1 | 40 | For each experiment |
| **4b** | Immunostaining P-Erk | *ksr1 R608H /ksr3 R364H /ksr3 S84/87A* mRNAs | 3 | 40 | For each experiment |
| **4b** | ISH/ets1 probe | Mo ksr3 ATG | 1 | 40 | |
| **4b** | ISH/ets1 probe | Mo ksr3 splice | 3 | 30-40 | For each experiment |
| **4b** | ISH /ets1 probe | *pmar1* mRNA/*pmar1* mRNA + Mo ksr3 splice | 2 | 25-30 | For each experiment |
| **4b** | ISH/ets1 probe | Mo ksr3 Splice + Mo hesC | 1 | 35 | |
| **4b** | ISH/ets1 probe | *ksr1 R608H/ksr3 S84/87A* mRNAs | 1 | 40 | For each experiment |
| **4b** | ISH/ets1 probe | *ksr3 R364H* mRNA | 2 | 30-40 | For each experiment |
| **4c** | | *ksr3* mRNA | >5 | 15-50 | For each experiment |
| **4c** | | *pp2aB* mRNA/*pp2aC L209P* mRNA | 2 | 15-25 | For each experiment |
| **4c** | | *ksr3* + *pp2aB* mRNA | 1 | 40 | |
| **4c** | Immunostaining P-Erk | *ksr3* mRNA | >5 | 40-50 | For each experiment |
| **4c** | Immunostaining P-Erk | *pp2aB* mRNA/*pp2aC L209P* mRNA | 2 | 40-50 | For each experiment |
| **4c** | Immunostaining P-Erk | *ksr3* + *pp2aB* mRNA | 1 | 50 | |
| **4d** | | *ksr3 S84 87A* mRNA/*alx1* mRNA | >5 | 15-25 | For each experiment |
| **4d** | | *ksr3 S84 87A* + *alx1* mRNA | 1 | 40 | |
| **6a** | Western blot | *Pl.* KSR1/*Pl*. KSR3 | >5 | | For each experiment |
| **6c** | Western blot | *Pl.* KSR3/ *Sk*. KSR3/ *Nv*. KSR3 | >5 | | For each experiment |
| **6d** | Western blot | *Pl.* KSR3/ *Nv.* KSR3/ *Sk.* KSR3/ dn-*h*RAS / dn-*h*cRAF | >5 | | For each experiment |
| **6d** | Western blot | *Pl.* KSR3 + dn-*h*RAS | 5 | | |
| **6d** | Western blot | *Nv.* KSR3+dn-*h*RAS | 4 | | |
| **6d** | Western blot | *Sk.* KSR3+dn-*h*RAS | 3 | | |
| **6d** | Western blot | *Pl*.KSR3 or *Nv.* KSR3 or *Sk.* KSR3+dn-*h*cRAF | 3 | | For each experiment |
| **6e** | Western blot | *Pl.* KSR3/*Nv*. KSR3/*Sk*. KSR3 | >5 | | For each experiment |
| **6e** | Western blot | *Pl.* KSR3 R364H | 5 | | |
| **6e** | Western blot | *Nv*. KSR3 R390H | 2 | | |
| **6e** | Western blot | *Sk.* KSR3 R350H | 3 | | |
| **6f** | CoIP + Western blot | *Pl.* RAF/*Pl*.KSR1/*Pl.* KSR3 | 2 | | For each experiment |
| **6f** | CoIP + Western blot | *Pl.* KSR1 + *Pl.* RAF/*Pl.* KSR3 + *Pl*. RAF | 2 | | For each experiment |
| **6f** | CoIP + Western blot | *Pl.* KSR3 R364H/ *Pl.* KSR3 R364H + *Pl*. RAF | 2 | | For each experiment |
| **6f** | CoIP + Western blot | *Pl.* KSR1+ *Pl.* KSR3 + *Pl.* RAF | 2 | | |
| **6f** | CoIP + Western blot | *Pl.* KSR1+ *Pl.* KSR3 R364H + *Pl.* RAF | 2 | | |
| **6g** | Western blot | *Pl.* KSR3/*Nv.* KSR3/*Sk.* KSR3 | >5 | | For each experiment |
| **6g** | Western blot | *Pl.* KSR3 S586A | 4 | | |
| **6g** | Western blot | *Nv.* KSR3 S618A | 3 | | |
| **6g** | Western blot | *Sk*. KSR3 S572A | 2 | | |
| **6h** | Western blot | *Sk.* KSR3/*Pl.* KSR3 | >5 | | For each experiment |
| **6h** | Western blot | *Sk.* KSR3 DE->AA/ *Pl.* KSR3 SE->AA | 3 | | For each experiment |
| **S8a** | Western blot | *Pl.* KSR1/*Pl*.KSR3 | >5 | | For each experiment |
| **S8a** | Western blot | *Pl.* SAM KSR1 + KSR3 | 3 | | |
| **S8b** | Western blot | *Pl.* ∆SAM KSR1 | 3 | | |
| **S8c** | Western blot | *Pl.* KSR3 D452/455V / *Pl*. KSR3 D455V | 2 | | For each experiment |
| **S8c** | Western blot | *Pl*. KSR3 D455V+Nta E299/303A | 2 | | |
| **S8c** | Western blot | *Pl.* KSR3 Nta E299/303A /*Pl.* KSR3 ASAAAA | 2 | | For each experiment |
| **S8d** | Western blot | *Pl*. KSR3 ∆307 | 4 | | |
| **S8e** | Western blot | dn *h*RAS/dn *h*C-RAF | >5 | | For each experiment |
| **S8e** | Western blot | *z*-TORSO FGFR1/*z*-TORSO FGFR1 + dn-RAS | 3 | | For each experiment |
| **S8e** | Western blot | *z*-TORSO FGFR1 + dn-cRAF | 2 | | |

**Supplementary table 1.** Number of western blots performed and of replicates experiments performed in the functional analysis and number of embryos analysed
