# Supplementary material for: RAS-independent ERK activation by constitutively active KSR3 in non-chordate metazoa
Source: Nat Commun. 2023 Jul 5;14:3970. doi: 10.1038/s41467-023-39606-y (PMC10322840; doi:10.1038/s41467-023-39606-y)
Supplement: Supplementary file 3 — Description of Additional Supplementary Files [file 41467_2023_39606_MOESM3_ESM.pdf]

Description of Additional Supplementary Files

File Name: Supplementary Data 1
Description: Full alignment of B-RAF, KSR1 and KSR3 protein sequences from various phyla
