# Supplementary material for: RAS-independent ERK activation by constitutively active KSR3 in non-chordate metazoa
Source: Nat Commun. 2023 Jul 5;14:3970. doi: 10.1038/s41467-023-39606-y (PMC10322840; doi:10.1038/s41467-023-39606-y)
Supplement: Supplementary file 4 — Supplementary Data 1 [file 41467_2023_39606_MOESM4_ESM.pdf]

BRAF-Capitella-teleta/1-419		--	
BRAF-Priapulus-caudatus/1-368		--	
CRAF-Homo-sapiens/1-648		--	
ARAF-Homo-sapiens/1-606		--	
RAF-Caenorhabditis-elegans/1-813		--	
Raf-Rhipicephalus-sanguineus/1-803		--	
BRAF-Limulus-caudatus/1-781		--	
RAF-Ramazzottius-variornatus/1-885		--	
RAF-Schmidtea-mediterranea/1-737		--	
RAF-Amphimedon-queenslandica/1-904		--	
RAF-Clytia-hemispherica/1-792		--	
BRAF-Crassostrea-gigas/1-688		--	
RAF-Nematostella-vectensis/1-730		--	
RAF-Acropora-millepora/1-729		--	
BRAF-Homo-sapiens/1-766		--	
BRAF-Danio-rerio/1-777		--	
BRAF-Eucidaris-tribuloides-partial/1-725		--	
BRAF-Strongylocentrotus-purpuratus/1-803		--	
BRAF-Paracentrotus-lividus-RNAseq/1-746		--	
BRAF-Paracentrotus-lividus-contigEST/1-716		--	
BRAF-Saccoglossus-kowaleskii/1-733		--	
RAF-Parastichopus-parvimensis/1-747		--	
BRAF-Branchiostoma-floridae/1-831		--	
BRAF-Trichoplax-adherens-partial/1-325		--	
BRAF-Patiria-miniata-partial/1-819		--	
KSR1-Caenorhabditis-elegans/1-771	1	--MMQTQVASRAGYSNLP	16
KSR2-Caenorhabditis-elegans/1-550		--	
KSR1-Amphimedon-queenslandica/1-1246	1	---MSATT-ATAVYSS--	12
KSR1-Patiria-miniata/1-787		--	
KSR1-Anneissia-japonica/1-885		--	
KSR1-Parastichopus-parvimensis/1-879		--	
KSR1-Eucidaris-tribuloides/1-873		--	
KSR1-Strongylocentrotus-purpuratus/1-869		--	
KSR1-Paracentrotus-lividus/1-848		--	
KSR1-Trichoplax-adherens/1-966		--	
KSR1-Schmidtea-mediterranea/1-954		--	
KSR1-Priapulus-caudatus-partial/1-248		--	
KSR1-Clytia-hemispherica/1-881		--	
KSR1-Nematostella-vectensis-partial/1-917	1	--HSPK	4
KSR1-Acropora-millepora/1-957	1	MEALDFFHRLVSVARPRQRRYRLTSQPAMDVNRSMDSILDNMEGHSKRRSSYSNFE	56
KSR1-Drosophila-melanogaster/1-966	1	---MSSNNNA--------	7
KSR1-Crassostrea-gigas/1-859	1	---MSDS-----------	4
KSR1-Saccoglossus-kowalevskii/1-898		--	
KSR1-Branchiostoma-floridae/1-865	1	---MG-------------	2
KSR1-Capitella-teleta/1-536		--	
KSR1-Rhipicephalus-sanguineus/1-864	1	---MA-------------	2
KSR1-Limulus-polyphemus/1-603	1	---MA-------------	2
KSR3-Ramazzottius-variornatus-partial/1-451		--	
KSR2-Homo-sapiens/1-950	1	---MDE------------	3
KSR2-Danio-rerio/1-966	1	---MDE------------	3
KSR1-Homo-sapiens/1-923	1	----------------------------MDRAALRA---AAMGEKKE---------	16
KSR1-Danio-rerio/1-898		--	
KSR3-Clytia-hemispherica/1-517		--	
KSR3-Schmidtea-mediterranea/1-666		--	
KSR3-Rhipicephalus-sanguineus/1-675		--	
KSR3-Limulus-polyphemus/1-686		--	
KSR3-Limulus-polyphemus-isoformB/1-671		--	
KSR3-Lingula-anatina/1-626		--	
KSR3-Crassostrea-gigas/1-585		--	
KSR3-Nematostella-vectensis/1-640		--	
KSR3-Acropora-millepora/1-634		--	
KSR3-Priapulus-caudatus/1-533		--	
KSR3-Saccoglossus-kowaleskii/1-591		--	
KSR3-Ptychodera-flava/1-629		--	
KSR3-Trichoplax-adherens/1-510		--	
KSR3-Capitella-teleta/1-642		--	
KSR3-Eucidaris-tribuloides/1-545		--	
KSR3-Strongylocentrotus-purpuratus/1-602		--	
KSR3-Paracentrotus-lividus-RNAseq/1-600		--	
KSR3-Notospermus-geniculatus/1-691		--	
KSR3-Aplysia-californica/1-405		--	
KSR3-Amphiura-filiformis-partial/1-642		--	
KSR3-Platynereis-dumerilii/1-704		--	
KSR3-Patiria-miniata/1-779		--	
KSR3-Anneissia-japonica/1-590		--	
KSR3-Parastichopus-parvimensis-partial/1-330		--	

Sequence	Start	Alignment	End
BRAF-Capitella-teleta/1-419		--	
BRAF-Priapulus-caudatus/1-368		--	
CRAF-Homo-sapiens/1-648		--	
ARAF-Homo-sapiens/1-606		--	
RAF-Caenorhabditis-elegans/1-813		--	
Raf-Rhipicephalus-sanguineus/1-803		--	
BRAF-Limulus-caudatus/1-781		--	
RAF-Ramazzottius-variornatus/1-885	1	--MVTKC-----	5
RAF-Schmidtea-mediterranea/1-737		--	
RAF-Amphimedon-queenslandica/1-904		--	
RAF-Clytia-hemispherica/1-792		--	
BRAF-Crassostrea-gigas/1-688		--	
RAF-Nematostella-vectensis/1-730		--	
RAF-Acropora-millepora/1-729		--	
BRAF-Homo-sapiens/1-766		--	
BRAF-Danio-rerio/1-777		--	
BRAF-Eucidaris-tribuloides-partial/1-725		--	
BRAF-Strongylocentrotus-purpuratus/1-803		--	
BRAF-Paracentrotus-lividus-RNAseq/1-746		--	
BRAF-Paracentrotus-lividus-contigEST/1-716		--	
BRAF-Saccoglossus-kowaleskii/1-733		--	
RAF-Parastichopus-parvimensis/1-747		--	
BRAF-Branchiostoma-floridae/1-831		--	
BRAF-Trichoplax-adherens-partial/1-325		--	
BRAF-Patiria-miniata-partial/1-819	1	---SVSPAQSC-----	8
KSR1-Caenorhabditis-elegans/1-771	17	QFGAGIAQDIKTQAIN--------NLKECLKLTT-----INR--------------	45
KSR2-Caenorhabditis-elegans/1-550		--	
KSR1-Amphimedon-queenslandica/1-1246	13	--------SI-GQTQDDKKDLFSVTKGKMKVQQRMIDLTARE-LESTRVSSQSI--	56
KSR1-Patiria-miniata/1-787		--	
KSR1-Anneissia-japonica/1-885	1	---------MLVIMSENGLPSGEKALANLKDIQFLIDQRISQ-LRKLRDLLVRTHP	46
KSR1-Parastichopus-parvimensis/1-879	1	---------MKGPKMSDDPPSDEKQLESLKDLQSVIEGRISR-LEGLRKLNVSS--	44
KSR1-Eucidaris-tribuloides/1-873	1	--------------MSEECL-TDGKALEIKNIQSVIDGRIAH-LDGLRKLP--A--	36
KSR1-Strongylocentrotus-purpuratus/1-869	1	--------------MSGESL-SSLQLMQLQDMQSVIEVRITN-LNGLRKLP--S--	36
KSR1-Paracentrotus-lividus/1-848	1	--------------MSGDC------LSSLQDMQSVIEVRITN-LDGLRKLP--S--	31
KSR1-Trichoplax-adherens/1-966	1	------------MDDRQPAKLLKERLEDLGDLQILIDTTVTQ-LIRTRAQC-----	38
KSR1-Schmidtea-mediterranea/1-954	1	--------------MHDEIT---SSYRTCKDLQSLINITFNDVIKPLREAFKS---	36
KSR1-Priapulus-caudatus-partial/1-248		--	
KSR1-Clytia-hemispherica/1-881	1	---------------------MEEEVATLEMLQKVVDMKIQH-LKGVRDKQHH---	31
KSR1-Nematostella-vectensis-partial/1-917	5	LTAARST---EYLEMSVGTD---DILDQLQMVQRMIDMSAAK-LNSLRTEG-----	48
KSR1-Acropora-millepora/1-957	57	LRAIKRLQTLKMSSNGLAVS---KVLEQIQMVQGMIDMNASS-LNSLRTQF-----	103
KSR1-Drosophila-melanogaster/1-966	8	---PASAPDTGSTNANDPISGSLSVDSNLVIIQDMIDLSANH-LEGLRTQC-----	54
KSR1-Crassostrea-gigas/1-859	5	-----------SDAENEVIEK---TLKNVEFIQTMIDSTAKL-LLGLRNEC-----	40
KSR1-Saccoglossus-kowalevskii/1-898	1	-----------MSGGDPTPKG---AWFECLTIQTVINSQLSL-LAGLRTQC-----	36
KSR1-Branchiostoma-floridae/1-865	3	-----------TEEREEAVKK---ALESCKTLQSMIDISASN-LNGLRTMC-----	38
KSR1-Capitella-teleta/1-536		--	
KSR1-Rhipicephalus-sanguineus/1-864	3	-----------DAEAENKVKS---AIEVCATAQAMIDINSEH-LEGLRTRC-----	38
KSR1-Limulus-polyphemus/1-603	3	-----------EDDSDINVRK---AIESCCNAQAMIDINAVH-LDGLRTLC-----	38
KSR3-Ramazzottius-variornatus-partial/1-451		--	
KSR2-Homo-sapiens/1-950	4	-------ENM-TKSEEQQPLSLQKALQQCELVQNMIDLSISN-LEGLRTKC-----	45
KSR2-Danio-rerio/1-966	4	-------ENM-TKSEE-HHLSLQKALQQCELVQNMIDISISS-LEGLRTKC-----	44
KSR1-Homo-sapiens/1-923	17	---GGGGGDA-AAAEGGAGAAASRALQQCGQLQKLIDISIGS-LRGLRTKC-----	62
KSR1-Danio-rerio/1-898		--	
KSR3-Clytia-hemispherica/1-517		--	
KSR3-Schmidtea-mediterranea/1-666		--	
KSR3-Rhipicephalus-sanguineus/1-675		--	
KSR3-Limulus-polyphemus/1-686		--	
KSR3-Limulus-polyphemus-isoformB/1-671		--	
KSR3-Lingula-anatina/1-626		--	
KSR3-Crassostrea-gigas/1-585		--	
KSR3-Nematostella-vectensis/1-640		--	
KSR3-Acropora-millepora/1-634		--	
KSR3-Priapulus-caudatus/1-533		--	
KSR3-Saccoglossus-kowaleskii/1-591		--	
KSR3-Ptychodera-flava/1-629		--	
KSR3-Trichoplax-adherens/1-510		--	
KSR3-Capitella-teleta/1-642		--	
KSR3-Eucidaris-tribuloides/1-545		--	
KSR3-Strongylocentrotus-purpuratus/1-602		--	
KSR3-Paracentrotus-lividus-RNAseq/1-600		--	
KSR3-Notospermus-geniculatus/1-691		--	
KSR3-Aplysia-californica/1-405		--	
KSR3-Amphiura-filiformis-partial/1-642		--	
KSR3-Platynereis-dumerilii/1-704		--	
KSR3-Patiria-miniata/1-779		--	
KSR3-Anneissia-japonica/1-590		--	
KSR3-Parastichopus-parvimensis-partial/1-330		--	

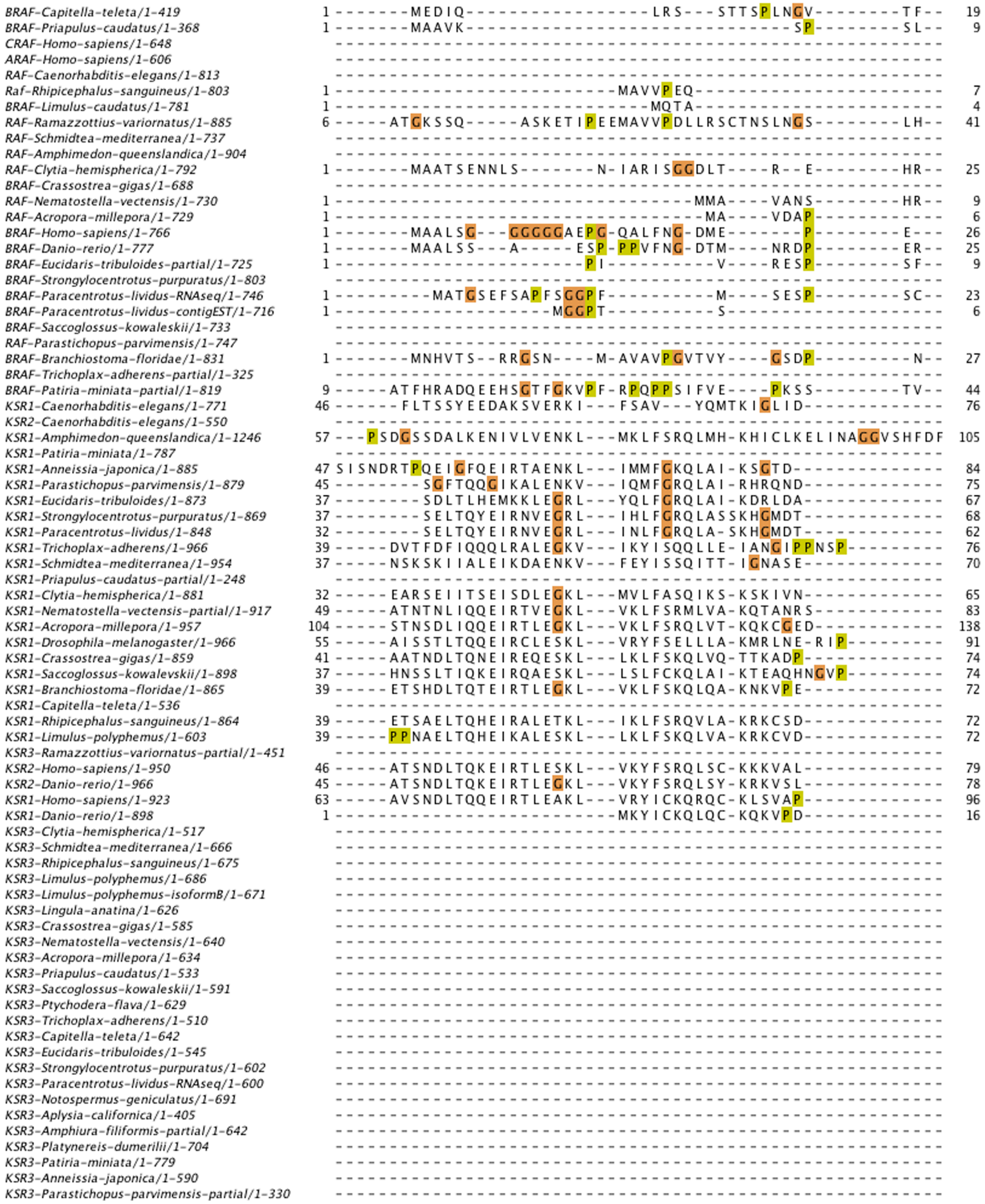
BRAF-Capitella-teleta/1-419
BRAF-Priapulus-caudatus/1-368
CRAF-Homo-sapiens/1-648
ARAF-Homo-sapiens/1-606
RAF-Caenorhabditis-elegans/1-813
Raf-Rhipicephalus-sanguineus/1-803
BRAF-Limulus-caudatus/1-781
RAF-Ramazzottius-variornatus/1-885
RAF-Schmidtea-mediterranea/1-737
RAF-Amphimedon-queenslandica/1-904
RAF-Clytia-hemispherica/1-792
BRAF-Crassostrea-gigas/1-688
RAF-Nematostella-vectensis/1-730
RAF-Acropora-millepora/1-729
BRAF-Homo-sapiens/1-766
BRAF-Danio-rerio/1-777
BRAF-Eucidaris-tribuloides-partial/1-725
BRAF-Strongylocentrotus-purpuratus/1-803
BRAF-Paracentrotus-lividus-RNAseq/1-746
BRAF-Paracentrotus-lividus-contigEST/1-716
BRAF-Saccoglossus-kowaleskii/1-733
RAF-Parastichopus-parvimensis/1-747
BRAF-Branchiostoma-floridae/1-831
BRAF-Trichoplax-adherens-partial/1-325
BRAF-Patiria-miniata-partial/1-819
KSR1-Caenorhabditis-elegans/1-771
KSR2-Caenorhabditis-elegans/1-550
KSR1-Amphimedon-queenslandica/1-1246
KSR1-Patiria-miniata/1-787
KSR1-Anneissia-japonica/1-885
KSR1-Parastichopus-parvimensis/1-879
KSR1-Eucidaris-tribuloides/1-873
KSR1-Strongylocentrotus-purpuratus/1-869
KSR1-Paracentrotus-lividus/1-848
KSR1-Trichoplax-adherens/1-966
KSR1-Schmidtea-mediterranea/1-954
KSR1-Priapulus-caudatus-partial/1-248
KSR1-Clytia-hemispherica/1-881
KSR1-Nematostella-vectensis-partial/1-917
KSR1-Acropora-millepora/1-957
KSR1-Drosophila-melanogaster/1-966
KSR1-Crassostrea-gigas/1-859
KSR1-Saccoglossus-kowalevskii/1-898
KSR1-Branchiostoma-floridae/1-865
KSR1-Capitella-teleta/1-536
KSR1-Rhipicephalus-sanguineus/1-864
KSR1-Limulus-polyphemus/1-603
KSR3-Ramazzottius-variornatus-partial/1-451
KSR2-Homo-sapiens/1-950
KSR2-Danio-rerio/1-966
KSR1-Homo-sapiens/1-923
KSR1-Danio-rerio/1-898
KSR3-Clytia-hemispherica/1-517
KSR3-Schmidtea-mediterranea/1-666
KSR3-Rhipicephalus-sanguineus/1-675
KSR3-Limulus-polyphemus/1-686
KSR3-Limulus-polyphemus-isoformB/1-671
KSR3-Lingula-anatina/1-626
KSR3-Crassostrea-gigas/1-585
KSR3-Nematostella-vectensis/1-640
KSR3-Acropora-millepora/1-634
KSR3-Priapulus-caudatus/1-533
KSR3-Saccoglossus-kowaleskii/1-591
KSR3-Ptychodera-flava/1-629
KSR3-Trichoplax-adherens/1-510
KSR3-Capitella-teleta/1-642
KSR3-Eucidaris-tribuloides/1-545
KSR3-Strongylocentrotus-purpuratus/1-602
KSR3-Paracentrotus-lividus-RNAseq/1-600
KSR3-Notospermus-geniculatus/1-691
KSR3-Aplysia-californica/1-405
KSR3-Amphiura-filiformis-partial/1-642
KSR3-Platynereis-dumerilii/1-704
KSR3-Patiria-miniata/1-779
KSR3-Anneissia-japonica/1-590
KSR3-Parastichopus-parvimensis-partial/1-330

```
BRAF-Capitella-teleta/1-419                        20 TI-----GTG--------EDSAAGCDQGDPETLEE--EIKNIKSVIRITTQKLVEL 60
BRAF-Priapulus-caudatus/1-368                      10 NGSVPNLSSS-------YGSSLEDVDLFEATTLQD--EVRNIQSVIRLTKENLEQL 56
CRAF-Homo-sapiens/1-648                               --------------------------------------------------------
ARAF-Homo-sapiens/1-606                               --------------------------------------------------------
RAF-Caenorhabditis-elegans/1-813                      --------------------------------------------------------
Raf-Rhipicephalus-sanguineus/1-803                  8 -------PYNGSVPP--LNSPPASYEQDDRVALSG--ELRNIRSMIRLTKENLEAL 52
BRAF-Limulus-caudatus/1-781                         5 -------GAN-------------NMTLTVCSPPVE--ELQNIRSMIRLTRGNLEAL 38
RAF-Ramazzottius-variornatus/1-885                 42 SLSIDSMSDSLD-----PE-DPVTIPMTLADRVAE--EVRNYSLMIEVTRGSLEML 89
RAF-Schmidtea-mediterranea/1-737                    1 -----------------MSSKILQERYDLIQKKVS--EFQLINKVLQNKI---IHL 34
RAF-Amphimedon-queenslandica/1-904                  1 ---------------MNMESAQ---FLEVHNSSSR--EVQAIRQVIRSTRTTLDEF 36
RAF-Clytia-hemispherica/1-792                      26 SLTIDNLNCGAEVDTGHFQSNG---I--RASQILS--EAQNVEELFQLTSEQLQEM 74
BRAF-Crassostrea-gigas/1-688                        1 ----------------------------MKIPRIC--AIRNIRKVIQLTKDSLDEL 26
RAF-Nematostella-vectensis/1-730                   10 D------------HPCAFNGEE---NEDISNSHES--EVRTVQHVIRLTKENLDQL 48
RAF-Acropora-millepora/1-729                        7 -------------QTLHFE-DF---D-QNCTNHEV--EVNSLQKVIKLTKENLDKI 42
BRAF-Homo-sapiens/1-766                            27 ------AG------AGAGA-AA---SSAADPAIPE--EVWNIKQMIKLTQEHIEAL 64
BRAF-Danio-rerio/1-777                             26 DPGLDELGAGLE-PACPGE-AA---IPECQQGIPE--EIWNIKQMIKLTQEHLEAL 74
BRAF-Eucidaris-tribuloides-partial/1-725           10 DNG-------------HSD-YF---SMEESTK-TE--QLRAIQQHMELTKKNIEKL 45
BRAF-Strongylocentrotus-purpuratus/1-803            1 --------------------------MGDRTK-TE--QLNVIHMHMELKKKNIEEL 27
BRAF-Paracentrotus-lividus-RNAseq/1-746            24 DNG-------------HSD-YF---KMADRTK-TD--QLNVIHMHMELKKKNIEEL 59
BRAF-Paracentrotus-lividus-contigEST/1-716          7 ------------------------------GH-IS--RVKCYPHAYGVKKKNIEEL 29
BRAF-Saccoglossus-kowaleskii/1-733                    -------------------------------------------------------M 1
RAF-Parastichopus-parvimensis/1-747                 1 ------------------------------------------MQHMVLARKNIEEL 14
BRAF-Branchiostoma-floridae/1-831                  28 LQG-------------HQD-EE---IMDEVQNVRK--VI-------NLTRQNLEAL 57
BRAF-Trichoplax-adherens-partial/1-325                --------------------------------------------------------
BRAF-Patiria-miniata-partial/1-819                 45 THG-------------LY-------IATVLQRTGR--TMFLLATSQKTPSRHLAPA 78
KSR1-Caenorhabditis-elegans/1-771                  77 ------REKR---EINAIWFTFVGLSAQNIRHLEI-CSITDFNALFSITNQELRSL 122
KSR2-Caenorhabditis-elegans/1-550                     -------------------------------------------------------M 1
KSR1-Amphimedon-queenslandica/1-1246              106 EQTNDELDSL---ISFKMFLTVTGLEELSVKSLSSEKEKLSVDRLFQMTPQELQYL 158
KSR1-Patiria-miniata/1-787                          1 --------------------------------MLD--KLQDLSELLEMTEYDIRAQ 22
KSR1-Anneissia-japonica/1-885                      85 FQGTSVESDY---PSIADWMCVVNIRQDVLQKLCQ--PYGTLKGLLDIEDSEMREL 135
KSR1-Parastichopus-parvimensis/1-879               76 NEINEELEQF---PNFQMWLYVMDIHDEAKELMAT--KLKTLMALVELPESEVKSL 126
KSR1-Eucidaris-tribuloides/1-873                   68 DLLCEEHKRY---PNFKQWLHVVDIHLEALEMLSD--KLQTLNSLLDMGESETRTI 118
KSR1-Strongylocentrotus-purpuratus/1-869           69 GSGCSELNRY---PNFKQWLHVVDVHPPAAELLME--KVQNLSHLIKHGESEIRTI 119
KSR1-Paracentrotus-lividus/1-848                   63 GSGCSELNRY---PNFKQWLHVVNVHHPASEMLQQ--KLQNLSHLIKLGESEIRTI 113
KSR1-Trichoplax-adherens/1-966                     77 -SIADKFYQY---PKLNQWLEVVGMESSTAKAICG--LQLPLSTMLNMQEEDMRQM 126
KSR1-Schmidtea-mediterranea/1-954                  71 EDSKICMKEF---PKSEICYRCIGLDDDTIQGLLKI-RNISFPDFLGMDPVEVINL 122
KSR1-Priapulus-caudatus-partial/1-248                 --------------------------------------------------------
KSR1-Clytia-hemispherica/1-881                     66 --QEHAARFY---PKLSQFLYVVDIDEEMRETLVN--RCSLVEELFEFSNERLAGI 114
KSR1-Nematostella-vectensis-partial/1-917          84 QVNHVELPSY---PSLVQWLQVVGINEAAIKEFVA--KCGTVEGLLSLPGDKINTI 134
KSR1-Acropora-millepora/1-957                     139 WNHCEKLRYY---PRLEQWLQVVGINEEAIKSLEE--KCNTIEGLLSLSDEKVRAV 189
KSR1-Drosophila-melanogaster/1-966                 92 ANGLVPHTTG---NELRQWLRVVGLSQGTLTACLA--RLTTLEQSLRLSDEEIRQL 142
KSR1-Crassostrea-gigas/1-859                       75 QHTQEKLSGY---PKTDQWLTVVGLPENAVQEILK--RDIKLGTLLEMTEEEVQSL 125
KSR1-Saccoglossus-kowalevskii/1-898                75 SADIRALESY---PKLEQWLEVVDINKTTAKDVCS--RLKKLESLIDLPDTDINKL 125
KSR1-Branchiostoma-floridae/1-865                  73 DQRTSELQRY---PSVKQWLEVVDIQPVAIRGIEN--KKISLEDLLKMTKVEIVSL 123
KSR1-Capitella-teleta/1-536                           --------------------------------------------------------
KSR1-Rhipicephalus-sanguineus/1-864                73 CENASELKNY---PKLLLWLKVVGLTPNSIQGLSN--KFTTFDDMLEKPEQEIKGY 123
KSR1-Limulus-polyphemus/1-603                      73 AGTIQQLTQY---PRLRQWLCVVGICKS---------------------------- 97
KSR3-Ramazzottius-variornatus-partial/1-451           --------------------------------------------------------
KSR2-Homo-sapiens/1-950                            80 QERNAELDGF---PQLRHWFRIVDVRKEVLEEISP--GQLSLEDLLEMTDEQVCET 130
KSR2-Danio-rerio/1-966                             79 QERNAELDGF---PRLGHWFRIVNMRKEVLEEISP--GELTLEALLEMVDDQVCEE 129
KSR1-Homo-sapiens/1-923                            97 GERTPELNSY---PRFSDWLYTFNVRPEVVQEI-P--RDLTLDALLEMNEAKVKET 146
KSR1-Danio-rerio/1-898                             17 TERPAALNSY---PRLEDWLRIINVRPALIEAV-N--VKLSLDALLQMPIIQVKET 66
KSR3-Clytia-hemispherica/1-517                        --------------------------------------------------------
KSR3-Schmidtea-mediterranea/1-666                     --------------------------------------------------------
KSR3-Rhipicephalus-sanguineus/1-675                   --------------------------------------------------------
KSR3-Limulus-polyphemus/1-686                         --------------------------------------------------------
KSR3-Limulus-polyphemus-isoformB/1-671                --------------------------------------------------------
KSR3-Lingula-anatina/1-626                            --------------------------------------------------------
KSR3-Crassostrea-gigas/1-585                          --------------------------------------------------------
KSR3-Nematostella-vectensis/1-640                     --------------------------------------------------------
KSR3-Acropora-millepora/1-634                         --------------------------------------------------------
KSR3-Priapulus-caudatus/1-533                         --------------------------------------------------------
KSR3-Saccoglossus-kowaleskii/1-591                    --------------------------------------------------------
KSR3-Ptychodera-flava/1-629                           --------------------------------------------------------
KSR3-Trichoplax-adherens/1-510                        --------------------------------------------------------
KSR3-Capitella-teleta/1-642                           --------------------------------------------------------
KSR3-Eucidaris-tribuloides/1-545                      --------------------------------------------------------
KSR3-Strongylocentrotus-purpuratus/1-602              --------------------------------------------------------
KSR3-Paracentrotus-lividus-RNAseq/1-600               --------------------------------------------------------
KSR3-Notospermus-geniculatus/1-691                    --------------------------------------------------------
KSR3-Aplysia-californica/1-405                        --------------------------------------------------------
KSR3-Amphiura-filiformis-partial/1-642                --------------------------------------------------------
KSR3-Platynereis-dumerilii/1-704                      --------------------------------------------------------
KSR3-Patiria-miniata/1-779                            --------------------------------------------------------
KSR3-Anneissia-japonica/1-590                         --------------------------------------------------------
KSR3-Parastichopus-parvimensis-partial/1-330          --------------------------------------------------------
```

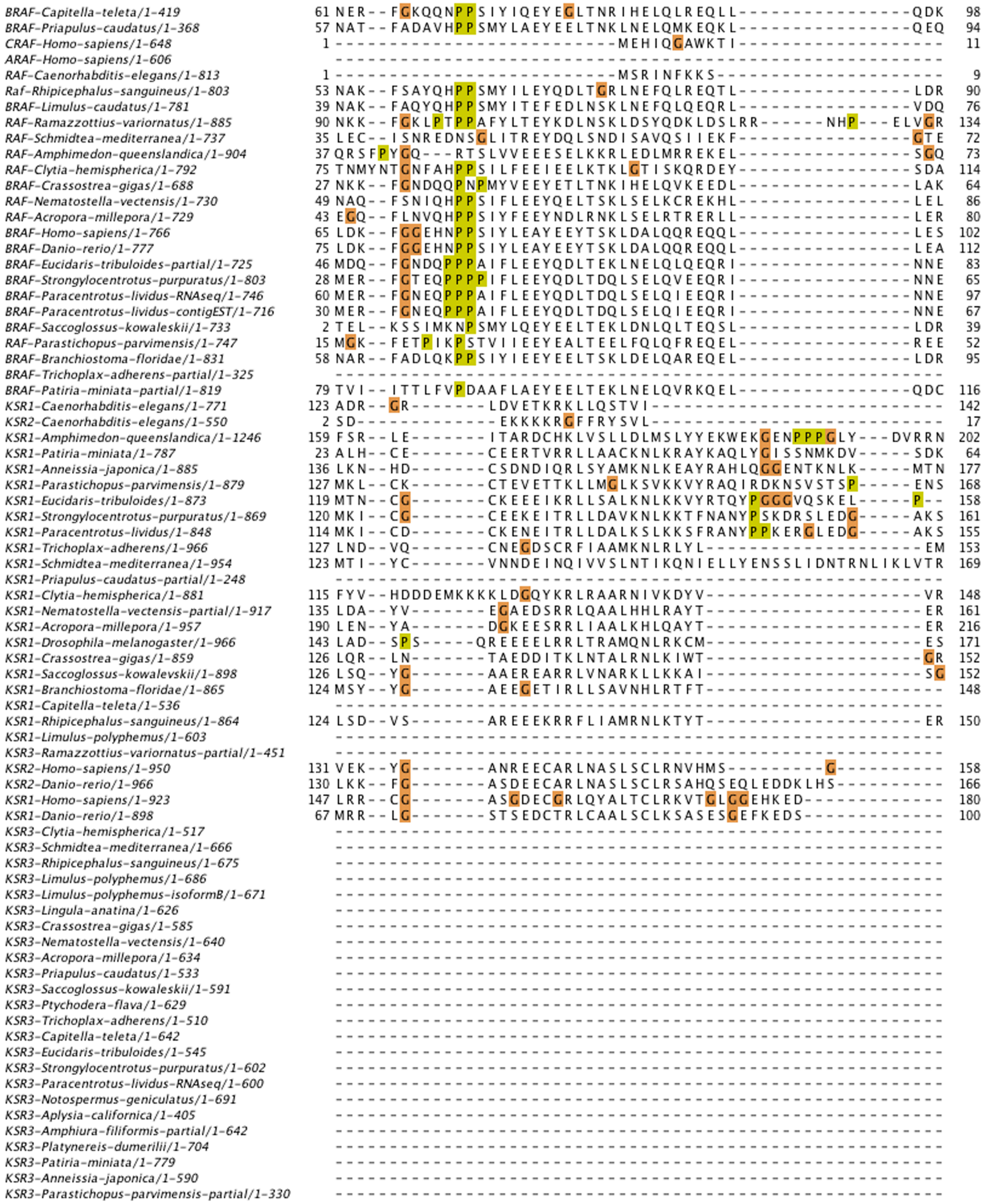
BRAF-Capitella-teleta/1-419
BRAF-Priapulus-caudatus/1-368
CRAF-Homo-sapiens/1-648
ARAF-Homo-sapiens/1-606
RAF-Caenorhabditis-elegans/1-813
Raf-Rhipicephalus-sanguineus/1-803
BRAF-Limulus-caudatus/1-781
RAF-Ramazzottius-variornatus/1-885
RAF-Schmidtea-mediterranea/1-737
RAF-Amphimedon-queenslandica/1-904
RAF-Clytia-hemispherica/1-792
BRAF-Crassostrea-gigas/1-688
RAF-Nematostella-vectensis/1-730
RAF-Acropora-millepora/1-729
BRAF-Homo-sapiens/1-766
BRAF-Danio-rerio/1-777
BRAF-Eucidaris-tribuloides-partial/1-725
BRAF-Strongylocentrotus-purpuratus/1-803
BRAF-Paracentrotus-lividus-RNAseq/1-746
BRAF-Paracentrotus-lividus-contigEST/1-716
BRAF-Saccoglossus-kowaleskii/1-733
RAF-Parastichopus-parvimensis/1-747
BRAF-Branchiostoma-floridae/1-831
BRAF-Trichoplax-adherens-partial/1-325
BRAF-Patiria-miniata-partial/1-819
KSR1-Caenorhabditis-elegans/1-771
KSR2-Caenorhabditis-elegans/1-550
KSR1-Amphimedon-queenslandica/1-1246
KSR1-Patiria-miniata/1-787
KSR1-Anneissia-japonica/1-885
KSR1-Parastichopus-parvimensis/1-879
KSR1-Eucidaris-tribuloides/1-873
KSR1-Strongylocentrotus-purpuratus/1-869
KSR1-Paracentrotus-lividus/1-848
KSR1-Trichoplax-adherens/1-966
KSR1-Schmidtea-mediterranea/1-954
KSR1-Priapulus-caudatus-partial/1-248
KSR1-Clytia-hemispherica/1-881
KSR1-Nematostella-vectensis-partial/1-917
KSR1-Acropora-millepora/1-957
KSR1-Drosophila-melanogaster/1-966
KSR1-Crassostrea-gigas/1-859
KSR1-Saccoglossus-kowalevskii/1-898
KSR1-Branchiostoma-floridae/1-865
KSR1-Capitella-teleta/1-536
KSR1-Rhipicephalus-sanguineus/1-864
KSR1-Limulus-polyphemus/1-603
KSR3-Ramazzottius-variornatus-partial/1-451
KSR2-Homo-sapiens/1-950
KSR2-Danio-rerio/1-966
KSR1-Homo-sapiens/1-923
KSR1-Danio-rerio/1-898
KSR3-Clytia-hemispherica/1-517
KSR3-Schmidtea-mediterranea/1-666
KSR3-Rhipicephalus-sanguineus/1-675
KSR3-Limulus-polyphemus/1-686
KSR3-Limulus-polyphemus-isoformB/1-671
KSR3-Lingula-anatina/1-626
KSR3-Crassostrea-gigas/1-585
KSR3-Nematostella-vectensis/1-640
KSR3-Acropora-millepora/1-634
KSR3-Priapulus-caudatus/1-533
KSR3-Saccoglossus-kowaleskii/1-591
KSR3-Ptychodera-flava/1-629
KSR3-Trichoplax-adherens/1-510
KSR3-Capitella-teleta/1-642
KSR3-Eucidaris-tribuloides/1-545
KSR3-Strongylocentrotus-purpuratus/1-602
KSR3-Paracentrotus-lividus-RNAseq/1-600
KSR3-Notospermus-geniculatus/1-691
KSR3-Aplysia-californica/1-405
KSR3-Amphiura-filiformis-partial/1-642
KSR3-Platynereis-dumerilii/1-704
KSR3-Patiria-miniata/1-779
KSR3-Anneissia-japonica/1-590
KSR3-Parastichopus-parvimensis-partial/1-330

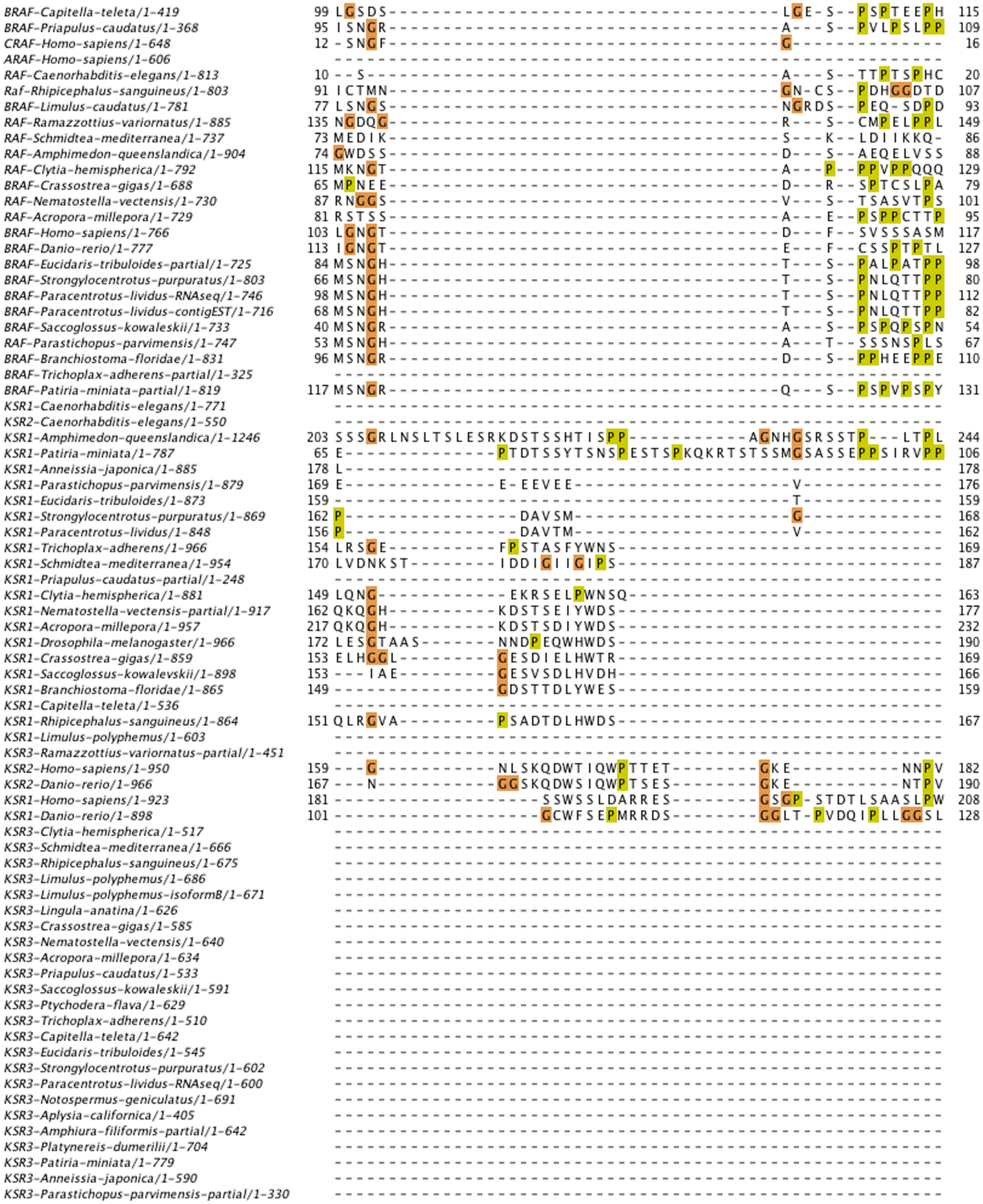
BRAF-Capitella-teleta/1-419 99 LGSDS LGE-S--PSPTEEPH 115
BRAF-Priapulus-caudatus/1-368 95 ISNGR A---S--PVLPSLPP 109
CRAF-Homo-sapiens/1-648 12 -SNGF G 16
ARAF-Homo-sapiens/1-606
RAF-Caenorhabditis-elegans/1-813 10 --S A---S--TTPTSPHC 20
Raf-Rhipicephalus-sanguineus/1-803 91 ICTMN GN-CS--PDHGGDTD 107
BRAF-Limulus-caudatus/1-781 77 LSNGS NGRDS--PEQ-SDPD 93
RAF-Ramazzottius-variornatus/1-885 135 NGDQG R---S--CMPELPPL 149
RAF-Schmidtea-mediterranea/1-737 73 MEDIK S---K--LDIIKKQ- 86
RAF-Amphimedon-queenslandica/1-904 74 GWDSS D---S--AEQELVSS 88
RAF-Clytia-hemispherica/1-792 115 MKNGT A---P--PPVPPQQQ 129
BRAF-Crassostrea-gigas/1-688 65 MPNEE D---R--SPTCSLPA 79
RAF-Nematostella-vectensis/1-730 87 RNGGS V---S--TSASVTPS 101
RAF-Acropora-millepora/1-729 81 RSTSS A---E--PSPPCTTP 95
BRAF-Homo-sapiens/1-766 103 LGNGT D---F--SVSSSASM 117
BRAF-Danio-rerio/1-777 113 IGNGT E---F--CSSPTPTL 127
BRAF-Eucidaris-tribuloides-partial/1-725 84 MSNGH T---S--PALPATPP 98
BRAF-Strongylocentrotus-purpuratus/1-803 66 MSNGH T---S--PNLQTTPP 80
BRAF-Paracentrotus-lividus-RNAseq/1-746 98 MSNGH T---S--PNLQTTPP 112
BRAF-Paracentrotus-lividus-contigEST/1-716 68 MSNGH T---S--PNLQTTPP 82
BRAF-Saccoglossus-kowaleskii/1-733 40 MSNGR A---S--PSPQPSPN 54
RAF-Parastichopus-parvimensis/1-747 53 MSNGH A---T--SSSNSPLS 67
BRAF-Branchiostoma-floridae/1-831 96 MSNGR D---S--PPHEEPPE 110
BRAF-Trichoplax-adherens-partial/1-325
BRAF-Patiria-miniata-partial/1-819 117 MSNGR Q---S--PSPVPSPY 131
KSR1-Caenorhabditis-elegans/1-771
KSR2-Caenorhabditis-elegans/1-550
KSR1-Amphimedon-queenslandica/1-1246 203 SSSGRLNSLTSLESRKDSTSSHTISPP AGNHGSRSSTP---LTPL 244
KSR1-Patiria-miniata/1-787 65 E PTDTSSYTSNSPESTSPKQKRTSTSSMGSASSEPPSIRVPP 106
KSR1-Anneissia-japonica/1-885 178 L 178
KSR1-Parastichopus-parvimensis/1-879 169 E E-EEVEE V 176
KSR1-Eucidaris-tribuloides/1-873 159 T 159
KSR1-Strongylocentrotus-purpuratus/1-869 162 P DAVSM G 168
KSR1-Paracentrotus-lividus/1-848 156 P DAVTM V 162
KSR1-Trichoplax-adherens/1-966 154 LRSGE FPSTASFYWNS 169
KSR1-Schmidtea-mediterranea/1-954 170 LVDNKST IDDIGIIGIPS 187
KSR1-Priapulus-caudatus-partial/1-248
KSR1-Clytia-hemispherica/1-881 149 LQNG EKRSELPWNSQ 163
KSR1-Nematostella-vectensis-partial/1-917 162 QKQGH KDSTSEIYWDS 177
KSR1-Acropora-millepora/1-957 217 QKQGH KDSTSDIYWDS 232
KSR1-Drosophila-melanogaster/1-966 172 LESGTAAS NNDPEQWHWDS 190
KSR1-Crassostrea-gigas/1-859 153 ELHGGL GESDIELHWTR 169
KSR1-Saccoglossus-kowalevskii/1-898 153 ---IAE GESVSDLHVDH 166
KSR1-Branchiostoma-floridae/1-865 149 GDSTTDLYWES 159
KSR1-Capitella-teleta/1-536
KSR1-Rhipicephalus-sanguineus/1-864 151 QLRGVA PSADTDLHWDS 167
KSR1-Limulus-polyphemus/1-603
KSR3-Ramazzottius-variornatus-partial/1-451
KSR2-Homo-sapiens/1-950 159 ---G NLSKQDWTIQWPTTET GKE NNPV 182
KSR2-Danio-rerio/1-966 167 ---N GGSKQDWSIQWPTSES GKE NTPV 190
KSR1-Homo-sapiens/1-923 181 SSWSSLDARRES GSGP-STDTLSAASLPW 208
KSR1-Danio-rerio/1-898 101 GCWFSEPMRRDS GGLT-PVDQIPLLGGSL 128
KSR3-Clytia-hemispherica/1-517
KSR3-Schmidtea-mediterranea/1-666
KSR3-Rhipicephalus-sanguineus/1-675
KSR3-Limulus-polyphemus/1-686
KSR3-Limulus-polyphemus-isoformB/1-671
KSR3-Lingula-anatina/1-626
KSR3-Crassostrea-gigas/1-585
KSR3-Nematostella-vectensis/1-640
KSR3-Acropora-millepora/1-634
KSR3-Priapulus-caudatus/1-533
KSR3-Saccoglossus-kowaleskii/1-591
KSR3-Ptychodera-flava/1-629
KSR3-Trichoplax-adherens/1-510
KSR3-Capitella-teleta/1-642
KSR3-Eucidaris-tribuloides/1-545
KSR3-Strongylocentrotus-purpuratus/1-602
KSR3-Paracentrotus-lividus-RNAseq/1-600
KSR3-Notospermus-geniculatus/1-691
KSR3-Aplysia-californica/1-405
KSR3-Amphiura-filiformis-partial/1-642
KSR3-Platynereis-dumerilii/1-704
KSR3-Patiria-miniata/1-779
KSR3-Anneissia-japonica/1-590
KSR3-Parastichopus-parvimensis-partial/1-330

BRAF-Capitella-teleta/1-419	116	YEISTHTI---GVDSG--	128
BRAF-Priapulus-caudatus/1-368	110	EPPNTPLL-L--	118
CRAF-Homo-sapiens/1-648		--	
ARAF-Homo-sapiens/1-606		--	
RAF-Caenorhabditis-elegans/1-813	21	PSPRLISL-PRCASSS--	35
Raf-Rhipicephalus-sanguineus/1-803	108	TEDAPV----RAS---	116
BRAF-Limulus-caudatus/1-781	94	YDESRMYP-EIAT---	105
RAF-Ramazzottius-variornatus/1-885	150	PPPSRPGG-PALSLVG--	164
RAF-Schmidtea-mediterranea/1-737		--	
RAF-Amphimedon-queenslandica/1-904	89	SSPDGSDQ----SPRG--	100
RAF-Clytia-hemispherica/1-792	130	TSTNSNNS-D-SDS--	141
BRAF-Crassostrea-gigas/1-688	80	STSGGGAQ-P-NI---	90
RAF-Nematostella-vectensis/1-730	102	SSSGPTSP-T-LSS--	113
RAF-Acropora-millepora/1-729	96	SSSGAT--	101
BRAF-Homo-sapiens/1-766	118	DTVTSSS--S-SS---	127
BRAF-Danio-rerio/1-777	128	LDVKGQG--I-QS---	137
BRAF-Eucidaris-tribuloides-partial/1-725	99	PEPVET--	104
BRAF-Strongylocentrotus-purpuratus/1-803	81	PEPADLHV-P-YHNK---	93
BRAF-Paracentrotus-lividus-RNAseq/1-746	113	PELSDPYV-P-YHK--	124
BRAF-Paracentrotus-lividus-contigEST/1-716	83	PELSDPYV-P-YHK--	94
BRAF-Saccoglossus-kowaleskii/1-733	55	IMPNILP----TMI--	64
RAF-Parastichopus-parvimensis/1-747	68	DQEVFVN---	74
BRAF-Branchiostoma-floridae/1-831	111	PPPLPPPP-P-TPSR---	123
BRAF-Trichoplax-adherens-partial/1-325		--	
BRAF-Patiria-miniata-partial/1-819	132	TPEPGPHY-P-HSLKT--	145
KSR1-Caenorhabditis-elegans/1-771		--	
KSR2-Caenorhabditis-elegans/1-550		--	
KSR1-Amphimedon-queenslandica/1-1246	245	TPSSTQSPFQGLVHQNS--TPS-----------------IPTHSV-----------	270
KSR1-Patiria-miniata/1-787	107	DPPPGGYPLRALGRNQYTVTPXXXXXXXXKKRSSDIRAQLCTASVRRETVRRLLAA	162
KSR1-Anneissia-japonica/1-885		--	
KSR1-Parastichopus-parvimensis/1-879		--	
KSR1-Eucidaris-tribuloides/1-873		--	
KSR1-Strongylocentrotus-purpuratus/1-869		--	
KSR1-Paracentrotus-lividus/1-848		--	
KSR1-Trichoplax-adherens/1-966		--	
KSR1-Schmidtea-mediterranea/1-954		--	
KSR1-Priapulus-caudatus-partial/1-248		--	
KSR1-Clytia-hemispherica/1-881		--	
KSR1-Nematostella-vectensis-partial/1-917		--	
KSR1-Acropora-millepora/1-957		--	
KSR1-Drosophila-melanogaster/1-966		--	
KSR1-Crassostrea-gigas/1-859		--	
KSR1-Saccoglossus-kowalevskii/1-898		--	
KSR1-Branchiostoma-floridae/1-865		--	
KSR1-Capitella-teleta/1-536		--	
KSR1-Rhipicephalus-sanguineus/1-864		--	
KSR1-Limulus-polyphemus/1-603		--	
KSR3-Ramazzottius-variornatus-partial/1-451		--	
KSR2-Homo-sapiens/1-950	183	CP-PEPTPWIRTHLS---QSPRVP------------------------------SK	204
KSR2-Danio-rerio/1-966	191	GP-SDHSQWVRLQLS---QSPKLH------------------------------SR	212
KSR1-Homo-sapiens/1-923	209	PP--GSSQLGRAGNSA--QGPRSI------------------------------SV	230
KSR1-Danio-rerio/1-898	129	RP-HSPSPLAR-----------PM------------------------------TT	142
KSR3-Clytia-hemispherica/1-517		--	
KSR3-Schmidtea-mediterranea/1-666		--	
KSR3-Rhipicephalus-sanguineus/1-675		--	
KSR3-Limulus-polyphemus/1-686		--	
KSR3-Limulus-polyphemus-isoformB/1-671		--	
KSR3-Lingula-anatina/1-626		--	
KSR3-Crassostrea-gigas/1-585		--	
KSR3-Nematostella-vectensis/1-640		--	
KSR3-Acropora-millepora/1-634		--	
KSR3-Priapulus-caudatus/1-533		--	
KSR3-Saccoglossus-kowaleskii/1-591		--	
KSR3-Ptychodera-flava/1-629		--	
KSR3-Trichoplax-adherens/1-510		--	
KSR3-Capitella-teleta/1-642		--	
KSR3-Eucidaris-tribuloides/1-545		--	
KSR3-Strongylocentrotus-purpuratus/1-602		--	
KSR3-Paracentrotus-lividus-RNAseq/1-600		--	
KSR3-Notospermus-geniculatus/1-691		--	
KSR3-Aplysia-californica/1-405		--	
KSR3-Amphiura-filiformis-partial/1-642		--	
KSR3-Platynereis-dumerilii/1-704		--	
KSR3-Patiria-miniata/1-779		--	
KSR3-Anneissia-japonica/1-590		--	
KSR3-Parastichopus-parvimensis-partial/1-330		--	

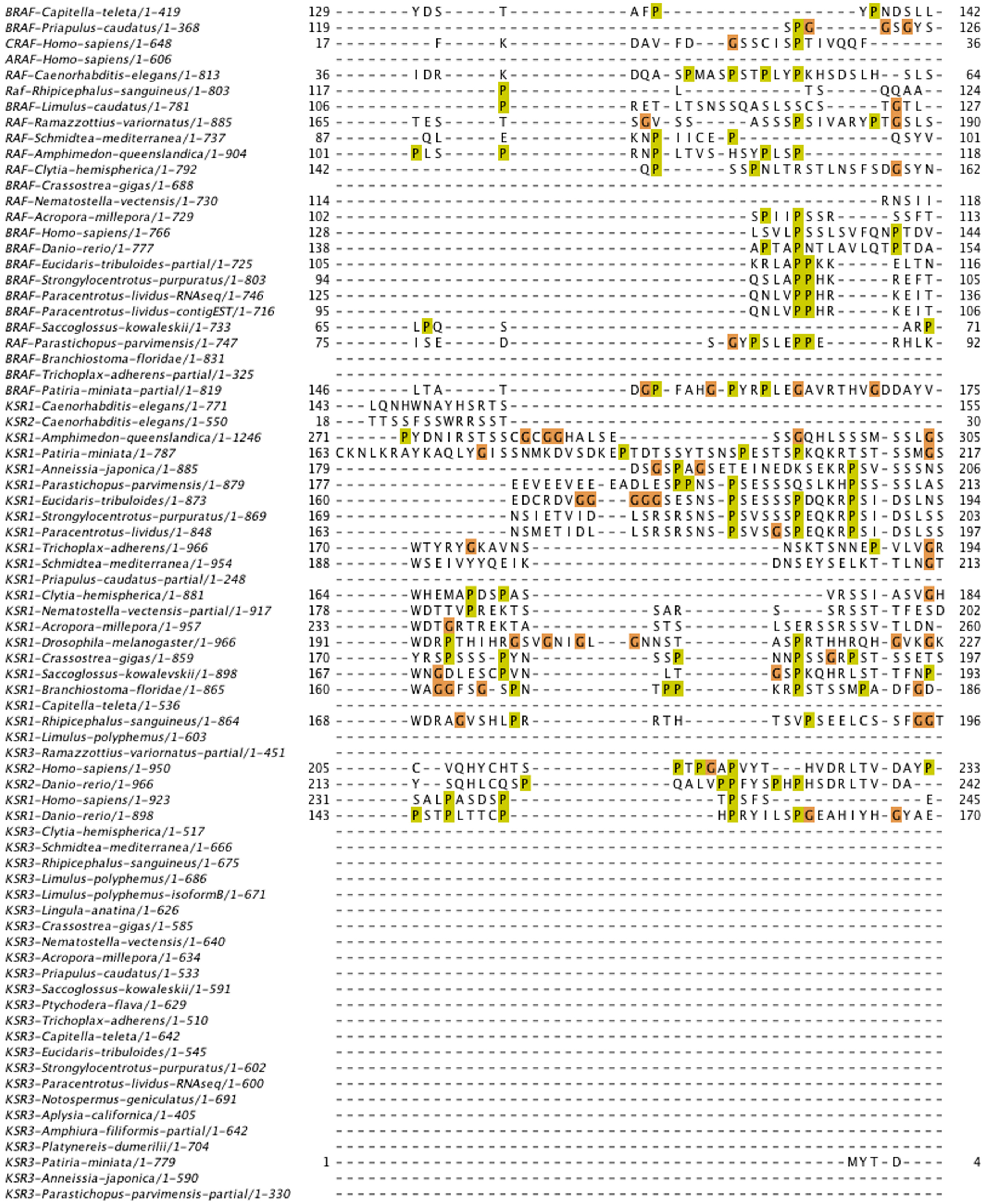
BRAF-Capitella-teleta/1-419
BRAF-Priapulus-caudatus/1-368
CRAF-Homo-sapiens/1-648
ARAF-Homo-sapiens/1-606
RAF-Caenorhabditis-elegans/1-813
Raf-Rhipicephalus-sanguineus/1-803
BRAF-Limulus-caudatus/1-781
RAF-Ramazzottius-variornatus/1-885
RAF-Schmidtea-mediterranea/1-737
RAF-Amphimedon-queenslandica/1-904
RAF-Clytia-hemispherica/1-792
BRAF-Crassostrea-gigas/1-688
RAF-Nematostella-vectensis/1-730
RAF-Acropora-millepora/1-729
BRAF-Homo-sapiens/1-766
BRAF-Danio-rerio/1-777
BRAF-Eucidaris-tribuloides-partial/1-725
BRAF-Strongylocentrotus-purpuratus/1-803
BRAF-Paracentrotus-lividus-RNAseq/1-746
BRAF-Paracentrotus-lividus-contigEST/1-716
BRAF-Saccoglossus-kowaleskii/1-733
RAF-Parastichopus-parvimensis/1-747
BRAF-Branchiostoma-floridae/1-831
BRAF-Trichoplax-adherens-partial/1-325
BRAF-Patiria-miniata-partial/1-819
KSR1-Caenorhabditis-elegans/1-771
KSR2-Caenorhabditis-elegans/1-550
KSR1-Amphimedon-queenslandica/1-1246
KSR1-Patiria-miniata/1-787
KSR1-Anneissia-japonica/1-885
KSR1-Parastichopus-parvimensis/1-879
KSR1-Eucidaris-tribuloides/1-873
KSR1-Strongylocentrotus-purpuratus/1-869
KSR1-Paracentrotus-lividus/1-848
KSR1-Trichoplax-adherens/1-966
KSR1-Schmidtea-mediterranea/1-954
KSR1-Priapulus-caudatus-partial/1-248
KSR1-Clytia-hemispherica/1-881
KSR1-Nematostella-vectensis-partial/1-917
KSR1-Acropora-millepora/1-957
KSR1-Drosophila-melanogaster/1-966
KSR1-Crassostrea-gigas/1-859
KSR1-Saccoglossus-kowalevskii/1-898
KSR1-Branchiostoma-floridae/1-865
KSR1-Capitella-teleta/1-536
KSR1-Rhipicephalus-sanguineus/1-864
KSR1-Limulus-polyphemus/1-603
KSR3-Ramazzottius-variornatus-partial/1-451
KSR2-Homo-sapiens/1-950
KSR2-Danio-rerio/1-966
KSR1-Homo-sapiens/1-923
KSR1-Danio-rerio/1-898
KSR3-Clytia-hemispherica/1-517
KSR3-Schmidtea-mediterranea/1-666
KSR3-Rhipicephalus-sanguineus/1-675
KSR3-Limulus-polyphemus/1-686
KSR3-Limulus-polyphemus-isoformB/1-671
KSR3-Lingula-anatina/1-626
KSR3-Crassostrea-gigas/1-585
KSR3-Nematostella-vectensis/1-640
KSR3-Acropora-millepora/1-634
KSR3-Priapulus-caudatus/1-533
KSR3-Saccoglossus-kowaleskii/1-591
KSR3-Ptychodera-flava/1-629
KSR3-Trichoplax-adherens/1-510
KSR3-Capitella-teleta/1-642
KSR3-Eucidaris-tribuloides/1-545
KSR3-Strongylocentrotus-purpuratus/1-602
KSR3-Paracentrotus-lividus-RNAseq/1-600
KSR3-Notospermus-geniculatus/1-691
KSR3-Aplysia-californica/1-405
KSR3-Amphiura-filiformis-partial/1-642
KSR3-Platynereis-dumerilii/1-704
KSR3-Patiria-miniata/1-779
KSR3-Anneissia-japonica/1-590
KSR3-Parastichopus-parvimensis-partial/1-330

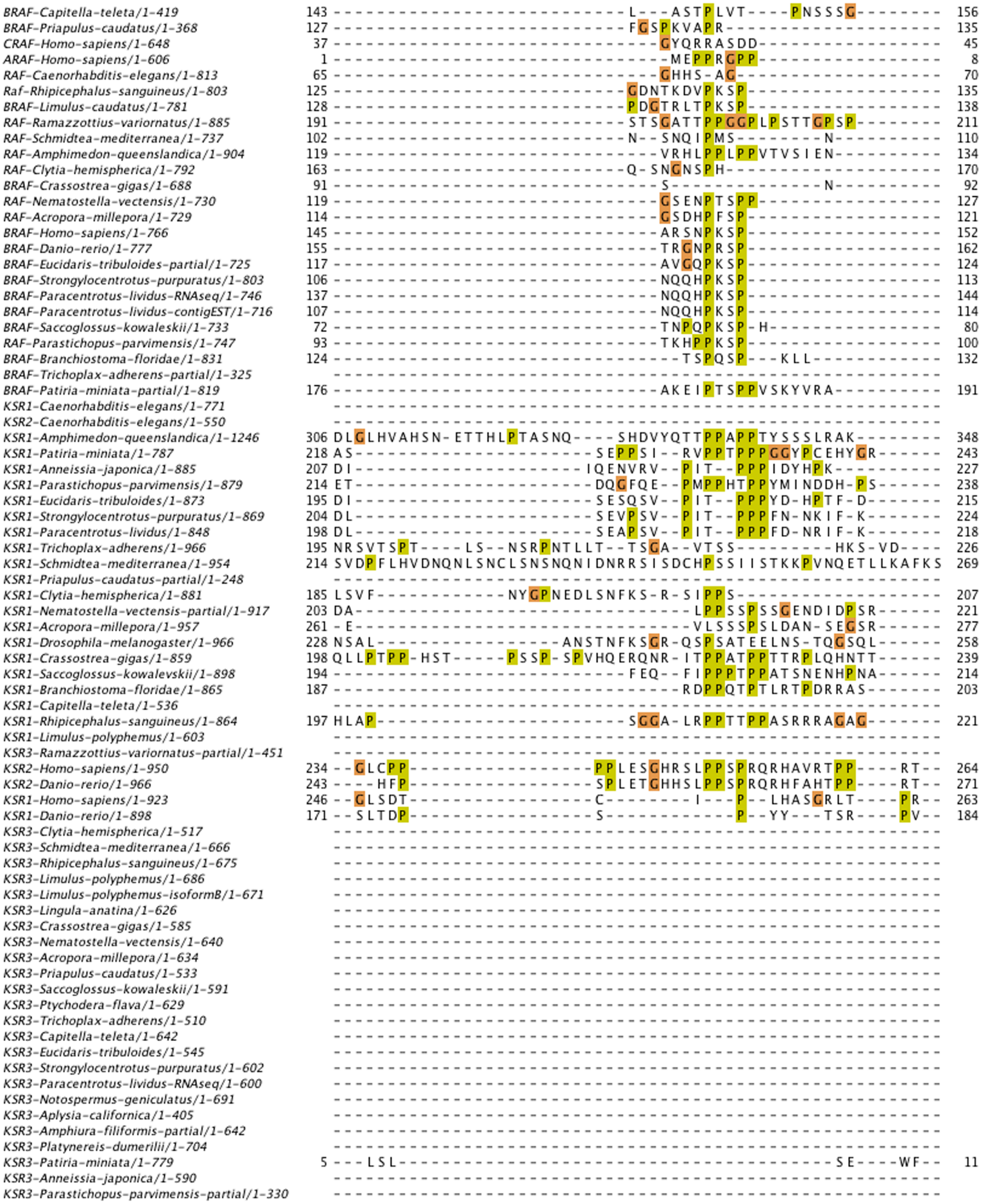
BRAF-Capitella-teleta/1-419
BRAF-Priapulus-caudatus/1-368
CRAF-Homo-sapiens/1-648
ARAF-Homo-sapiens/1-606
RAF-Caenorhabditis-elegans/1-813
Raf-Rhipicephalus-sanguineus/1-803
BRAF-Limulus-caudatus/1-781
RAF-Ramazzottius-variornatus/1-885
RAF-Schmidtea-mediterranea/1-737
RAF-Amphimedon-queenslandica/1-904
RAF-Clytia-hemispherica/1-792
BRAF-Crassostrea-gigas/1-688
RAF-Nematostella-vectensis/1-730
RAF-Acropora-millepora/1-729
BRAF-Homo-sapiens/1-766
BRAF-Danio-rerio/1-777
BRAF-Eucidaris-tribuloides-partial/1-725
BRAF-Strongylocentrotus-purpuratus/1-803
BRAF-Paracentrotus-lividus-RNAseq/1-746
BRAF-Paracentrotus-lividus-contigEST/1-716
BRAF-Saccoglossus-kowaleskii/1-733
RAF-Parastichopus-parvimensis/1-747
BRAF-Branchiostoma-floridae/1-831
BRAF-Trichoplax-adherens-partial/1-325
BRAF-Patiria-miniata-partial/1-819
KSR1-Caenorhabditis-elegans/1-771
KSR2-Caenorhabditis-elegans/1-550
KSR1-Amphimedon-queenslandica/1-1246
KSR1-Patiria-miniata/1-787
KSR1-Anneissia-japonica/1-885
KSR1-Parastichopus-parvimensis/1-879
KSR1-Eucidaris-tribuloides/1-873
KSR1-Strongylocentrotus-purpuratus/1-869
KSR1-Paracentrotus-lividus/1-848
KSR1-Trichoplax-adherens/1-966
KSR1-Schmidtea-mediterranea/1-954
KSR1-Priapulus-caudatus-partial/1-248
KSR1-Clytia-hemispherica/1-881
KSR1-Nematostella-vectensis-partial/1-917
KSR1-Acropora-millepora/1-957
KSR1-Drosophila-melanogaster/1-966
KSR1-Crassostrea-gigas/1-859
KSR1-Saccoglossus-kowalevskii/1-898
KSR1-Branchiostoma-floridae/1-865
KSR1-Capitella-teleta/1-536
KSR1-Rhipicephalus-sanguineus/1-864
KSR1-Limulus-polyphemus/1-603
KSR3-Ramazzottius-variornatus-partial/1-451
KSR2-Homo-sapiens/1-950
KSR2-Danio-rerio/1-966
KSR1-Homo-sapiens/1-923
KSR1-Danio-rerio/1-898
KSR3-Clytia-hemispherica/1-517
KSR3-Schmidtea-mediterranea/1-666
KSR3-Rhipicephalus-sanguineus/1-675
KSR3-Limulus-polyphemus/1-686
KSR3-Limulus-polyphemus-isoformB/1-671
KSR3-Lingula-anatina/1-626
KSR3-Crassostrea-gigas/1-585
KSR3-Nematostella-vectensis/1-640
KSR3-Acropora-millepora/1-634
KSR3-Priapulus-caudatus/1-533
KSR3-Saccoglossus-kowaleskii/1-591
KSR3-Ptychodera-flava/1-629
KSR3-Trichoplax-adherens/1-510
KSR3-Capitella-teleta/1-642
KSR3-Eucidaris-tribuloides/1-545
KSR3-Strongylocentrotus-purpuratus/1-602
KSR3-Paracentrotus-lividus-RNAseq/1-600
KSR3-Notospermus-geniculatus/1-691
KSR3-Aplysia-californica/1-405
KSR3-Amphiura-filiformis-partial/1-642
KSR3-Platynereis-dumerilii/1-704
KSR3-Patiria-miniata/1-779
KSR3-Anneissia-japonica/1-590
KSR3-Parastichopus-parvimensis-partial/1-330

```
BRAF-Capitella-teleta/1-419                    157 ---------------------NLLASPQMPRSPLRNMIKANLPN------------ 179
BRAF-Priapulus-caudatus/1-368                  136 -------------------------------SPLRSFIRAHLPN------------ 148
CRAF-Homo-sapiens/1-648                         46 -------------------------GKLTDPSKTSNTIRVFLPN------------  64
ARAF-Homo-sapiens/1-606                          9 -------------------------ANGAEPSRAVGTVKVYLPN------------  27
RAF-Caenorhabditis-elegans/1-813                71 ---------------------GAGTSDKEPPKFKYKMIMVHLPF------------  93
Raf-Rhipicephalus-sanguineus/1-803             136 ---------------------------------MKSVVRAFLPN------------ 146
BRAF-Limulus-caudatus/1-781                    139 ---------------------------------L-KVVRAFLPY------------ 148
RAF-Ramazzottius-variornatus/1-885             212 ---------------------SASGSTPQTPAIPKKIIMAELPN------------ 234
RAF-Schmidtea-mediterranea/1-737               111 ----------------------HSAATNTTGVSTKCFLRANLPD------------ 132
RAF-Amphimedon-queenslandica/1-904             135 ----------------------DTVAPSSPVTTHAALIRAHLPE------------ 156
RAF-Clytia-hemispherica/1-792                  171 -------------------------------SSLRAHIRAFLPN------------ 183
BRAF-Crassostrea-gigas/1-688                    93 ----------------------SI----PSPKPVHGIIKAYLPN------------ 110
RAF-Nematostella-vectensis/1-730               128 ---------------------------------LRAHVRAFLPN------------ 138
RAF-Acropora-millepora/1-729                   122 -------------------------------QTLRTHIRVNLPN------------ 134
BRAF-Homo-sapiens/1-766                        153 ---------------------------------QKPIVRVFLPN------------ 163
BRAF-Danio-rerio/1-777                         163 ---------------------------------QKPIVRVFLPN------------ 173
BRAF-Eucidaris-tribuloides-partial/1-725       125 ---------------------------------LSQSVRAHLPN------------ 135
BRAF-Strongylocentrotus-purpuratus/1-803       114 ---------------------------------LSQSVRAFLPN------------ 124
BRAF-Paracentrotus-lividus-RNAseq/1-746        145 ---------------------------------LSQSVRAFLPN------------ 155
BRAF-Paracentrotus-lividus-contigEST/1-716     115 ---------------------------------LSQSVRAFLPN------------ 125
BRAF-Saccoglossus-kowaleskii/1-733              81 ----------------------------------ANLTRVCLPN------------  90
RAF-Parastichopus-parvimensis/1-747            101 ----------------------------------NAPVRAYLPN------------ 110
BRAF-Branchiostoma-floridae/1-831              133 ----------------------------------QATVFAHLPN------------ 142
BRAF-Trichoplax-adherens-partial/1-325             --------------------------------------------------------
BRAF-Patiria-miniata-partial/1-819             192 ----------------------SL----PNKQRTSDPLFFAIPN------------ 209
KSR1-Caenorhabditis-elegans/1-771                  --------------------------------------------------------
KSR2-Caenorhabditis-elegans/1-550                  --------------------------------------------------------
KSR1-Amphimedon-queenslandica/1-1246           349 ------------------KKIGHTRSHSNPPLAN-LIMPEAVPENSVPEYRSPSPP 385
KSR1-Patiria-miniata/1-787                     244 --NQYTVTPPTTPKQWVKK----TAAKGTPPPNK-KMITVNLPLPESGLKRSKST- 291
KSR1-Anneissia-japonica/1-885                  228 --LLDSVAPPPTPT-SK-R----TKSKGTPPPSK-KVFNV-HGVILDGSS-SL--- 269
KSR1-Parastichopus-parvimensis/1-879           239 --KRFNVTPPSTPT-IK-K----NKPKGTPPPNK-KLMQL-PPVSQLKRSKSS--- 281
KSR1-Eucidaris-tribuloides/1-873               216 --IPPMSTPPLTPT-SKRV----GKSKGTPPPIK-KLNLP-LTDGQQPIWSSK--- 259
KSR1-Strongylocentrotus-purpuratus/1-869       225 --LPPMPTPPHTPT-FNKR----VKGKTPPLT----RKTN-LPLPAQSNWNSD--- 265
KSR1-Paracentrotus-lividus/1-848               219 --LPPMPTPPHTPT-FSKR----GKGKGTPPPSK-RLNNH-LPLPAPSNWNSV--- 262
KSR1-Trichoplax-adherens/1-966                 227 VVKSSTFS-PSPSSYCIANSLDSGASPTTPQVPA-NYYK--------DSSPSVSD- 271
KSR1-Schmidtea-mediterranea/1-954              270 NTTLSGITCNDDKSYLSTNQLADINSLSVPNTPN------------VQKSRSVGG- 312
KSR1-Priapulus-caudatus-partial/1-248              --------------------------------------------------------
KSR1-Clytia-hemispherica/1-881                 208 ------------------------------PSPA-RSV--------SSIPRSRSD- 223
KSR1-Nematostella-vectensis-partial/1-917      222 ---L---SASDMSDAMVSP----RHS----PRPL-ISPVILQYPVTSGLKRSRSD- 261
KSR1-Acropora-millepora/1-957                  278 ---LSTLSSSDTDTAPSFK----QHS----PLPA-HFI-VRNGSPSSPLKRSVSD- 319
KSR1-Drosophila-melanogaster/1-966             259 ---TLTLTPSPPNS---------------------------------PFTPSSGL- 277
KSR1-Crassostrea-gigas/1-859                   240 -----------------------SRIPSTPPPPS-RIK-VKSPSTAVPITSSKS-- 268
KSR1-Saccoglossus-kowalevskii/1-898            215 ---IWKISPPPTPSSMVKK----GKMPSKGTPPP-KKK-LVVPENNLKRSKSDET- 260
KSR1-Branchiostoma-floridae/1-865              204 ------------------------------DTHD-RTL--------YPSTGDSET- 219
KSR1-Capitella-teleta/1-536                        --------------------------------------------------------
KSR1-Rhipicephalus-sanguineus/1-864            222 ------------------------------GSPS-GSL-IALPDALPPLTKSRSH- 244
KSR1-Limulus-polyphemus/1-603                      --------------------------------------------------------
KSR3-Ramazzottius-variornatus-partial/1-451        --------------------------------------------------------
KSR2-Homo-sapiens/1-950                        265 PNIVTTVTPPGTPPMRKK---NKLKPPGTPPPSS-RKLIHLIPG-FTALHRSKSH- 314
KSR2-Danio-rerio/1-966                         272 PLVVNTMTPPGTPQVRRR---NKLKAPGTPPPAS-RKLIHLLPG-FTALHRSKSH- 321
KSR1-Homo-sapiens/1-923                        264 AL-HSFITPPTTPQLRRH---TKLKPPRTPPPPS-RKVFQLLPS-FPTLTRSKSH- 312
KSR1-Danio-rerio/1-898                         185 RLHGHTSTPPITPPSRRR---HRLKPPCTPPPPS-RKVLHLLPN-I-TLTRSKSH- 233
KSR3-Clytia-hemispherica/1-517                     --------------------------------------------------------
KSR3-Schmidtea-mediterranea/1-666                  --------------------------------------------------------
KSR3-Rhipicephalus-sanguineus/1-675              1 MV--VLCLVY------------------------------SRPV-RPDS-------  16
KSR3-Limulus-polyphemus/1-686                      --------------------------------------------------------
KSR3-Limulus-polyphemus-isoformB/1-671             --------------------------------------------------------
KSR3-Lingula-anatina/1-626                         --------------------------------------------------------
KSR3-Crassostrea-gigas/1-585                       --------------------------------------------------------
KSR3-Nematostella-vectensis/1-640                  --------------------------------------------------------
KSR3-Acropora-millepora/1-634                      --------------------------------------------------------
KSR3-Priapulus-caudatus/1-533                      --------------------------------------------------------
KSR3-Saccoglossus-kowaleskii/1-591               1 ------------------------------------------MS-TEADADSM-Q-  11
KSR3-Ptychodera-flava/1-629                      1 -----------------------------MLFRS-HNIFASRVA-IEAGKTSL-P-  23
KSR3-Trichoplax-adherens/1-510                     --------------------------------------------------------
KSR3-Capitella-teleta/1-642                        --------------------------------------------------------
KSR3-Eucidaris-tribuloides/1-545                 1 -------------------------------------------M-QPKE-------   5
KSR3-Strongylocentrotus-purpuratus/1-602         1 -------------------------------------------M-QPKE-------   5
KSR3-Paracentrotus-lividus-RNAseq/1-600          1 -------------------------------------------M-QPKE-------   5
KSR3-Notospermus-geniculatus/1-691               1 ----------------------------------------------------M-Q-   2
KSR3-Aplysia-californica/1-405                     --------------------------------------------------------
KSR3-Amphiura-filiformis-partial/1-642             --------------------------------------------------------
KSR3-Platynereis-dumerilii/1-704                   --------------------------------------------------------
KSR3-Patiria-miniata/1-779                      12 RL--SWCVVD-----------------QVTPFRQ-HHIFHVLRM-FPEERIHL-Q-  44
KSR3-Anneissia-japonica/1-590                      --------------------------------------------------------
KSR3-Parastichopus-parvimensis-partial/1-330       --------------------------------------------------------
```

Sequence	Start	Alignment	End
BRAF-Capitella-teleta/1-419	180	-----------------------------QQKTCIQV-------------------	187
BRAF-Priapulus-caudatus/1-368	149	-----------------------------QQRTSVSV-------------------	156
CRAF-Homo-sapiens/1-648	65	-----------------------------KQRTVVNV-------------------	72
ARAF-Homo-sapiens/1-606	28	-----------------------------KQRTVVTV-------------------	35
RAF-Caenorhabditis-elegans/1-813	94	-----------------------------DQHSRVEV-------------------	101
Raf-Rhipicephalus-sanguineus/1-803	147	-----------------------------QQRTTVQV-------------------	154
BRAF-Limulus-caudatus/1-781	149	-----------------------------QQRSTVQV-------------------	156
RAF-Ramazzottius-variornatus/1-885	235	-----------------------------NQKTTVEA-------------------	242
RAF-Schmidtea-mediterranea/1-737	133	-----------------------------NQMTSVEV-------------------	140
RAF-Amphimedon-queenslandica/1-904	157	-----------------------------GQRTIVPI-------------------	164
RAF-Clytia-hemispherica/1-792	184	-----------------------------NQRTMIKY-------------------	191
BRAF-Crassostrea-gigas/1-688	111	-----------------------------EQFSLVKI-------------------	118
RAF-Nematostella-vectensis/1-730	139	-----------------------------QQRTMIKC-------------------	146
RAF-Acropora-millepora/1-729	135	-----------------------------QQYTVLKC-------------------	142
BRAF-Homo-sapiens/1-766	164	-----------------------------KQRTVVPA-------------------	171
BRAF-Danio-rerio/1-777	174	-----------------------------KQRTVVPA-------------------	181
BRAF-Eucidaris-tribuloides-partial/1-725	136	-----------------------------QQRTSVLA-------------------	143
BRAF-Strongylocentrotus-purpuratus/1-803	125	-----------------------------QQRTSVLA-------------------	132
BRAF-Paracentrotus-lividus-RNAseq/1-746	156	-----------------------------QQRTSVLA-------------------	163
BRAF-Paracentrotus-lividus-contigEST/1-716	126	-----------------------------QQRTSVLA-------------------	133
BRAF-Saccoglossus-kowaleskii/1-733	91	-----------------------------QQRTTVVA-------------------	98
RAF-Parastichopus-parvimensis/1-747	111	-----------------------------KQRTSVLA-------------------	118
BRAF-Branchiostoma-floridae/1-831	143	-----------------------------HQRTRVQC-------------------	150
BRAF-Trichoplax-adherens-partial/1-325		--	
BRAF-Patiria-miniata-partial/1-819	210	------------------------LMSHYRSHLKVLC-------------------	222
KSR1-Caenorhabditis-elegans/1-771		--	
KSR2-Caenorhabditis-elegans/1-550		--	
KSR1-Amphimedon-queenslandica/1-1246	386	YYNGHAPPTRTGSHPSIIHNSIANSNQSLANSTSTKSHPQVRRLMAGNYNFSR-SI	440
KSR1-Patiria-miniata/1-787	292	--------------E-------ASLPHRIDI--------------EGGGS-----L	307
KSR1-Anneissia-japonica/1-885	270	----------------------PSRTQRVR------------SE------------	279
KSR1-Parastichopus-parvimensis/1-879	282	----------------------EVSPHKVDMSVV--------SDIPGPRK-----F	302
KSR1-Eucidaris-tribuloides/1-873	260	----------------------ST------------------------ES-----V	264
KSR1-Strongylocentrotus-purpuratus/1-869	266	----------------------N--------------------------H-----L	268
KSR1-Paracentrotus-lividus/1-848	263	----------------------D--------------------------H-----L	265
KSR1-Trichoplax-adherens/1-966	272	--------------I---NAGLLENNHNVDEATV--------AQ-DGSNC------	295
KSR1-Schmidtea-mediterranea/1-954	313	--------------S---EEILTDLPNNIVNRST--------EF-SISPTLHRKNY	342
KSR1-Priapulus-caudatus-partial/1-248	1	----------------------------MHVA----------------RF-----F	7
KSR1-Clytia-hemispherica/1-881	224	--------------D-------ANVGQRIHRGST--------SS-AGSSS-----S	244
KSR1-Nematostella-vectensis-partial/1-917	262	--------------E-------ANIGNRIISDVT--------M--RGSAT-----V	281
KSR1-Acropora-millepora/1-957	320	--------------E-------VNLAHRIHIT-N--------E--HASAT-----V	338
KSR1-Drosophila-melanogaster/1-966	278	--------------S-------SS----LNGT-----------------------P	285
KSR1-Crassostrea-gigas/1-859	269	--------------Y-------TNLHTNLHIKVV--------DV-DGINK-----F	289
KSR1-Saccoglossus-kowalevskii/1-898	261	-----------------------SLL--YKVNS------------D---------A	270
KSR1-Branchiostoma-floridae/1-865	220	--------------Y-------ISEDEEFPLSP------------S---------Y	233
KSR1-Capitella-teleta/1-536		--	
KSR1-Rhipicephalus-sanguineus/1-864	245	--------------E-------AHLENRLDLGP------------D---------H	258
KSR1-Limulus-polyphemus/1-603		--	
KSR3-Ramazzottius-variornatus-partial/1-451		--	
KSR2-Homo-sapiens/1-950	315	--------------E-------FQLGHRVDEAHTPKAKKKSKPL------------	337
KSR2-Danio-rerio/1-966	322	--------------E-------FQLGNRIDDAQTPKAKKKNKPL------------	344
KSR1-Homo-sapiens/1-923	313	--------------E-------SQLGNRIDDVSSMR--------------------	327
KSR1-Danio-rerio/1-898	234	--------------E-------SQLGHRIEDTPTNKCVKKNKLF------------	256
KSR3-Clytia-hemispherica/1-517		--	
KSR3-Schmidtea-mediterranea/1-666	1	---------------------------MVDLNQ------DLAYE------------	11
KSR3-Rhipicephalus-sanguineus/1-675		--	
KSR3-Limulus-polyphemus/1-686		--	
KSR3-Limulus-polyphemus-isoformB/1-671		--	
KSR3-Lingula-anatina/1-626	1	----------------------------MDENR-----------------------	5
KSR3-Crassostrea-gigas/1-585		--	
KSR3-Nematostella-vectensis/1-640		--	
KSR3-Acropora-millepora/1-634		--	
KSR3-Priapulus-caudatus/1-533		--	
KSR3-Saccoglossus-kowaleskii/1-591	12	--------------T-------L-----GLEQN-----------------------	18
KSR3-Ptychodera-flava/1-629	24	--------------L-------AKLAQICQIAE-----------------------	35
KSR3-Trichoplax-adherens/1-510		--	
KSR3-Capitella-teleta/1-642		--	
KSR3-Eucidaris-tribuloides/1-545	6	-----------------------HLALIMEQLQ-----------------------	15
KSR3-Strongylocentrotus-purpuratus/1-602	6	-----------------------HIALIMEQLQ-----------------------	15
KSR3-Paracentrotus-lividus-RNAseq/1-600	6	-----------------------HIALIMEQLH-----------------------	15
KSR3-Notospermus-geniculatus/1-691	3	--------------T-------ARMTAADDSVR------SSQYL------------	19
KSR3-Aplysia-californica/1-405		--	
KSR3-Amphiura-filiformis-partial/1-642		--	
KSR3-Platynereis-dumerilii/1-704	1	----------------------------MDVLQ-----------------------	5
KSR3-Patiria-miniata/1-779	45	--------------T-------ADIATIMDILT-----------------------	56
KSR3-Anneissia-japonica/1-590	1	----------------------------MEVFT-----------------------	5
KSR3-Parastichopus-parvimensis-partial/1-330		--	

BRAF-Capitella-teleta/1-419	188	--QDGLTLKEALAKP------------------------------------MKRRE	205
BRAF-Priapulus-caudatus/1-368	157	--RPGVTLREALSRA------------------------------------LKLRK	174
CRAF-Homo-sapiens/1-648	73	--RNGMSLHDCLMKA------------------------------------LKVRG	90
ARAF-Homo-sapiens/1-606	36	--RDGMSVYDSLDKA------------------------------------LKVRG	53
RAF-Caenorhabditis-elegans/1-813	102	--RPGETARDAISKL------------------------------------LKKRN	119
Raf-Rhipicephalus-sanguineus/1-803	155	--RPGQTVMEALSKA------------------------------------MKRRK	172
BRAF-Limulus-caudatus/1-781	157	--RPGQTIQEALSKA------------------------------------MERRH	174
RAF-Ramazzottius-variornatus/1-885	243	--IKGKSVQDTLLKK------------------------------------LQHRQ	260
RAF-Schmidtea-mediterranea/1-737	141	--KPNTSLICALEKK------------------------------------LSNRG	158
RAF-Amphimedon-queenslandica/1-904	165	--RQGVTIQDALMKA------------------------------------MKTRN	182
RAF-Clytia-hemispherica/1-792	192	--KLGQTVREALYKA------------------------------------MSRRS	209
BRAF-Crassostrea-gigas/1-688	119	--EPGKILKEGLKKS------------------------------------MSRRF	136
RAF-Nematostella-vectensis/1-730	147	--QPGVTLKQALDKR------------------------------------MRMRG	164
RAF-Acropora-millepora/1-729	143	--KPGITLKEALDKG------------------------------------MRTRG	160
BRAF-Homo-sapiens/1-766	172	--RCGVTVRDSLKKA------------------------------------LMMRG	189
BRAF-Danio-rerio/1-777	182	--RCGMTVRDSLKKA------------------------------------LMMRG	199
BRAF-Eucidaris-tribuloides-partial/1-725	144	--KAGTTLKESLSKA------------------------------------MKMRE	161
BRAF-Strongylocentrotus-purpuratus/1-803	133	--KSGTTLKYCLSKA------------------------------------MKMRE	150
BRAF-Paracentrotus-lividus-RNAseq/1-746	164	--KSGTTLKHSLSKA------------------------------------MKMRE	181
BRAF-Paracentrotus-lividus-contigEST/1-716	134	--KSGTTLKHSLSKA------------------------------------MKMRE	151
BRAF-Saccoglossus-kowaleskii/1-733	99	--VEGRTLRDSLSKA------------------------------------MQRRG	116
RAF-Parastichopus-parvimensis/1-747	119	--KQGTILRDALSKA------------------------------------MKMRE	136
BRAF-Branchiostoma-floridae/1-831	151	--KSGTTLREALSKA------------------------------------MRMRG	168
BRAF-Trichoplax-adherens-partial/1-325		--	
BRAF-Patiria-miniata-partial/1-819	223	--KPGTTLRDALAKA------------------------------------MKVRE	240
KSR1-Caenorhabditis-elegans/1-771	156	---S---------------GSTDE-----PS----------GQ----S-----TPA	169
KSR2-Caenorhabditis-elegans/1-550	31	---S---------------GSISQ-----SS----------RTTSKTT-----TSS	48
KSR1-Amphimedon-queenslandica/1-1246	441	STDLGDVQPDPPLRSN-----SEAS-----SVQSA--SPRQRRLSKEVGTSTSMVD	484
KSR1-Patiria-miniata/1-787	308	SRIRGSKIP-PLLKKQTSGGSCEAN------------LNSSRRHSEDT---MDRRN	347
KSR1-Anneissia-japonica/1-885	280	--IV------------SPGSSCEAI--------------VSRLKSEDE---SDPGS	304
KSR1-Parastichopus-parvimensis/1-879	303	LNIW------------PPSYSSSSLD-----------KFQPRRHSEDG---TLDRS	332
KSR1-Eucidaris-tribuloides/1-873	265	LPHS------------RPGFDSIISNLKPGQMRRGGHDLPMRRYSDDH---TDAV-	304
KSR1-Strongylocentrotus-purpuratus/1-869	269	NKRS------------GPGYDSATL----GRIQVRSRDQHTRRYSDDQ---SVMR-	304
KSR1-Paracentrotus-lividus/1-848	266	NK---------------PSFDSATL----GRIQGKQRDPRDRRYSDDH---NAMR-	298
KSR1-Trichoplax-adherens/1-966	296	-----NTDA--NSVLQ-------------IIVQPSDAEN-EIQLSNRS---NSLGS	327
KSR1-Schmidtea-mediterranea/1-954	343	SQIPGKTKP--NQTRN-------------IQLQRS------LSHEFDS---KNLLN	374
KSR1-Priapulus-caudatus-partial/1-248	8	SRRTKKKPE--EL-----------------WLGRSLEAL--SQHTIDG---STGSR	39
KSR1-Clytia-hemispherica/1-881	245	TSRY--------------------------MV-----KSEKIRRK-------NKPG	262
KSR1-Nematostella-vectensis-partial/1-917	282	GKARGK-------KLS-------------PVI-----------------------P	294
KSR1-Acropora-millepora/1-957	339	GKKRGR------GKLE-------------PIV----------------------PG	353
KSR1-Drosophila-melanogaster/1-966	286	QRSRGTPPP--ARKHQ-------------TLLSQSHVQV-------DG--------	311
KSR1-Crassostrea-gigas/1-859	290	NKHLITRPS-----HP-------------PLIKS---YSEERRKRPD---------	315
KSR1-Saccoglossus-kowalevskii/1-898	271	NSRNFKKPT--NLKMP-------------GIVGGSHEVLSKRRRSVES---DSDAG	308
KSR1-Branchiostoma-floridae/1-865	234	LTNAGMTPP----STP-------------TILT--------RRPTPPA---T--PP	259
KSR1-Capitella-teleta/1-536		--	
KSR1-Rhipicephalus-sanguineus/1-864	259	NNATSRQPP----TLP-------------NDGG--------LRRRLAT---E--PG	284
KSR1-Limulus-polyphemus/1-603		--	
KSR3-Ramazzottius-variornatus-partial/1-451		--	
KSR2-Homo-sapiens/1-950	338	--NL--------KIHSSV-GSCENI----PSQQRS------PLLSERS-----LRS	367
KSR2-Danio-rerio/1-966	345	--NL--------KIHSSVAGSCENL----PT-QRS------PLHSDHS-----LRS	374
KSR1-Homo-sapiens/1-923		--	
KSR1-Danio-rerio/1-898	257	-LNV--------QIN---GNGCEDS----PS--RS------PTLSVRT-----PGA	283
KSR3-Clytia-hemispherica/1-517		--	
KSR3-Schmidtea-mediterranea/1-666	12	-SLS--------ENSSKVTGS-----------------------------------	23
KSR3-Rhipicephalus-sanguineus/1-675	17	--------------RPRRLRHCHF--------------------------------	26
KSR3-Limulus-polyphemus/1-686		--	
KSR3-Limulus-polyphemus-isoformB/1-671		--	
KSR3-Lingula-anatina/1-626	6	-------------TDSMVQNELEE--------------------------------	16
KSR3-Crassostrea-gigas/1-585		--	
KSR3-Nematostella-vectensis/1-640		--	
KSR3-Acropora-millepora/1-634		--	
KSR3-Priapulus-caudatus/1-533		--	
KSR3-Saccoglossus-kowaleskii/1-591		--	
KSR3-Ptychodera-flava/1-629		--	
KSR3-Trichoplax-adherens/1-510		--	
KSR3-Capitella-teleta/1-642		--	
KSR3-Eucidaris-tribuloides/1-545		--	
KSR3-Strongylocentrotus-purpuratus/1-602		--	
KSR3-Paracentrotus-lividus-RNAseq/1-600		--	
KSR3-Notospermus-geniculatus/1-691	20	-RIS--------SLSNHVDGKMDT--------------------------------	34
KSR3-Aplysia-californica/1-405		--	
KSR3-Amphiura-filiformis-partial/1-642	1	-----------------VNGACEA--------------------------------	7
KSR3-Platynereis-dumerilii/1-704	6	-RGA--------SSDPGCDASCDE--------------------------------	20
KSR3-Patiria-miniata/1-779	57	---R--------N---	58
KSR3-Anneissia-japonica/1-590	6	-SEK--------Q---	9
KSR3-Parastichopus-parvimensis-partial/1-330		--	

```
BRAF-Capitella-teleta/1-419                        206 LVPE----NCVVYKMN-----SPHKVPWDTDCG-----------L--LAGLQLSVEL 240
BRAF-Priapulus-caudatus/1-368                      175 LTPE----MCRVCVRG-----TRAWVSWDVDLT-----------E--LESLEIEIEL 209
CRAF-Homo-sapiens/1-648                             91 LQPE----CCAVFRLLHEHKGKKARLDWNTDAA-----------S--LIGEELQVDF 130
ARAF-Homo-sapiens/1-606                             54 LNQD----CCVVYRLIK---GRKTVTAWDTAIA-----------P--LDGEELIVEV 90
RAF-Caenorhabditis-elegans/1-813                   120 ITPQ----LCHVNASS---DPKQESIELSLTMEEI---------ASRLPGNELWVHS 160
Raf-Rhipicephalus-sanguineus/1-803                 173 LTTE----MCVVFKCS-----TRAVVDWNEDVS-----------H--IEDDDIQVEI 207
BRAF-Limulus-caudatus/1-781                        175 LTPH----MCVVYRCN-----PRVRIEWDTDIT-----------S--LEGEEITVEI 209
RAF-Ramazzottius-variornatus/1-885                 261 LVPS----KCRVFKFDLKDTNKRIAVKWTDDVA-----------S--IEGDRIVVEE 300
RAF-Schmidtea-mediterranea/1-737                   159 LDPK----KLKAYLKG-------KSIPWNTDAM-----------RVAATGETISVRY 193
RAF-Amphimedon-queenslandica/1-904                 183 LTPN----TCIVYAES-----PRLLIEWDTDTT-----------Q--LSGKEVFVEY 217
RAF-Clytia-hemispherica/1-792                      210 LTTD----SYRVYNKA-----NRELIAWDSDVS-----------L--LDGAEVIVEM 244
BRAF-Crassostrea-gigas/1-688                       137 LSPE----NCNVYDST-----TKMPISWDIDMA-----------N--LAGKDINVCE 171
RAF-Nematostella-vectensis/1-730                   165 IAIE----TCLIFHCG-----TRDIISWDTHLF-----------E--LEGEEISVEM 199
RAF-Acropora-millepora/1-729                       161 LTPE----ACLICDCY-----SRNIVGWDTDLS-----------SF-VEDVELSVER 196
BRAF-Homo-sapiens/1-766                            190 LIPE----CCAVYRIQ---DGEKKPIGWDTDIS-----------W--LTGEELHVEV 226
BRAF-Danio-rerio/1-777                             200 LIPE----CCAVYRVQ---DGEKKPIGWDTDIS-----------W--LTGEELHVEV 236
BRAF-Eucidaris-tribuloides-partial/1-725           162 LTPE----MCVVYKRE-----PRVLVSWDTDMM-----------Y--LAGEEISVEL 196
BRAF-Strongylocentrotus-purpuratus/1-803           151 LTPE----MCIVYRVN-----PRMALSWDTDMM-----------Y--LAGEEISVEL 185
BRAF-Paracentrotus-lividus-RNAseq/1-746            182 LTPE----MCIVYRTN-----PRMALSWETDMM-----------Y--LAGEEISVEL 216
BRAF-Paracentrotus-lividus-contigEST/1-716         152 LTPE----MCIVYRTN-----PRMALSWETDMM-----------Y--LAGEEISVEL 186
BRAF-Saccoglossus-kowaleskii/1-733                 117 YSSE----TCLVFRKD-----TGGPVDWDTDMS-----------W--LEGEELLVQV 151
RAF-Parastichopus-parvimensis/1-747                137 LTPE----MCVVYKFN---DDKKTQISWDTDMM-----------Y--LSGGEITVEL 173
BRAF-Branchiostoma-floridae/1-831                  169 VSAE----TCRVFRIS-----PRVMVEWDTDMS-----------W--LEGEEISVEI 203
BRAF-Trichoplax-adherens-partial/1-325                 ---------------------------------------------------------
BRAF-Patiria-miniata-partial/1-819                 241 LTVE----MCNVYKRQ-----PRHPVAWDTDMM-----------Y--LAGEE----- 270
KSR1-Caenorhabditis-elegans/1-771                  170 I---------VTPSPKF---NVPSLS---------VTS------------A-KMI-- 190
KSR2-Caenorhabditis-elegans/1-550                   49 S---------VTSSNPI---NAPPPT---------ATS------------SSSVL-- 70
KSR1-Amphimedon-queenslandica/1-1246               485 LPL----IQCSVEQSEF---PSSSVK------SHSVPR---------------LANL 513
KSR1-Patiria-miniata/1-787                         348 ------------MSPH------------------------------------KGMFN 356
KSR1-Anneissia-japonica/1-885                      305 ---GQ-------TSPRK---SS------------------------------P---- 314
KSR1-Parastichopus-parvimensis/1-879               333 RRNSH-------SSPFR---TDPNRL-----------D--------------SPMVL 354
KSR1-Eucidaris-tribuloides/1-873                   305 --RAN-------HSPSR---KI-SPG-----------S--------------VGMLP 323
KSR1-Strongylocentrotus-purpuratus/1-869           305 --LSS---------PCR---SEERLT-----------D--------------IGV-- 320
KSR1-Paracentrotus-lividus/1-848                   299 --LPS---------PSR---SEERLT-----------D--------------IGL-- 314
KSR1-Trichoplax-adherens/1-966                     328 MRTTDKNIQCRLPLQAA---MLSGSC-------EAL----------LHDSQNCTNDI 364
KSR1-Schmidtea-mediterranea/1-954                  375 LKVNDG--NCLTNQQ-----FQPSVFQFNQDNLAVINYGMIEKERRSSYEEKLM--- 421
KSR1-Priapulus-caudatus-partial/1-248               40 GQSGHS--SPSVSSPAR---SPPGG-------------------------------- 59
KSR1-Clytia-hemispherica/1-881                     263 IITI-----------------PPST-----------------------DGETDSDHS 279
KSR1-Nematostella-vectensis-partial/1-917          295 LTI-------------------------------EIPK--------AESGDQLSVLS 312
KSR1-Acropora-millepora/1-957                      354 MTI-------------------------------QIPK--------SVSGDIC---- 367
KSR1-Drosophila-melanogaster/1-966                 312 ----EQ--LARNRLPTD---PSPDSH------SST--------------SSDIFVDP 339
KSR1-Crassostrea-gigas/1-859                       316 LTLENT---HVVRRPSN---EDPYPS------PSQ-------------TSPSCL--- 344
KSR1-Saccoglossus-kowalevskii/1-898                309 ASSGQS--SPRKNSPAR---TPPTPH--------------------------HFIHD 334
KSR1-Branchiostoma-floridae/1-865                  260 LRVIQ----KKMKTPGT---PPPSKS------NLQLPEYQLNPLTRSRSHEAQLTHR 303
KSR1-Capitella-teleta/1-536                            ---------------------------------------------------------
KSR1-Rhipicephalus-sanguineus/1-864                285 LELAN----CSPLSPSR---SPPFVS----------PDQQ---------GDACFV-- 313
KSR1-Limulus-polyphemus/1-603                          ---------------------------------------------------------
KSR3-Ramazzottius-variornatus-partial/1-451            ---------------------------------------------------------
KSR2-Homo-sapiens/1-950                            368 FFVGH---------A------------------PFLPS------T-------PPVHT 384
KSR2-Danio-rerio/1-966                             375 FFFP-----------------------------NFVPS------T-------PPVHS 389
KSR1-Homo-sapiens/1-923                                ---------------------------------------------------------
KSR1-Danio-rerio/1-898                             284 APATA---------P------------------YTLPG------T-------PTLQE 300
KSR3-Clytia-hemispherica/1-517                         ---------------------------------------------------------
KSR3-Schmidtea-mediterranea/1-666                      ---------------------------------------------------------
KSR3-Rhipicephalus-sanguineus/1-675                    ---------------------------------------------------------
KSR3-Limulus-polyphemus/1-686                          ---------------------------------------------------------
KSR3-Limulus-polyphemus-isoformB/1-671                 ---------------------------------------------------------
KSR3-Lingula-anatina/1-626                             ---------------------------------------------------------
KSR3-Crassostrea-gigas/1-585                           ---------------------------------------------------------
KSR3-Nematostella-vectensis/1-640                      ---------------------------------------------------------
KSR3-Acropora-millepora/1-634                          ---------------------------------------------------------
KSR3-Priapulus-caudatus/1-533                          ---------------------------------------------------------
KSR3-Saccoglossus-kowaleskii/1-591                     ---------------------------------------------------------
KSR3-Ptychodera-flava/1-629                            ---------------------------------------------------------
KSR3-Trichoplax-adherens/1-510                         ---------------------------------------------------------
KSR3-Capitella-teleta/1-642                            ---------------------------------------------------------
KSR3-Eucidaris-tribuloides/1-545                       ---------------------------------------------------------
KSR3-Strongylocentrotus-purpuratus/1-602               ---------------------------------------------------------
KSR3-Paracentrotus-lividus-RNAseq/1-600                ---------------------------------------------------------
KSR3-Notospermus-geniculatus/1-691                     ---------------------------------------------------------
KSR3-Aplysia-californica/1-405                         ---------------------------------------------------------
KSR3-Amphiura-filiformis-partial/1-642                 ---------------------------------------------------------
KSR3-Platynereis-dumerilii/1-704                       ---------------------------------------------------------
KSR3-Patiria-miniata/1-779                             ---------------------------------------------------------
KSR3-Anneissia-japonica/1-590                          ---------------------------------------------------------
KSR3-Parastichopus-parvimensis-partial/1-330           ---------------------------------------------------------
```

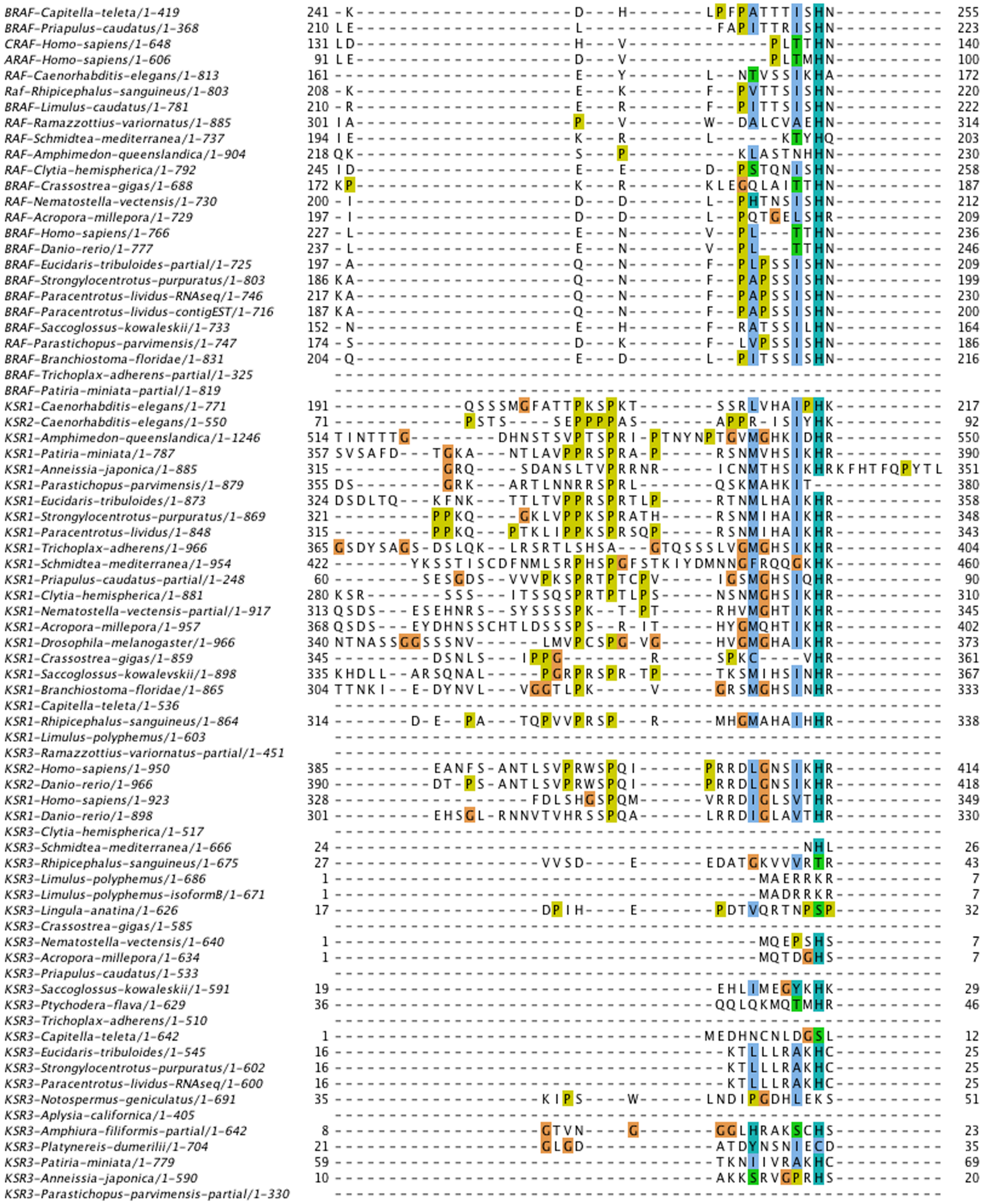
BRAF-Capitella-teleta/1-419 241 255
BRAF-Priapulus-caudatus/1-368 210 223
CRAF-Homo-sapiens/1-648 131 140
ARAF-Homo-sapiens/1-606 91 100
RAF-Caenorhabditis-elegans/1-813 161 172
Raf-Rhipicephalus-sanguineus/1-803 208 220
BRAF-Limulus-caudatus/1-781 210 222
RAF-Ramazzottius-variornatus/1-885 301 314
RAF-Schmidtea-mediterranea/1-737 194 203
RAF-Amphimedon-queenslandica/1-904 218 230
RAF-Clytia-hemispherica/1-792 245 258
BRAF-Crassostrea-gigas/1-688 172 187
RAF-Nematostella-vectensis/1-730 200 212
RAF-Acropora-millepora/1-729 197 209
BRAF-Homo-sapiens/1-766 227 236
BRAF-Danio-rerio/1-777 237 246
BRAF-Eucidaris-tribuloides-partial/1-725 197 209
BRAF-Strongylocentrotus-purpuratus/1-803 186 199
BRAF-Paracentrotus-lividus-RNAseq/1-746 217 230
BRAF-Paracentrotus-lividus-contigEST/1-716 187 200
BRAF-Saccoglossus-kowaleskii/1-733 152 164
RAF-Parastichopus-parvimensis/1-747 174 186
BRAF-Branchiostoma-floridae/1-831 204 216
BRAF-Trichoplax-adherens-partial/1-325
BRAF-Patiria-miniata-partial/1-819
KSR1-Caenorhabditis-elegans/1-771 191 217
KSR2-Caenorhabditis-elegans/1-550 71 92
KSR1-Amphimedon-queenslandica/1-1246 514 550
KSR1-Patiria-miniata/1-787 357 390
KSR1-Anneissia-japonica/1-885 315 351
KSR1-Parastichopus-parvimensis/1-879 355 380
KSR1-Eucidaris-tribuloides/1-873 324 358
KSR1-Strongylocentrotus-purpuratus/1-869 321 348
KSR1-Paracentrotus-lividus/1-848 315 343
KSR1-Trichoplax-adherens/1-966 365 404
KSR1-Schmidtea-mediterranea/1-954 422 460
KSR1-Priapulus-caudatus-partial/1-248 60 90
KSR1-Clytia-hemispherica/1-881 280 310
KSR1-Nematostella-vectensis-partial/1-917 313 345
KSR1-Acropora-millepora/1-957 368 402
KSR1-Drosophila-melanogaster/1-966 340 373
KSR1-Crassostrea-gigas/1-859 345 361
KSR1-Saccoglossus-kowalevskii/1-898 335 367
KSR1-Branchiostoma-floridae/1-865 304 333
KSR1-Capitella-teleta/1-536
KSR1-Rhipicephalus-sanguineus/1-864 314 338
KSR1-Limulus-polyphemus/1-603
KSR3-Ramazzottius-variornatus-partial/1-451
KSR2-Homo-sapiens/1-950 385 414
KSR2-Danio-rerio/1-966 390 418
KSR1-Homo-sapiens/1-923 328 349
KSR1-Danio-rerio/1-898 301 330
KSR3-Clytia-hemispherica/1-517
KSR3-Schmidtea-mediterranea/1-666 24 26
KSR3-Rhipicephalus-sanguineus/1-675 27 43
KSR3-Limulus-polyphemus/1-686 1 7
KSR3-Limulus-polyphemus-isoformB/1-671 1 7
KSR3-Lingula-anatina/1-626 17 32
KSR3-Crassostrea-gigas/1-585
KSR3-Nematostella-vectensis/1-640 1 7
KSR3-Acropora-millepora/1-634 1 7
KSR3-Priapulus-caudatus/1-533
KSR3-Saccoglossus-kowaleskii/1-591 19 29
KSR3-Ptychodera-flava/1-629 36 46
KSR3-Trichoplax-adherens/1-510
KSR3-Capitella-teleta/1-642 1 12
KSR3-Eucidaris-tribuloides/1-545 16 25
KSR3-Strongylocentrotus-purpuratus/1-602 16 25
KSR3-Paracentrotus-lividus-RNAseq/1-600 16 25
KSR3-Notospermus-geniculatus/1-691 35 51
KSR3-Aplysia-californica/1-405
KSR3-Amphiura-filiformis-partial/1-642 8 23
KSR3-Platynereis-dumerilii/1-704 21 35
KSR3-Patiria-miniata/1-779 59 69
KSR3-Anneissia-japonica/1-590 10 20
KSR3-Parastichopus-parvimensis-partial/1-330

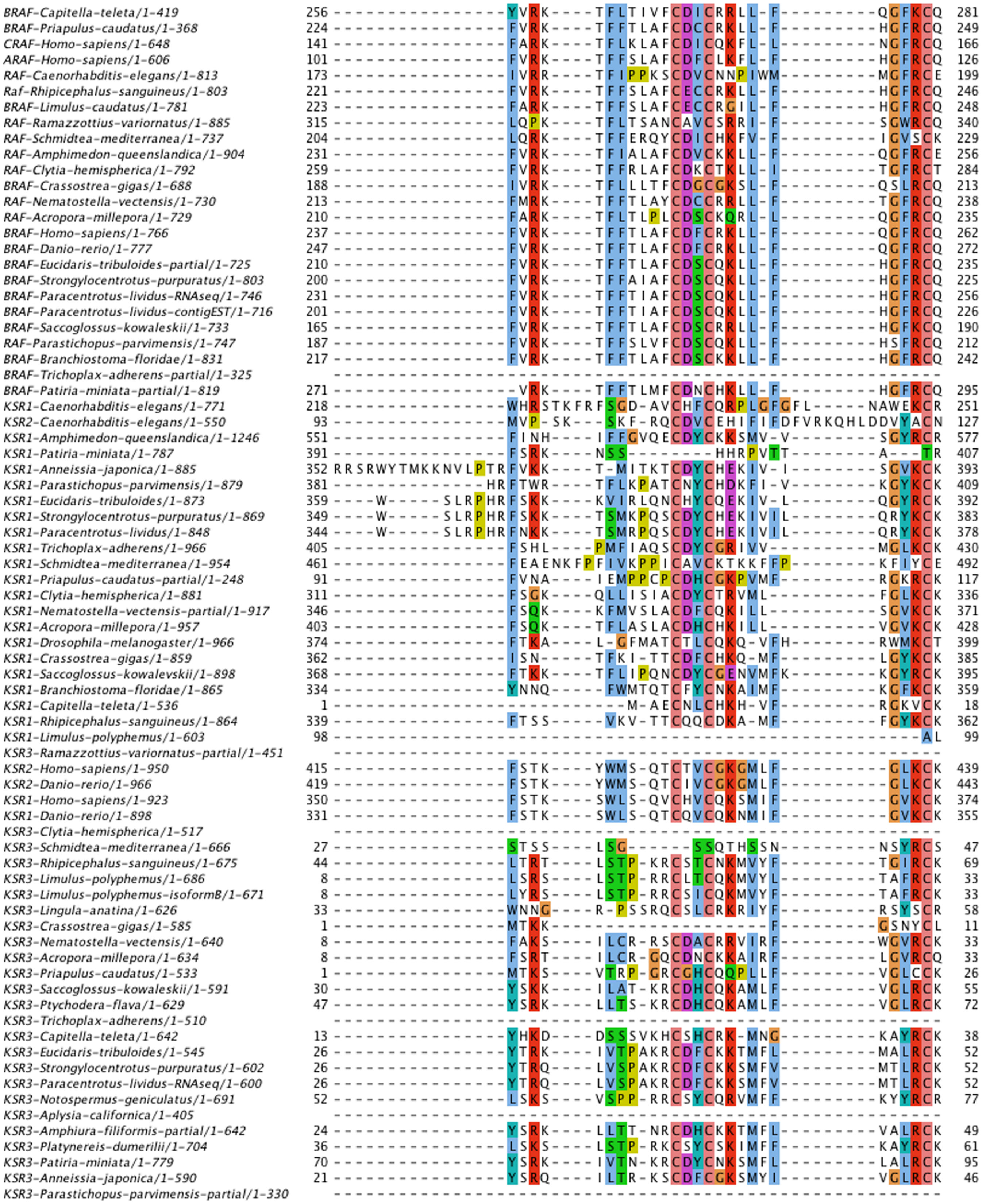
BRAF-Capitella-teleta/1-419
BRAF-Priapulus-caudatus/1-368
CRAF-Homo-sapiens/1-648
ARAF-Homo-sapiens/1-606
RAF-Caenorhabditis-elegans/1-813
Raf-Rhipicephalus-sanguineus/1-803
BRAF-Limulus-caudatus/1-781
RAF-Ramazzottius-variornatus/1-885
RAF-Schmidtea-mediterranea/1-737
RAF-Amphimedon-queenslandica/1-904
RAF-Clytia-hemispherica/1-792
BRAF-Crassostrea-gigas/1-688
RAF-Nematostella-vectensis/1-730
RAF-Acropora-millepora/1-729
BRAF-Homo-sapiens/1-766
BRAF-Danio-rerio/1-777
BRAF-Eucidaris-tribuloides-partial/1-725
BRAF-Strongylocentrotus-purpuratus/1-803
BRAF-Paracentrotus-lividus-RNAseq/1-746
BRAF-Paracentrotus-lividus-contigEST/1-716
BRAF-Saccoglossus-kowaleskii/1-733
RAF-Parastichopus-parvimensis/1-747
BRAF-Branchiostoma-floridae/1-831
BRAF-Trichoplax-adherens-partial/1-325
BRAF-Patiria-miniata-partial/1-819
KSR1-Caenorhabditis-elegans/1-771
KSR2-Caenorhabditis-elegans/1-550
KSR1-Amphimedon-queenslandica/1-1246
KSR1-Patiria-miniata/1-787
KSR1-Anneissia-japonica/1-885
KSR1-Parastichopus-parvimensis/1-879
KSR1-Eucidaris-tribuloides/1-873
KSR1-Strongylocentrotus-purpuratus/1-869
KSR1-Paracentrotus-lividus/1-848
KSR1-Trichoplax-adherens/1-966
KSR1-Schmidtea-mediterranea/1-954
KSR1-Priapulus-caudatus-partial/1-248
KSR1-Clytia-hemispherica/1-881
KSR1-Nematostella-vectensis-partial/1-917
KSR1-Acropora-millepora/1-957
KSR1-Drosophila-melanogaster/1-966
KSR1-Crassostrea-gigas/1-859
KSR1-Saccoglossus-kowalevskii/1-898
KSR1-Branchiostoma-floridae/1-865
KSR1-Capitella-teleta/1-536
KSR1-Rhipicephalus-sanguineus/1-864
KSR1-Limulus-polyphemus/1-603
KSR3-Ramazzottius-variornatus-partial/1-451
KSR2-Homo-sapiens/1-950
KSR2-Danio-rerio/1-966
KSR1-Homo-sapiens/1-923
KSR1-Danio-rerio/1-898
KSR3-Clytia-hemispherica/1-517
KSR3-Schmidtea-mediterranea/1-666
KSR3-Rhipicephalus-sanguineus/1-675
KSR3-Limulus-polyphemus/1-686
KSR3-Limulus-polyphemus-isoformB/1-671
KSR3-Lingula-anatina/1-626
KSR3-Crassostrea-gigas/1-585
KSR3-Nematostella-vectensis/1-640
KSR3-Acropora-millepora/1-634
KSR3-Priapulus-caudatus/1-533
KSR3-Saccoglossus-kowaleskii/1-591
KSR3-Ptychodera-flava/1-629
KSR3-Trichoplax-adherens/1-510
KSR3-Capitella-teleta/1-642
KSR3-Eucidaris-tribuloides/1-545
KSR3-Strongylocentrotus-purpuratus/1-602
KSR3-Paracentrotus-lividus-RNAseq/1-600
KSR3-Notospermus-geniculatus/1-691
KSR3-Aplysia-californica/1-405
KSR3-Amphiura-filiformis-partial/1-642
KSR3-Platynereis-dumerilii/1-704
KSR3-Patiria-miniata/1-779
KSR3-Anneissia-japonica/1-590
KSR3-Parastichopus-parvimensis-partial/1-330

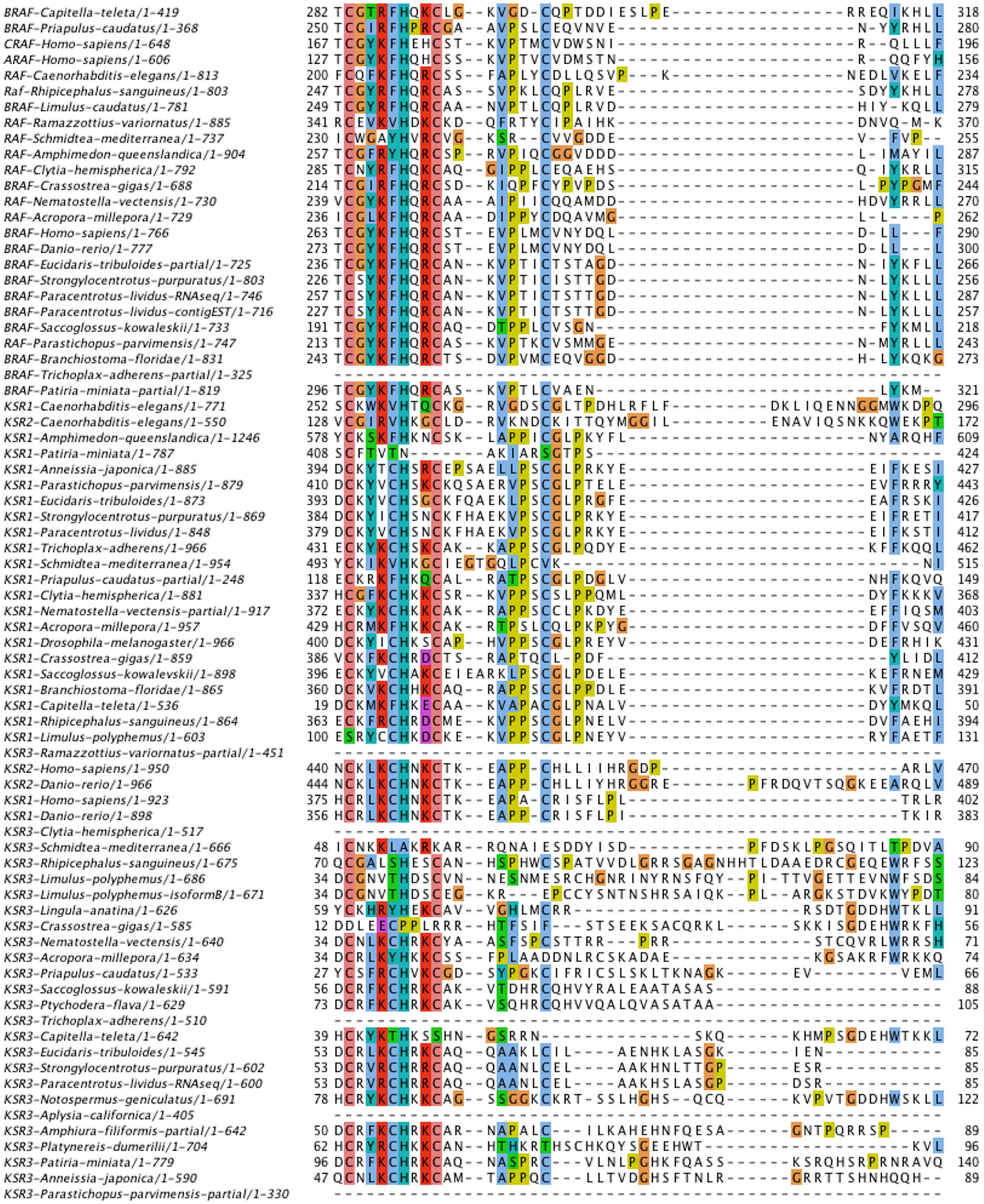
BRAF-Capitella-teleta/1-419
BRAF-Priapulus-caudatus/1-368
CRAF-Homo-sapiens/1-648
ARAF-Homo-sapiens/1-606
RAF-Caenorhabditis-elegans/1-813
Raf-Rhipicephalus-sanguineus/1-803
BRAF-Limulus-caudatus/1-781
RAF-Ramazzottius-variornatus/1-885
RAF-Schmidtea-mediterranea/1-737
RAF-Amphimedon-queenslandica/1-904
RAF-Clytia-hemispherica/1-792
BRAF-Crassostrea-gigas/1-688
RAF-Nematostella-vectensis/1-730
RAF-Acropora-millepora/1-729
BRAF-Homo-sapiens/1-766
BRAF-Danio-rerio/1-777
BRAF-Eucidaris-tribuloides-partial/1-725
BRAF-Strongylocentrotus-purpuratus/1-803
BRAF-Paracentrotus-lividus-RNAseq/1-746
BRAF-Paracentrotus-lividus-contigEST/1-716
BRAF-Saccoglossus-kowaleskii/1-733
RAF-Parastichopus-parvimensis/1-747
BRAF-Branchiostoma-floridae/1-831
BRAF-Trichoplax-adherens-partial/1-325
BRAF-Patiria-miniata-partial/1-819
KSR1-Caenorhabditis-elegans/1-771
KSR2-Caenorhabditis-elegans/1-550
KSR1-Amphimedon-queenslandica/1-1246
KSR1-Patiria-miniata/1-787
KSR1-Anneissia-japonica/1-885
KSR1-Parastichopus-parvimensis/1-879
KSR1-Eucidaris-tribuloides/1-873
KSR1-Strongylocentrotus-purpuratus/1-869
KSR1-Paracentrotus-lividus/1-848
KSR1-Trichoplax-adherens/1-966
KSR1-Schmidtea-mediterranea/1-954
KSR1-Priapulus-caudatus-partial/1-248
KSR1-Clytia-hemispherica/1-881
KSR1-Nematostella-vectensis-partial/1-917
KSR1-Acropora-millepora/1-957
KSR1-Drosophila-melanogaster/1-966
KSR1-Crassostrea-gigas/1-859
KSR1-Saccoglossus-kowalevskii/1-898
KSR1-Branchiostoma-floridae/1-865
KSR1-Capitella-teleta/1-536
KSR1-Rhipicephalus-sanguineus/1-864
KSR1-Limulus-polyphemus/1-603
KSR3-Ramazzottius-variornatus-partial/1-451
KSR2-Homo-sapiens/1-950
KSR2-Danio-rerio/1-966
KSR1-Homo-sapiens/1-923
KSR1-Danio-rerio/1-898
KSR3-Clytia-hemispherica/1-517
KSR3-Schmidtea-mediterranea/1-666
KSR3-Rhipicephalus-sanguineus/1-675
KSR3-Limulus-polyphemus/1-686
KSR3-Limulus-polyphemus-isoformB/1-671
KSR3-Lingula-anatina/1-626
KSR3-Crassostrea-gigas/1-585
KSR3-Nematostella-vectensis/1-640
KSR3-Acropora-millepora/1-634
KSR3-Priapulus-caudatus/1-533
KSR3-Saccoglossus-kowaleskii/1-591
KSR3-Ptychodera-flava/1-629
KSR3-Trichoplax-adherens/1-510
KSR3-Capitella-teleta/1-642
KSR3-Eucidaris-tribuloides/1-545
KSR3-Strongylocentrotus-purpuratus/1-602
KSR3-Paracentrotus-lividus-RNAseq/1-600
KSR3-Notospermus-geniculatus/1-691
KSR3-Aplysia-californica/1-405
KSR3-Amphiura-filiformis-partial/1-642
KSR3-Platynereis-dumerilii/1-704
KSR3-Patiria-miniata/1-779
KSR3-Anneissia-japonica/1-590
KSR3-Parastichopus-parvimensis-partial/1-330

```
BRAF-Capitella-teleta/1-419                        319 AENVSAAG---------LLSPPTSHPR----------------------------G 337
BRAF-Priapulus-caudatus/1-368                      281 AHNDKALGMGA------------------------LASPSNVARSPSPSPVRRSAP 312
CRAF-Homo-sapiens/1-648                            197 PNSTIGDSG-------------------------VPALPSL-TMRRMRESVSRMPV 226
ARAF-Homo-sapiens/1-606                            157 SVQDLSGGS------------------------RQHEAP---SNRPLNELLTPQGP 185
RAF-Caenorhabditis-elegans/1-813                   235 GIASQVE-GPDRSVAEIVLAN--LAPTS----GQSPAATPD-SSHPDLTSIKRTGG 282
Raf-Rhipicephalus-sanguineus/1-803                 279 AMPDMVGG-PQYE-SGV------YPPFMPP--------PGG-GGEPVLSPT----- 312
BRAF-Limulus-caudatus/1-781                        280 AMNNTASNMLQTT-GDS------YSPYASN--------YQY-QRQP--AVT----- 312
RAF-Ramazzottius-variornatus/1-885                 371 AKMFL-AN--DAN-GEA------FLPYGL--------------------------- 389
RAF-Schmidtea-mediterranea/1-737                   256 -------------------------NYGGESSAGYSNKP-------NTS------- 272
RAF-Amphimedon-queenslandica/1-904                 288 SQHPP---KPD----D--------SPQ--------PSSP--------------VPH 306
RAF-Clytia-hemispherica/1-792                      316 AKQNP-----N--------------------------------------------- 321
BRAF-Crassostrea-gigas/1-688                       245 KPKL---------------------------------------------------- 248
RAF-Nematostella-vectensis/1-730                   271 AGRNEF-------------------------------------------------- 276
RAF-Acropora-millepora/1-729                       263 GLG------PD----TLFVEE-----------------------------L----- 274
BRAF-Homo-sapiens/1-766                            291 VSKFF-EHHPIPQ-EEASLAET------ALTSGSSPSAPASDSIGPQILTS--PSP 336
BRAF-Danio-rerio/1-777                             301 ASKFL-VHHPITQ-EEVSSEGT------TPISEMCPSLPPSESTGSLCHPT--VSP 346
BRAF-Eucidaris-tribuloides-partial/1-725           267 ALRRDDE-----------------GPYPDP----------------FPEPD---SP 286
BRAF-Strongylocentrotus-purpuratus/1-803           257 ALRKEDDY---------------NDYPDT-----------------FP--D---EP 275
BRAF-Paracentrotus-lividus-RNAseq/1-746            288 ALRKEDDY-----------------DYPDP----------------FP--D---EP 305
BRAF-Paracentrotus-lividus-contigEST/1-716         258 ALRKEDDY-----------------DYPDP----------------FP--D---EP 275
BRAF-Saccoglossus-kowaleskii/1-733                 219 GLKGTGAEMPSDS-RSVFLDSDTEQ-----------NDPGY--GKGMTLPL---G- 256
RAF-Parastichopus-parvimensis/1-747                244 GMA----EGKD----D-SRKNAEDGPYGGEMDEAEAEN-----ESPVTTPT---SS 282
BRAF-Branchiostoma-floridae/1-831                  274 PIQSLLAQNSN----DVFIQPTTSQPGGS-MQGGSLRTPSTGQSYLSTQPI---PT 321
BRAF-Trichoplax-adherens-partial/1-325                 --------------------------------------------------------
BRAF-Patiria-miniata-partial/1-819                 322 ----LLMKNVD----E-D-----EDPYG-----EEPES------------------ 340
KSR1-Caenorhabditis-elegans/1-771                  297 SV------------------------------------------------------ 298
KSR2-Caenorhabditis-elegans/1-550                  173 TA------------------------------------------------------ 174
KSR1-Amphimedon-queenslandica/1-1246               610 MMSPSH-----------------ESVYQVNKTGHLTSSGEY----G-----S---- 635
KSR1-Patiria-miniata/1-787                         425 -----------------------------P----------L----L-----I---- 428
KSR1-Anneissia-japonica/1-885                      428 THQGYA--------------------SPSP----------I----F-----H---- 440
KSR1-Parastichopus-parvimensis/1-879               444 ENPVPN-------------------------------------------------- 449
KSR1-Eucidaris-tribuloides/1-873                   427 EAVNID--------------------NS------------L----G-----R---- 437
KSR1-Strongylocentrotus-purpuratus/1-869           418 NSGRTA--------------------SP------------I----M-----S---- 428
KSR1-Paracentrotus-lividus/1-848                   413 EQARTE--------------------KP---------------------------- 420
KSR1-Trichoplax-adherens/1-966                     463 LHDNLN--------------------LSQD----------T----S-----K---- 475
KSR1-Schmidtea-mediterranea/1-954                  516 --------------------------KSPP----------A----D-----R---- 522
KSR1-Priapulus-caudatus-partial/1-248              150 FSFSGG-----------------EENPSPV----------A----A-----A---- 165
KSR1-Clytia-hemispherica/1-881                     369 DNESRA--------------------QVDD----------R----K-----K---- 381
KSR1-Nematostella-vectensis-partial/1-917          404 RRGYEY--------------------RSLP----------I----I-----K---- 416
KSR1-Acropora-millepora/1-957                      461 RRAWEY--------------------RSLP----------S----T-----R---- 473
KSR1-Drosophila-melanogaster/1-966                 432 EQ-GGY--------------------ASLP----------H----V-----H---- 443
KSR1-Crassostrea-gigas/1-859                       413 KHRDGS--------------------PSPS----------A----E-----R---- 425
KSR1-Saccoglossus-kowalevskii/1-898                430 TN-PAM--------------------RQAA----------A----E-----A---- 441
KSR1-Branchiostoma-floridae/1-865                  392 QG-TAQ--------------------WGQN----------W----G-----S---- 403
KSR1-Capitella-teleta/1-536                         51 RS-GNE---------------------SPN----------I----L-----R----  61
KSR1-Rhipicephalus-sanguineus/1-864                395 QK-GGM--------------------QSPN----------H----A-----A---- 406
KSR1-Limulus-polyphemus/1-603                      132 KN-EGN--------------------YSPI----------L----N-----Q---- 143
KSR3-Ramazzottius-variornatus-partial/1-451            --------------------------------------------------------
KSR2-Homo-sapiens/1-950                            471 RTES------------------------------VPCDINN----P----LRK--- 485
KSR2-Danio-rerio/1-966                             490 RTES------------------------------VPCDINN----P----L----- 502
KSR1-Homo-sapiens/1-923                            403 RTES------------------------------VPSDINN----P----VDR--- 417
KSR1-Danio-rerio/1-898                             384 RTES------------------------------VPSDINN----P----VDR--- 398
KSR3-Clytia-hemispherica/1-517                         --------------------------------------------------------
KSR3-Schmidtea-mediterranea/1-666                   91 LMND-K----------------------PPLSFLDPSSKNP----L----SSTSS- 114
KSR3-Rhipicephalus-sanguineus/1-675                124 VGHE-V-----------------LTVSPLMDQPFLFSSGDH----G-----TLASA 152
KSR3-Limulus-polyphemus/1-686                       85 EKSA-F-----------------SSPTATENN-------------L-----PRSSF 104
KSR3-Limulus-polyphemus-isoformB/1-671              81 EKTD-V-----------------ISLDTTESN-------------E-----PRMSA 100
KSR3-Lingula-anatina/1-626                          92 QANE-I-----------------NSNMDLSVLQSL----NN----L---------- 111
KSR3-Crassostrea-gigas/1-585                        57 TTNE-T-----------------PVIFHSRPHRPL--IRRS---------LKR---  80
KSR3-Nematostella-vectensis/1-640                   72 FGKD-V-----------------KRPS-DTGRGLA-----------------QMRE  91
KSR3-Acropora-millepora/1-634                       75 NARE-K-----------------ISIHQANVEHLV-----------------RKRN  95
KSR3-Priapulus-caudatus/1-533                       67 QTHE-V-----------------NRNKLPSI---------------------Y---  80
KSR3-Saccoglossus-kowaleskii/1-591                  89 ------------------------QRLRPCRALLVEDKQFV----A----EDR--- 109
KSR3-Ptychodera-flava/1-629                        106 ---R-A-----------------SQRLRQWVPSVERRKRNS----A-----KA--- 128
KSR3-Trichoplax-adherens/1-510                         --------------------------------------------------------
KSR3-Capitella-teleta/1-642                         73 QTTP-D-----------------DQDLCSAKHALQ--HQES----Y----CK----  96
KSR3-Eucidaris-tribuloides/1-545                    86 ------------------------QTAQRNLDIQT--SHDA----S----GKP--- 104
KSR3-Strongylocentrotus-purpuratus/1-602            86 ------------------------NVVPTCHAALAEASRRH----Q----SAN--- 106
KSR3-Paracentrotus-lividus-RNAseq/1-600             86 ------------------------NSVPSCHTALE-ASRRQ----Q----SVN--- 105
KSR3-Notospermus-geniculatus/1-691                 123 QANE-V-----------------NSNV-----VVVPDDGRH----Y----CHH--- 144
KSR3-Aplysia-californica/1-405                         --------------------------------------------------------
KSR3-Amphiura-filiformis-partial/1-642              90 -------------------------TQRSQGEFLTPQHHRH----H----HQRSGP 112
KSR3-Platynereis-dumerilii/1-704                    97 QANE-V-----------------NSNL-----LLVPCHGRR----G----HSYSEK 121
KSR3-Patiria-miniata/1-779                         141 LAHQ-A-----------------EPSYEPMSTSSTPQHHRE----G----LSR--- 167
KSR3-Anneissia-japonica/1-590                       90 ---E-Q-----------------PQTFCTCVQKPRPILHRQ----H----SVT--- 113
KSR3-Parastichopus-parvimensis-partial/1-330           --------------------------------------------------------
```

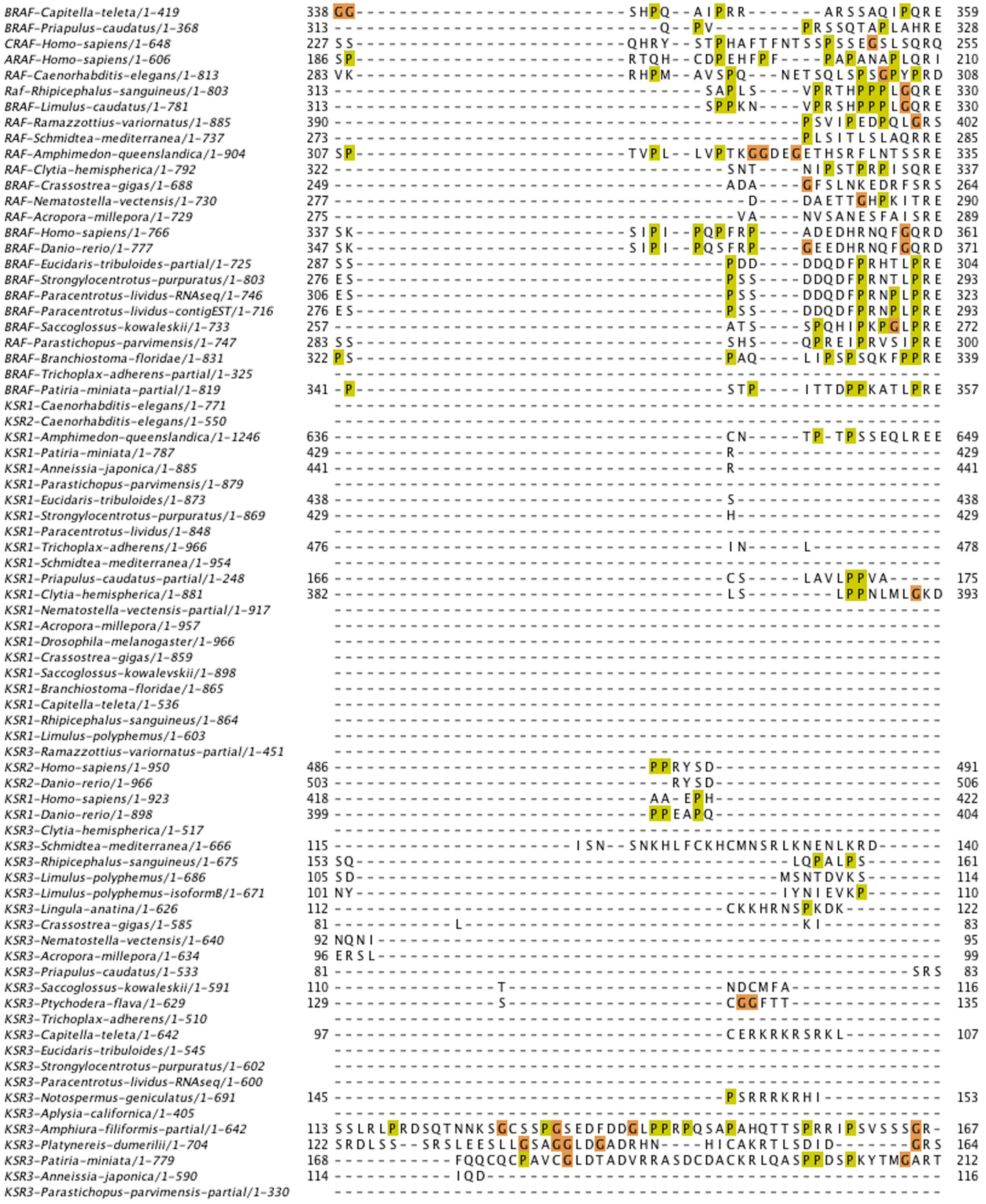
BRAF-Capitella-teleta/1-419 338 GG - SHPQ - AIPRR - ARSSAQIPQRE 359
BRAF-Priapulus-caudatus/1-368 313 - Q - PV - PRSSQTAPLAHRE 328
CRAF-Homo-sapiens/1-648 227 SS - QHRY - STPHAFTFNTSSPSSEGSLSQRQ 255
ARAF-Homo-sapiens/1-606 186 SP - RTQH - CDPEHFPF - PAPANAPLQRI 210
RAF-Caenorhabditis-elegans/1-813 283 VK - RHPM - AVSPQ - NETSQLSPSGPYPRD 308
Raf-Rhipicephalus-sanguineus/1-803 313 - SAPLS - VPRTHPPPLGQRE 330
BRAF-Limulus-caudatus/1-781 313 - SPPKN - VPRSHPPPLGQRE 330
RAF-Ramazzottius-variornatus/1-885 390 - PSVIPEDPQLGRS 402
RAF-Schmidtea-mediterranea/1-737 273 - PLSITLSLAQRRE 285
RAF-Amphimedon-queenslandica/1-904 307 SP - TVPL - LVPTKGGDEGETHSRFLNTSSRE 335
RAF-Clytia-hemispherica/1-792 322 - SNT - NIPSTPRPISQRE 337
BRAF-Crassostrea-gigas/1-688 249 - ADA - GFSLNKEDRFSRS 264
RAF-Nematostella-vectensis/1-730 277 - D - DAETTGHPKITRE 290
RAF-Acropora-millepora/1-729 275 - VA - NVSANESFAISRE 289
BRAF-Homo-sapiens/1-766 337 SK - SIPI - PQPFRP - ADEDHRNQFGQRD 361
BRAF-Danio-rerio/1-777 347 SK - SIPI - PQSFRP - GEEDHRNQFGQRD 371
BRAF-Eucidaris-tribuloides-partial/1-725 287 SS - PDD - DDQDFPRHTLPRE 304
BRAF-Strongylocentrotus-purpuratus/1-803 276 ES - PSS - DDQDFPRNTLPRE 293
BRAF-Paracentrotus-lividus-RNAseq/1-746 306 ES - PSS - DDQDFPRNPLPRE 323
BRAF-Paracentrotus-lividus-contigEST/1-716 276 ES - PSS - DDQDFPRNPLPRE 293
BRAF-Saccoglossus-kowaleskii/1-733 257 - ATS - SPQHIPKPGLPRE 272
RAF-Parastichopus-parvimensis/1-747 283 SS - SHS - QPREIPRVSIPRE 300
BRAF-Branchiostoma-floridae/1-831 322 PS - PAQ - LIPSPSQKFPPRE 339
BRAF-Trichoplax-adherens-partial/1-325
BRAF-Patiria-miniata-partial/1-819 341 - P - STP - ITTDPPKATLPRE 357
KSR1-Caenorhabditis-elegans/1-771
KSR2-Caenorhabditis-elegans/1-550
KSR1-Amphimedon-queenslandica/1-1246 636 - CN - TP - TPSSEQLREE 649
KSR1-Patiria-miniata/1-787 429 - R - 429
KSR1-Anneissia-japonica/1-885 441 - R - 441
KSR1-Parastichopus-parvimensis/1-879
KSR1-Eucidaris-tribuloides/1-873 438 - S - 438
KSR1-Strongylocentrotus-purpuratus/1-869 429 - H - 429
KSR1-Paracentrotus-lividus/1-848
KSR1-Trichoplax-adherens/1-966 476 - IN - L - 478
KSR1-Schmidtea-mediterranea/1-954
KSR1-Priapulus-caudatus-partial/1-248 166 - CS - LAVLPPVA - 175
KSR1-Clytia-hemispherica/1-881 382 - LS - LPPNLMLGKD 393
KSR1-Nematostella-vectensis-partial/1-917
KSR1-Acropora-millepora/1-957
KSR1-Drosophila-melanogaster/1-966
KSR1-Crassostrea-gigas/1-859
KSR1-Saccoglossus-kowalevskii/1-898
KSR1-Branchiostoma-floridae/1-865
KSR1-Capitella-teleta/1-536
KSR1-Rhipicephalus-sanguineus/1-864
KSR1-Limulus-polyphemus/1-603
KSR3-Ramazzottius-variornatus-partial/1-451
KSR2-Homo-sapiens/1-950 486 - PPRYSD - 491
KSR2-Danio-rerio/1-966 503 - RYSD - 506
KSR1-Homo-sapiens/1-923 418 - AA - EPH - 422
KSR1-Danio-rerio/1-898 399 - PPEAPQ - 404
KSR3-Clytia-hemispherica/1-517
KSR3-Schmidtea-mediterranea/1-666 115 - ISN - SNKHLFCKHCMNSRLKNENLKRD - 140
KSR3-Rhipicephalus-sanguineus/1-675 153 SQ - LQPALPS - 161
KSR3-Limulus-polyphemus/1-686 105 SD - MSNTDVKS - 114
KSR3-Limulus-polyphemus-isoformB/1-671 101 NY - IYNIEVKP - 110
KSR3-Lingula-anatina/1-626 112 - CKKHRNSPKDK - 122
KSR3-Crassostrea-gigas/1-585 81 - L - KI - 83
KSR3-Nematostella-vectensis/1-640 92 NQNI - 95
KSR3-Acropora-millepora/1-634 96 ERSL - 99
KSR3-Priapulus-caudatus/1-533 81 - SRS 83
KSR3-Saccoglossus-kowaleskii/1-591 110 - T - NDCMFA - 116
KSR3-Ptychodera-flava/1-629 129 - S - CGGFTT - 135
KSR3-Trichoplax-adherens/1-510
KSR3-Capitella-teleta/1-642 97 - CERKRKRSRKL - 107
KSR3-Eucidaris-tribuloides/1-545
KSR3-Strongylocentrotus-purpuratus/1-602
KSR3-Paracentrotus-lividus-RNAseq/1-600
KSR3-Notospermus-geniculatus/1-691 145 - PSRRRKRHI - 153
KSR3-Aplysia-californica/1-405
KSR3-Amphiura-filiformis-partial/1-642 113 SSLRLPRDSQTNNKSGCSSPGSEDFDDGLPPRPQSAPAHQTTSPRRIPSVSSSGR - 167
KSR3-Platynereis-dumerilii/1-704 122 SRDLSS - SRSLEESLLGSAGGLDGADRHN - HICAKRTLSDID - GRS 164
KSR3-Patiria-miniata/1-779 168 - FQQCQCPAVCGLDTADVRRASDCDACKRLQASPPDSPKYTMGART 212
KSR3-Anneissia-japonica/1-590 114 - IQD - 116
KSR3-Parastichopus-parvimensis-partial/1-330

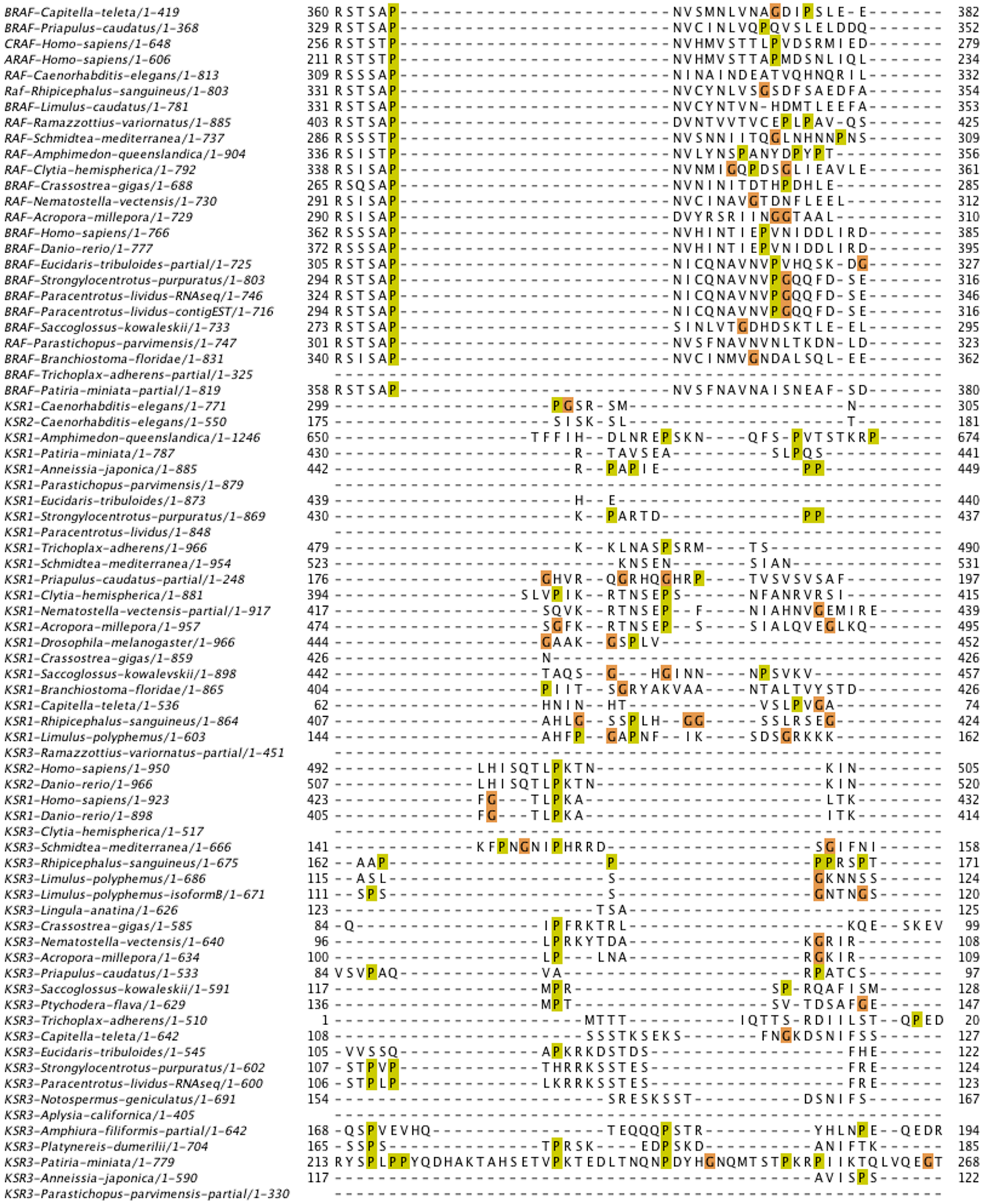
BRAF-Capitella-teleta/1-419
BRAF-Priapulus-caudatus/1-368
CRAF-Homo-sapiens/1-648
ARAF-Homo-sapiens/1-606
RAF-Caenorhabditis-elegans/1-813
Raf-Rhipicephalus-sanguineus/1-803
BRAF-Limulus-caudatus/1-781
RAF-Ramazzottius-variornatus/1-885
RAF-Schmidtea-mediterranea/1-737
RAF-Amphimedon-queenslandica/1-904
RAF-Clytia-hemispherica/1-792
BRAF-Crassostrea-gigas/1-688
RAF-Nematostella-vectensis/1-730
RAF-Acropora-millepora/1-729
BRAF-Homo-sapiens/1-766
BRAF-Danio-rerio/1-777
BRAF-Eucidaris-tribuloides-partial/1-725
BRAF-Strongylocentrotus-purpuratus/1-803
BRAF-Paracentrotus-lividus-RNAseq/1-746
BRAF-Paracentrotus-lividus-contigEST/1-716
BRAF-Saccoglossus-kowaleskii/1-733
RAF-Parastichopus-parvimensis/1-747
BRAF-Branchiostoma-floridae/1-831
BRAF-Trichoplax-adherens-partial/1-325
BRAF-Patiria-miniata-partial/1-819
KSR1-Caenorhabditis-elegans/1-771
KSR2-Caenorhabditis-elegans/1-550
KSR1-Amphimedon-queenslandica/1-1246
KSR1-Patiria-miniata/1-787
KSR1-Anneissia-japonica/1-885
KSR1-Parastichopus-parvimensis/1-879
KSR1-Eucidaris-tribuloides/1-873
KSR1-Strongylocentrotus-purpuratus/1-869
KSR1-Paracentrotus-lividus/1-848
KSR1-Trichoplax-adherens/1-966
KSR1-Schmidtea-mediterranea/1-954
KSR1-Priapulus-caudatus-partial/1-248
KSR1-Clytia-hemispherica/1-881
KSR1-Nematostella-vectensis-partial/1-917
KSR1-Acropora-millepora/1-957
KSR1-Drosophila-melanogaster/1-966
KSR1-Crassostrea-gigas/1-859
KSR1-Saccoglossus-kowalevskii/1-898
KSR1-Branchiostoma-floridae/1-865
KSR1-Capitella-teleta/1-536
KSR1-Rhipicephalus-sanguineus/1-864
KSR1-Limulus-polyphemus/1-603
KSR3-Ramazzottius-variornatus-partial/1-451
KSR2-Homo-sapiens/1-950
KSR2-Danio-rerio/1-966
KSR1-Homo-sapiens/1-923
KSR1-Danio-rerio/1-898
KSR3-Clytia-hemispherica/1-517
KSR3-Schmidtea-mediterranea/1-666
KSR3-Rhipicephalus-sanguineus/1-675
KSR3-Limulus-polyphemus/1-686
KSR3-Limulus-polyphemus-isoformB/1-671
KSR3-Lingula-anatina/1-626
KSR3-Crassostrea-gigas/1-585
KSR3-Nematostella-vectensis/1-640
KSR3-Acropora-millepora/1-634
KSR3-Priapulus-caudatus/1-533
KSR3-Saccoglossus-kowaleskii/1-591
KSR3-Ptychodera-flava/1-629
KSR3-Trichoplax-adherens/1-510
KSR3-Capitella-teleta/1-642
KSR3-Eucidaris-tribuloides/1-545
KSR3-Strongylocentrotus-purpuratus/1-602
KSR3-Paracentrotus-lividus-RNAseq/1-600
KSR3-Notospermus-geniculatus/1-691
KSR3-Aplysia-californica/1-405
KSR3-Amphiura-filiformis-partial/1-642
KSR3-Platynereis-dumerilii/1-704
KSR3-Patiria-miniata/1-779
KSR3-Anneissia-japonica/1-590
KSR3-Parastichopus-parvimensis-partial/1-330

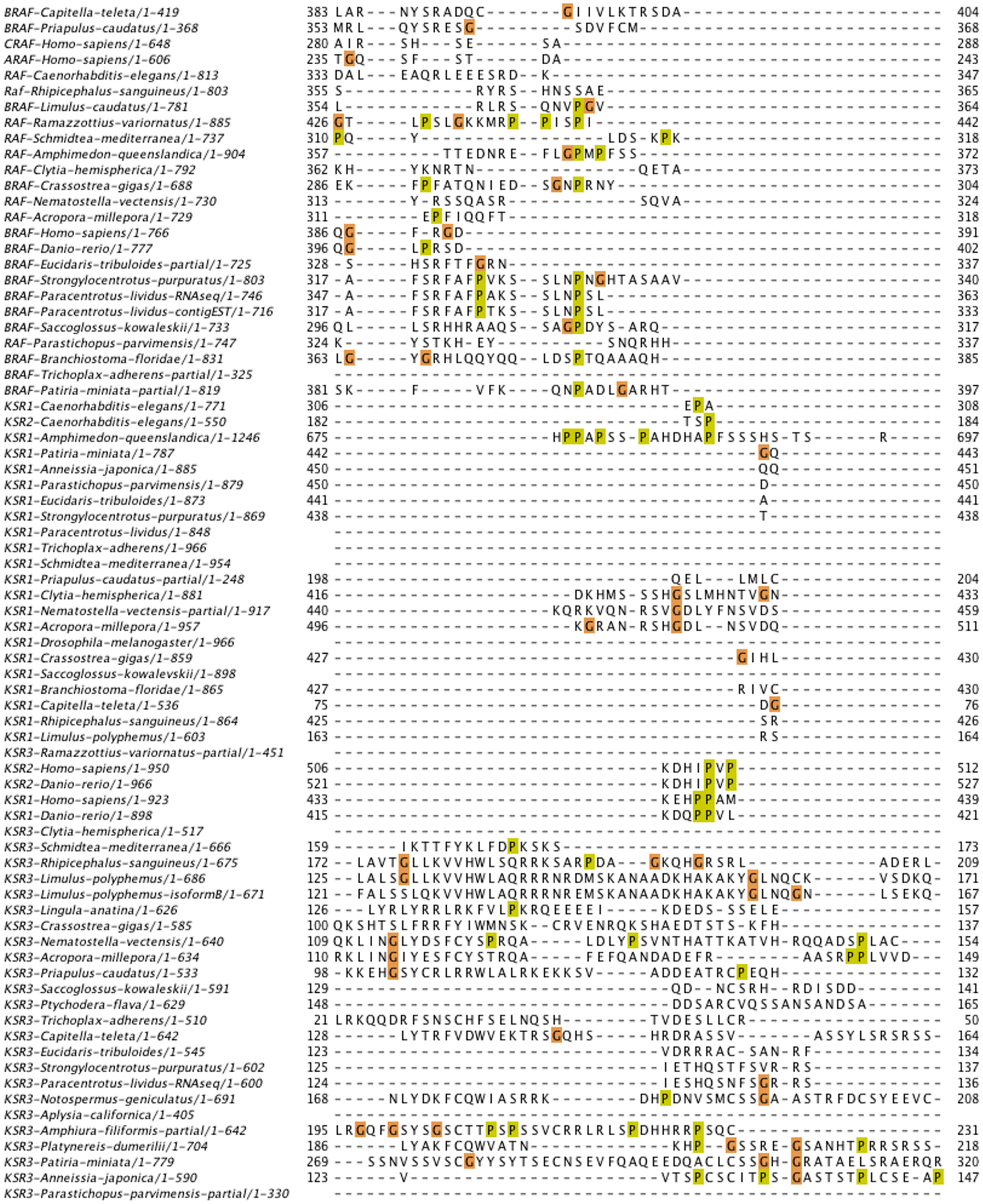

BRAF-Capitella-teleta/1-419 383 LAR---NYSRADQC-------GIIVLKTRSDA 404
BRAF-Priapulus-caudatus/1-368 353 MRL---QYSRESG---------SDVFCM 368
CRAF-Homo-sapiens/1-648 280 AIR---SH---SE------SA 288
ARAF-Homo-sapiens/1-606 235 TGQ---SF---ST------DA 243
RAF-Caenorhabditis-elegans/1-813 333 DAL---EAQRLEEESRD--K 347
Raf-Rhipicephalus-sanguineus/1-803 355 S-----------RYRS--HNSSAE 365
BRAF-Limulus-caudatus/1-781 354 L-----------RLRS--QNVPGV 364
RAF-Ramazzottius-variornatus/1-885 426 GT----LPSLGKKMRP--PISPI 442
RAF-Schmidtea-mediterranea/1-737 310 PQ----Y-----------------LDS-KPK 318
RAF-Amphimedon-queenslandica/1-904 357 ----------TTEDNRE--FLGPMPFSS 372
RAF-Clytia-hemispherica/1-792 362 KH-----YKNRTN-------------QETA 373
BRAF-Crassostrea-gigas/1-688 286 EK-----FPFATQNIED--SGNPRNY 304
RAF-Nematostella-vectensis/1-730 313 -------Y-RSSQASR-----------SQVA 324
RAF-Acropora-millepora/1-729 311 --------EPFIQQFT 318
BRAF-Homo-sapiens/1-766 386 QG-----F-RGD 391
BRAF-Danio-rerio/1-777 396 QG-----LPRSD 402
BRAF-Eucidaris-tribuloides-partial/1-725 328 -S-----HSRFTFGRN 337
BRAF-Strongylocentrotus-purpuratus/1-803 317 -A-----FSRFAFPVKS--SLNPNGHTASAAV 340
BRAF-Paracentrotus-lividus-RNAseq/1-746 347 -A-----FSRFAFPAKS--SLNPSL 363
BRAF-Paracentrotus-lividus-contigEST/1-716 317 -A-----FSRFAFPTKS--SLNPSL 333
BRAF-Saccoglossus-kowaleskii/1-733 296 QL-----LSRHHRAAQS--SAGPDYS-ARQ 317
RAF-Parastichopus-parvimensis/1-747 324 K------YSTKH-EY----------SNQRHH 337
BRAF-Branchiostoma-floridae/1-831 363 LG-----YGRHLQQYQQ--LDSPTQAAAQH 385
BRAF-Trichoplax-adherens-partial/1-325
BRAF-Patiria-miniata-partial/1-819 381 SK-----F-----VFK---QNPADLGARHT 397
KSR1-Caenorhabditis-elegans/1-771 306 EPA 308
KSR2-Caenorhabditis-elegans/1-550 182 TSP 184
KSR1-Amphimedon-queenslandica/1-1246 675 HPPAPSS-PAHDHAPFSSSHS-TS------R 697
KSR1-Patiria-miniata/1-787 442 GQ 443
KSR1-Anneissia-japonica/1-885 450 QQ 451
KSR1-Parastichopus-parvimensis/1-879 450 D 450
KSR1-Eucidaris-tribuloides/1-873 441 A 441
KSR1-Strongylocentrotus-purpuratus/1-869 438 T 438
KSR1-Paracentrotus-lividus/1-848
KSR1-Trichoplax-adherens/1-966
KSR1-Schmidtea-mediterranea/1-954
KSR1-Priapulus-caudatus-partial/1-248 198 QEL---LMLC 204
KSR1-Clytia-hemispherica/1-881 416 DKHMS-SSHGSLMHNTVGN 433
KSR1-Nematostella-vectensis-partial/1-917 440 KQRKVQN-RSVGDLYFNSVDS 459
KSR1-Acropora-millepora/1-957 496 KGRAN-RSHGDL--NSVDQ 511
KSR1-Drosophila-melanogaster/1-966
KSR1-Crassostrea-gigas/1-859 427 GIHL 430
KSR1-Saccoglossus-kowalevskii/1-898
KSR1-Branchiostoma-floridae/1-865 427 RIVC 430
KSR1-Capitella-teleta/1-536 75 DG 76
KSR1-Rhipicephalus-sanguineus/1-864 425 SR 426
KSR1-Limulus-polyphemus/1-603 163 RS 164
KSR3-Ramazzottius-variornatus-partial/1-451
KSR2-Homo-sapiens/1-950 506 KDHIPVP 512
KSR2-Danio-rerio/1-966 521 KDHIPVP 527
KSR1-Homo-sapiens/1-923 433 KEHPPAM 439
KSR1-Danio-rerio/1-898 415 KDQPPVL 421
KSR3-Clytia-hemispherica/1-517
KSR3-Schmidtea-mediterranea/1-666 159 ------IKTTFYKLFDPKSKS 173
KSR3-Rhipicephalus-sanguineus/1-675 172 --LAVTGLLKVVHWLSQRRKSARPDA---GKQHGRSRL------------ADERL 209
KSR3-Limulus-polyphemus/1-686 125 --LALSGLLKVVHWLAQRRRNRDMSKANAADKHAKAKYGLNQCK------VSDKQ 171
KSR3-Limulus-polyphemus-isoformB/1-671 121 --FALSSLQKVVHWLAQRRRNREMSKANAADKHAKAKYGLNQGN------LSEKQ 167
KSR3-Lingula-anatina/1-626 126 ---LYRLYRRLRKFVLPKRQEEEEI-----KDEDS-SSELE 157
KSR3-Crassostrea-gigas/1-585 100 QKSHTSLFRRFYIWMNSK--CRVENRQKSHAEDTSTS-KFH 137
KSR3-Nematostella-vectensis/1-640 109 QKLINGLYDSFCYSPRQA-----LDLYPSVNTHATTKATVH-RQQADSPLAC 154
KSR3-Acropora-millepora/1-634 110 RKLINGIYESFCYSTRQA-----FEFQANDADEFR--------AASRPPLVVD 149
KSR3-Priapulus-caudatus/1-533 98 -KKEHGSYCRLRRWLALRKEKKSV-----ADDEATRCPEQH 132
KSR3-Saccoglossus-kowaleskii/1-591 129 QD--NCSRH--RDISDD 141
KSR3-Ptychodera-flava/1-629 148 DDSARCVQSSANSANDSA 165
KSR3-Trichoplax-adherens/1-510 21 LRKQQDRFSNSCHFSELNQSH--------TVDESLLCR 50
KSR3-Capitella-teleta/1-642 128 ------LYTRFVDWVEKTRSGQHS-----HRDRASSV-------ASSYLSRSRSS 164
KSR3-Eucidaris-tribuloides/1-545 123 VDRRRAC-SAN-RF 134
KSR3-Strongylocentrotus-purpuratus/1-602 125 IETHQSTFSVR-RS 137
KSR3-Paracentrotus-lividus-RNAseq/1-600 124 IESHQSNFSGR-RS 136
KSR3-Notospermus-geniculatus/1-691 168 -----NLYDKFCQWIASRRK--------DHPDNVSMCSSGA-ASTRFDCSYEEVC 208
KSR3-Aplysia-californica/1-405
KSR3-Amphiura-filiformis-partial/1-642 195 LRGQFGSYSGSCTTPSPSSVCRRLRLSPDHHRRPSQC 231
KSR3-Platynereis-dumerilii/1-704 186 ------LYAKFCQWVATN-------------KHP--GSSRE-GSANHTPRRSRSS 218
KSR3-Patiria-miniata/1-779 269 ---SSNVSSVSCGYYSYTSECNSEVFQAQEEDQACLCSSGH-GRATAELSRAERQR 320
KSR3-Anneissia-japonica/1-590 123 ------V-------------------------VTSPCSCITPS-GASTSTPLCSE-AP 147
KSR3-Parastichopus-parvimensis-partial/1-330

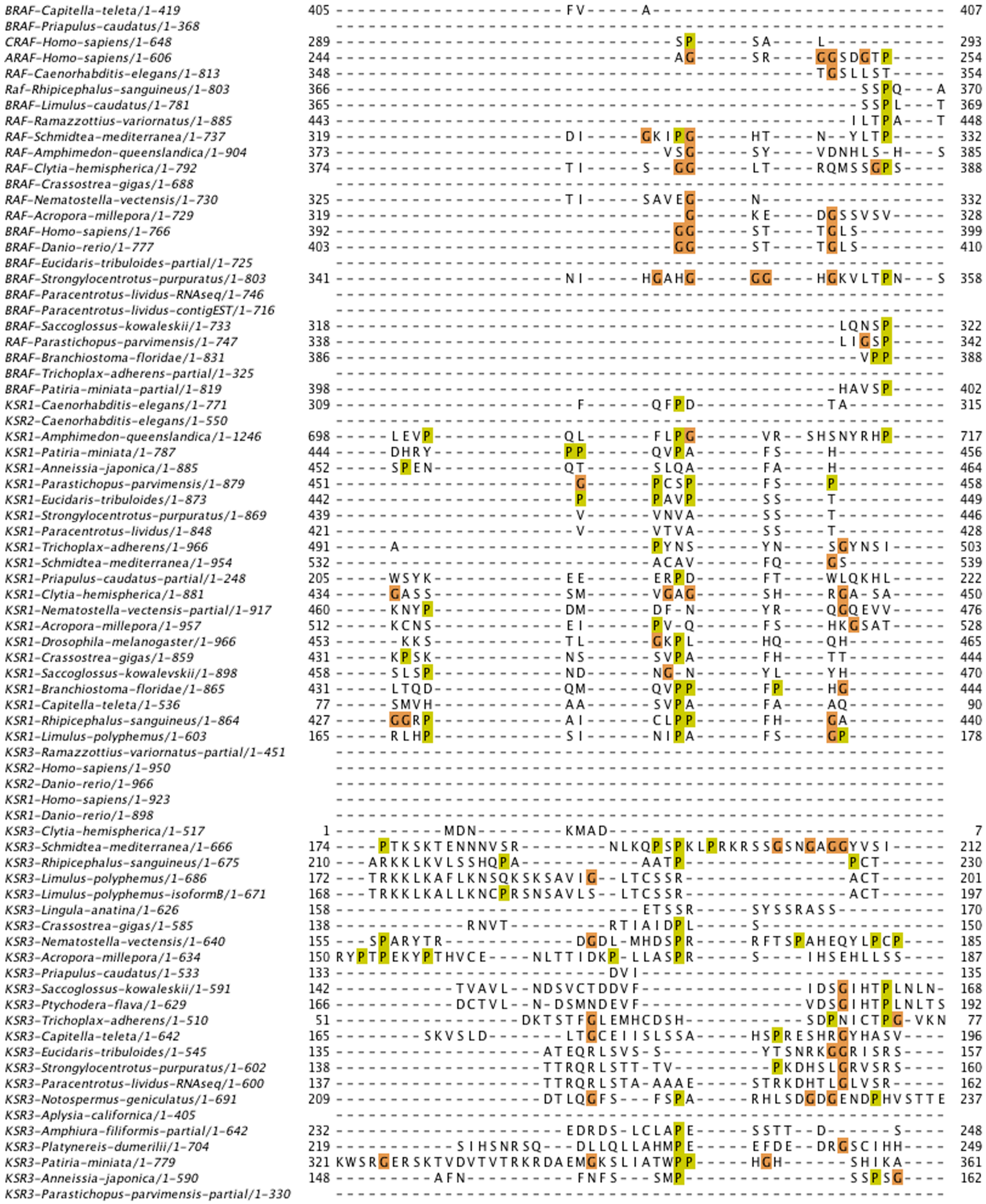
BRAF-Capitella-teleta/1-419
BRAF-Priapulus-caudatus/1-368
CRAF-Homo-sapiens/1-648
ARAF-Homo-sapiens/1-606
RAF-Caenorhabditis-elegans/1-813
Raf-Rhipicephalus-sanguineus/1-803
BRAF-Limulus-caudatus/1-781
RAF-Ramazzottius-variornatus/1-885
RAF-Schmidtea-mediterranea/1-737
RAF-Amphimedon-queenslandica/1-904
RAF-Clytia-hemispherica/1-792
BRAF-Crassostrea-gigas/1-688
RAF-Nematostella-vectensis/1-730
RAF-Acropora-millepora/1-729
BRAF-Homo-sapiens/1-766
BRAF-Danio-rerio/1-777
BRAF-Eucidaris-tribuloides-partial/1-725
BRAF-Strongylocentrotus-purpuratus/1-803
BRAF-Paracentrotus-lividus-RNAseq/1-746
BRAF-Paracentrotus-lividus-contigEST/1-716
BRAF-Saccoglossus-kowaleskii/1-733
RAF-Parastichopus-parvimensis/1-747
BRAF-Branchiostoma-floridae/1-831
BRAF-Trichoplax-adherens-partial/1-325
BRAF-Patiria-miniata-partial/1-819
KSR1-Caenorhabditis-elegans/1-771
KSR2-Caenorhabditis-elegans/1-550
KSR1-Amphimedon-queenslandica/1-1246
KSR1-Patiria-miniata/1-787
KSR1-Anneissia-japonica/1-885
KSR1-Parastichopus-parvimensis/1-879
KSR1-Eucidaris-tribuloides/1-873
KSR1-Strongylocentrotus-purpuratus/1-869
KSR1-Paracentrotus-lividus/1-848
KSR1-Trichoplax-adherens/1-966
KSR1-Schmidtea-mediterranea/1-954
KSR1-Priapulus-caudatus-partial/1-248
KSR1-Clytia-hemispherica/1-881
KSR1-Nematostella-vectensis-partial/1-917
KSR1-Acropora-millepora/1-957
KSR1-Drosophila-melanogaster/1-966
KSR1-Crassostrea-gigas/1-859
KSR1-Saccoglossus-kowalevskii/1-898
KSR1-Branchiostoma-floridae/1-865
KSR1-Capitella-teleta/1-536
KSR1-Rhipicephalus-sanguineus/1-864
KSR1-Limulus-polyphemus/1-603
KSR3-Ramazzottius-variornatus-partial/1-451
KSR2-Homo-sapiens/1-950
KSR2-Danio-rerio/1-966
KSR1-Homo-sapiens/1-923
KSR1-Danio-rerio/1-898
KSR3-Clytia-hemispherica/1-517
KSR3-Schmidtea-mediterranea/1-666
KSR3-Rhipicephalus-sanguineus/1-675
KSR3-Limulus-polyphemus/1-686
KSR3-Limulus-polyphemus-isoformB/1-671
KSR3-Lingula-anatina/1-626
KSR3-Crassostrea-gigas/1-585
KSR3-Nematostella-vectensis/1-640
KSR3-Acropora-millepora/1-634
KSR3-Priapulus-caudatus/1-533
KSR3-Saccoglossus-kowaleskii/1-591
KSR3-Ptychodera-flava/1-629
KSR3-Trichoplax-adherens/1-510
KSR3-Capitella-teleta/1-642
KSR3-Eucidaris-tribuloides/1-545
KSR3-Strongylocentrotus-purpuratus/1-602
KSR3-Paracentrotus-lividus-RNAseq/1-600
KSR3-Notospermus-geniculatus/1-691
KSR3-Aplysia-californica/1-405
KSR3-Amphiura-filiformis-partial/1-642
KSR3-Platynereis-dumerilii/1-704
KSR3-Patiria-miniata/1-779
KSR3-Anneissia-japonica/1-590
KSR3-Parastichopus-parvimensis-partial/1-330

```
BRAF-Capitella-teleta/1-419                             --------------------------------------------------------
BRAF-Priapulus-caudatus/1-368                           --------------------------------------------------------
CRAF-Homo-sapiens/1-648                                 --------------------------------------------------------
ARAF-Homo-sapiens/1-606                                 --------------------------------------------------------
RAF-Caenorhabditis-elegans/1-813                    355 ----------------------------QARHRPHFQS-----GHILSGARMNRLH 377
Raf-Rhipicephalus-sanguineus/1-803                  371 ARSFWCNGF-------PS-----LSYSPPPRGTPSS-------------------- 394
BRAF-Limulus-caudatus/1-781                         370 PRYEIFSRF-------HT-----HKGS--PTHSPVP-------------------- 391
RAF-Ramazzottius-variornatus/1-885                  449 A----GLPW-------PAFNFAFSSRYAPLRFVPFT-------------------- 473
RAF-Schmidtea-mediterranea/1-737                        --------------------------------------------------------
RAF-Amphimedon-queenslandica/1-904                  386 TEYNSVFDY-------NVSSSGNSSRDAPIAHPPHSRRGLCGRGHIST-SALETSH 433
RAF-Clytia-hemispherica/1-792                           --------------------------------------------------------
BRAF-Crassostrea-gigas/1-688                            --------------------------------------------------------
RAF-Nematostella-vectensis/1-730                        --------------------------------------------------------
RAF-Acropora-millepora/1-729                        329 --------L-------E--------------------------------------- 330
BRAF-Homo-sapiens/1-766                                 --------------------------------------------------------
BRAF-Danio-rerio/1-777                                  --------------------------------------------------------
BRAF-Eucidaris-tribuloides-partial/1-725                --------------------------------------------------------
BRAF-Strongylocentrotus-purpuratus/1-803            359 AVQDGVFDY-------DPTNIAND-------------------G-----------H 377
BRAF-Paracentrotus-lividus-RNAseq/1-746                 --------------------------------------------------------
BRAF-Paracentrotus-lividus-contigEST/1-716              --------------------------------------------------------
BRAF-Saccoglossus-kowaleskii/1-733                      --------------------------------------------------------
RAF-Parastichopus-parvimensis/1-747                     --------------------------------------------------------
BRAF-Branchiostoma-floridae/1-831                       --------------------------------------------------------
BRAF-Trichoplax-adherens-partial/1-325                  --------------------------------------------------------
BRAF-Patiria-miniata-partial/1-819                      --------------------------------------------------------
KSR1-Caenorhabditis-elegans/1-771                   316 -------IDSSS----STNSSAPSTPALPA---GISG------------------- 338
KSR2-Caenorhabditis-elegans/1-550                   185 -------------------TCSTSTTMSPA---GVEK------------------- 199
KSR1-Amphimedon-queenslandica/1-1246                718 -------HHHRV----PPHS-TSSSPST------SSQ------------------- 736
KSR1-Patiria-miniata/1-787                          457 -------GESSS----NPSSTNSSTSSSPV---PFTA------------------- 479
KSR1-Anneissia-japonica/1-885                       465 -------GESSS----NPSSTTSSTPSSPV---PLYP------------------- 487
KSR1-Parastichopus-parvimensis/1-879                459 -------GESSS----NPSSTTSSTPSSPN---PYAN------------------- 481
KSR1-Eucidaris-tribuloides/1-873                    450 -------GDSSS----NPSSTTSSTPSSPA---PFAT------------------- 472
KSR1-Strongylocentrotus-purpuratus/1-869            447 -------GDSSS----NPSSTTSSTPSSPA---PFIS------------------- 469
KSR1-Paracentrotus-lividus/1-848                    429 -------GDSSS----NPSSTTSSTPSSPA---PFNS------------------- 451
KSR1-Trichoplax-adherens/1-966                      504 -------NSVST----NDSSTNNLTFKDKH---YLNS------------------- 526
KSR1-Schmidtea-mediterranea/1-954                   540 ------------------DSNSNSTPESPL---NIES------------------- 555
KSR1-Priapulus-caudatus-partial/1-248               223 -------E---K----LPKKRLARSPSHPV---KLSR------------------- 242
KSR1-Clytia-hemispherica/1-881                      451 -------D--TY----STASSTSSISSST--------------------------- 466
KSR1-Nematostella-vectensis-partial/1-917           477 -------NGGSG----STS-SASSPPSSPV---PSSG------------------- 498
KSR1-Acropora-millepora/1-957                       529 -------NGDSG----STSSSTSSPPASPL---PSAA------------------- 551
KSR1-Drosophila-melanogaster/1-966                  466 -------GDSSS----PSSSCTSSTPSSPA---LFQQ------------------- 488
KSR1-Crassostrea-gigas/1-859                        445 -------DS-GS----NPSSCNSSTPSSPT---TMRY------------------- 466
KSR1-Saccoglossus-kowalevskii/1-898                 471 -------MESSS----NPSSTTSSTPSSPA---HFAT------------------- 493
KSR1-Branchiostoma-floridae/1-865                   445 -------DS-SS----NPSSTTSSTPSSPF---QPSS------------------- 466
KSR1-Capitella-teleta/1-536                          91 -------DSGST----TSGSCNSSTPSSPA---PSPS------------------- 113
KSR1-Rhipicephalus-sanguineus/1-864                 441 -------GGPDS----SSSSCNSSTPSSPA---FLMA------------------- 463
KSR1-Limulus-polyphemus/1-603                       179 -------DS-SS----NTSSCNSSTPSSPA---LIIT------------------- 200
KSR3-Ramazzottius-variornatus-partial/1-451             --------------------------------------------------------
KSR2-Homo-sapiens/1-950                             513 -----YQPDSSS----NPSSTTSSTPSSPA---PP--------------------- 535
KSR2-Danio-rerio/1-966                              528 -----YQPDSSS----NPSSTTSSTPSSPA---PP--------------------- 550
KSR1-Homo-sapiens/1-923                             440 -----NHLDSSS----NPSSTTSSTPSSPA---PF--------------------- 462
KSR1-Danio-rerio/1-898                              422 -----NQLDSSS----NPSSTTSSTPSSPA---PF--------------------- 444
KSR3-Clytia-hemispherica/1-517                        8 -------------------KT-----------------------------------   9
KSR3-Schmidtea-mediterranea/1-666                   213 ----------NSGHSEIVTTPGSSIIYDS--------------------------- 231
KSR3-Rhipicephalus-sanguineus/1-675                 231 --CHTR-------KASFAAGILSPNGSLS--------------------------- 250
KSR3-Limulus-polyphemus/1-686                       202 --CGWTRLPITSSQSTNYASLISPNSSLS--------------------------- 228
KSR3-Limulus-polyphemus-isoformB/1-671              198 --CGWNHLPITSSQSTIHASLISPNGSLS--------------------------- 224
KSR3-Lingula-anatina/1-626                          171 ----------SS----CFSITTLSDGYAS--------------------------- 185
KSR3-Crassostrea-gigas/1-585                        151 -----------R----EDNNRANSYRYEET---IHLE------------------- 169
KSR3-Nematostella-vectensis/1-640                   186 --HAWTRTYSCSSRTSNITSFTGSSGYSS--------------------------- 212
KSR3-Acropora-millepora/1-634                       188 --STWYRVNSWS----TSASCTCSSGYSS--------------------------- 210
KSR3-Priapulus-caudatus/1-533                       136 --------------------SLEACATEE--------------------------- 144
KSR3-Saccoglossus-kowaleskii/1-591                  169 ---L-RDRESRS----SYSSSYCSCPRSS--------------------------- 189
KSR3-Ptychodera-flava/1-629                         193 ---L-RDHGSQS----SYSSSYCSCPRSS--------------------------- 213
KSR3-Trichoplax-adherens/1-510                       78 ANFF--G---ST----ADESDLQSVQYHS---------------------------  97
KSR3-Capitella-teleta/1-642                         197 ----------SS-----EDSDFHVYTFAS--------------------------- 210
KSR3-Eucidaris-tribuloides/1-545                    158 --------TLSS----QSSNTSSVSGYHS--------------------------- 174
KSR3-Strongylocentrotus-purpuratus/1-602            161 --------TLSS----QSSNRSSVSGYYS--------------------------- 177
KSR3-Paracentrotus-lividus-RNAseq/1-600             163 ---------LSS----ESSNRSSVSGYFS--------------------------- 178
KSR3-Notospermus-geniculatus/1-691                  238 TDLFWYQRTRQS----SFSSTYEVCSVAS--------------------------- 262
KSR3-Aplysia-californica/1-405                          --------------------------------------------------------
KSR3-Amphiura-filiformis-partial/1-642              249 --------PANN----ASVRTMVSYAEYV--------------------------- 265
KSR3-Platynereis-dumerilii/1-704                    250 --------SLSS----EESELYESLKRHH--------------------------- 266
KSR3-Patiria-miniata/1-779                          362 --------GTSS----NVSS--VSCGYYS--------------------------- 376
KSR3-Anneissia-japonica/1-590                       163 --------TQSS----QTSS--SSCGYYS--------------------------- 177
KSR3-Parastichopus-parvimensis-partial/1-330            --------------------------------------------------------
```

BRAF-Capitella-teleta/1-419	408	-ALACMLPGELSL--	419
BRAF-Priapulus-caudatus/1-368		---	
CRAF-Homo-sapiens/1-648		---	
ARAF-Homo-sapiens/1-606		---	
RAF-Caenorhabditis-elegans/1-813	378	PLVDCTPLGSNSPSS----TCSSP-PG--------------------GLI-------	402
Raf-Rhipicephalus-sanguineus/1-803	395	---------SSHHL---TLPHPSGSA-Y-----------------KRHAFAYASQR-	420
BRAF-Limulus-caudatus/1-781	392	---------KRRQL---ELPCPGGSP-G-----------------S-LALPYQQ---	414
RAF-Ramazzottius-variornatus/1-885	474	--DLIASQQ	480
RAF-Schmidtea-mediterranea/1-737	333	------SLSSGSHNA--TSNHSSGHNSA-----------------NNSYSNFY----	360
RAF-Amphimedon-queenslandica/1-904	434	TGMDAMRIERRRKGH---------KRQS-----------------SAGHLDYSGVED	464
RAF-Clytia-hemispherica/1-792	389	-------SGRGRITNTLFTPTSVSAPNS-----------------GENYFDFNTANS	421
BRAF-Crassostrea-gigas/1-688	305	--------KEKRHAS--DGTCSKPTPKH-----------------RYGSQ-------	327
RAF-Nematostella-vectensis/1-730	333	-----RLMRSSRHGR--HKSESATHPRI-----------------NDIFHDNNVHP-	364
RAF-Acropora-millepora/1-729	331	----SSKLPNRRHSR--TSSVGTANPRL-----------------------Y-----	353
BRAF-Homo-sapiens/1-766		---	
BRAF-Danio-rerio/1-777		---	
BRAF-Eucidaris-tribuloides-partial/1-725	338	---LQ	339
BRAF-Strongylocentrotus-purpuratus/1-803	378	TASAAVNIHGAHGGG--HGKVLTPNSAV-----------------QDGVFDYDPTNI	415
BRAF-Paracentrotus-lividus-RNAseq/1-746		---	
BRAF-Paracentrotus-lividus-contigEST/1-716		---	
BRAF-Saccoglossus-kowaleskii/1-733	323	----C--------------KTRQTPTPS-----------------KDSVFDYSPASI	344
RAF-Parastichopus-parvimensis/1-747	343	----MPSRKNK--------------AQH-----------------QEGYFDYNVLA-	363
BRAF-Branchiostoma-floridae/1-831	389	----FASKSNRRHAS--VSSTPT-HTAM-----------------EVSVFDYSPAAI	421
BRAF-Trichoplax-adherens-partial/1-325		---	
BRAF-Patiria-miniata-partial/1-819	403	----AVSVARPR--Q--ISFTPTILSAG-----------------RDTVFDYSPTSA	434
KSR1-Caenorhabditis-elegans/1-771	339	-------------------NVSSLTAPYR----------------SERKFLFPDTEN	360
KSR2-Caenorhabditis-elegans/1-550	200	-------------------NVHKT---------------------------------	204
KSR1-Amphimedon-queenslandica/1-1246	737	------------SS------PCLTPMSPRSPLDPPVEYSGFNSLHRSIAIVLSTDDN	775
KSR1-Patiria-miniata/1-787	480	------------SSSSTT----P-PTPAHPSPS---PHMDPCK----PQFQ------	506
KSR1-Anneissia-japonica/1-885	488	------------SSASAT----PPSV---HS-----PHVR---E---FDFGFVNDVT	514
KSR1-Parastichopus-parvimensis/1-879	482	------------SSASPT----PSS-SIQASPN---PG--L--E---SQFTFPDVTV	511
KSR1-Eucidaris-tribuloides/1-873	473	------------PSSSSI----TPPSPAQPSPR---ISSS---V---PQFKFPDITP	504
KSR1-Strongylocentrotus-purpuratus/1-869	470	------------TSGIPL----PSPSPALPSPS---PRMM---V---PQFKFPPDVS	501
KSR1-Paracentrotus-lividus/1-848	452	------------TPGMPP------PSPAQPSPS---PLMS---V---PQFKFPPDVS	481
KSR1-Trichoplax-adherens/1-966	527	------------QGAD----------KIKSIPNSAPPTSVNS------TFNFSTPTD	555
KSR1-Schmidtea-mediterranea/1-954	556	------------GYNSAVNNHISPR-----------HPNKLLKT---QKFDFSKIDE	586
KSR1-Priapulus-caudatus-partial/1-248	243	------------SAES--------------------------------VF-------	248
KSR1-Clytia-hemispherica/1-881	467	---FHSDSPLG	474
KSR1-Nematostella-vectensis-partial/1-917	499	------------GRAD-----YSPGE----------EQDGFPTQ---DHFYYPSRYG	525
KSR1-Acropora-millepora/1-957	552	------------SVSTS--TCLSAQE----------ELDQFPGS---H-FPFS-DVS	579
KSR1-Drosophila-melanogaster/1-966	489	------------REREL--DQAGSSSSANLLPTPSLGKHQPS------QFNFPNVTV	525
KSR1-Crassostrea-gigas/1-859	467	-----------------------TPSP---------ANTQNLT-----TFTFPEIVE	486
KSR1-Saccoglossus-kowalevskii/1-898	494	------------SSTTS--SISSPAQP---------SPLTRQT-----HFDFPEPNN	522
KSR1-Branchiostoma-floridae/1-865	467	-----------------------SDSQ---------PSPMTRQ-----AFRFPVLVK	486
KSR1-Capitella-teleta/1-536	114	------------SAVMM--S--SGGSM---------ASPQFGS-----QFTFPEIST	140
KSR1-Rhipicephalus-sanguineus/1-864	464	------------ASR--------QNTP---------SSASRLQ-----QFHFPEILS	486
KSR1-Limulus-polyphemus/1-603	201	------------S-A--------QQTP---------PSASRLE-----QFQFPDLNN	222
KSR3-Ramazzottius-variornatus-partial/1-451	1	-----------------METCSATTPANK----------------------------	12
KSR2-Homo-sapiens/1-950	536	-------L---------PPSATPPSPLHP---SPQC-------TRQQKNFNLPA---	563
KSR2-Danio-rerio/1-966	551	-------L---------PPSATPPSPLHP---SPQS-------ARQQKQSNLTA---	578
KSR1-Homo-sapiens/1-923	463	-------P---------TSSNPSSATTPP---NPSP-------GQRDSRFNFPA---	490
KSR1-Danio-rerio/1-898	445	-------Q---------QSNPPSVTPPPN---PSPK-------GHRDNRFHFPA---	472
KSR3-Clytia-hemispherica/1-517	10	-----------------VTTCASMLYALF------QK--------RKRKCSCLCHTA	35
KSR3-Schmidtea-mediterranea/1-666	232	-------L---SADSGRQNDVSSRSGS-------------------TRNSIYSLD--	257
KSR3-Rhipicephalus-sanguineus/1-675	251	-----------------SSGYISSSASSA-VQTPS-----------DTELDCRGA--	276
KSR3-Limulus-polyphemus/1-686	229	-----------------SSSCLSS-TSSA-AQTPS-----------DTESDYRIR--	253
KSR3-Limulus-polyphemus-isoformB/1-671	225	-----------------SSSCLSS-TSSA-AQTPS-----------DTESDYRIR--	249
KSR3-Lingula-anatina/1-626	186	-------R-------SSVSTCSSMHFKPPTLLPPQP---------QQTESLS-----	214
KSR3-Crassostrea-gigas/1-585	170	------PV---GCDVNYVYICNKSSETVV------QE--------QNTNDVC-----	198
KSR3-Nematostella-vectensis/1-640	213	-------M---------PASCPSSANSHR----------------------------	225
KSR3-Acropora-millepora/1-634	211	-------A---------NTSCPSSANSHR----------------------------	223
KSR3-Priapulus-caudatus/1-533	145	-------K------------------T------------------------------	146
KSR3-Saccoglossus-kowaleskii/1-591	190	-------I------------------ASD------TA---------NQCCLCET---	203
KSR3-Ptychodera-flava/1-629	214	-------V------------------ASI------P----------HQNCTCLT---	226
KSR3-Trichoplax-adherens/1-510	98	-------Q------ASYTNDEDSLL--------------------------------	109
KSR3-Capitella-teleta/1-642	211	-------Y---------SSNCSSLTLTSL------ST----------TASDYPSSHV	235
KSR3-Eucidaris-tribuloides/1-545	175	-------L---ESDSSFTSDCETPTELQV------FD---------VGAEGCGF---	203
KSR3-Strongylocentrotus-purpuratus/1-602	178	-------Q---ESDASLLSECDTPKEISV------FD---------SFGPGDVT---	206
KSR3-Paracentrotus-lividus-RNAseq/1-600	179	-------L---------ESECDTPKELSV------FD---------SFGPADVV---	201
KSR3-Notospermus-geniculatus/1-691	263	-------L---------STSTSYSEILHD------RD---------REYFNFAG---	285
KSR3-Aplysia-californica/1-405		---	
KSR3-Amphiura-filiformis-partial/1-642	266	-------L---------SDNCSDVFVTDS------F-----------------G---	280
KSR3-Platynereis-dumerilii/1-704	267	-------Y---------SSTCSSLTITST-------S---------TTTSDCPG---	288
KSR3-Patiria-miniata/1-779	377	-------Y---------TSECNSDVFRTQ------EE---------DQACLCSS---	399
KSR3-Anneissia-japonica/1-590	178	-------L---------GSECNVSTVDSS------NG---------ITSCTC-----	198
KSR3-Parastichopus-parvimensis-partial/1-330		---	

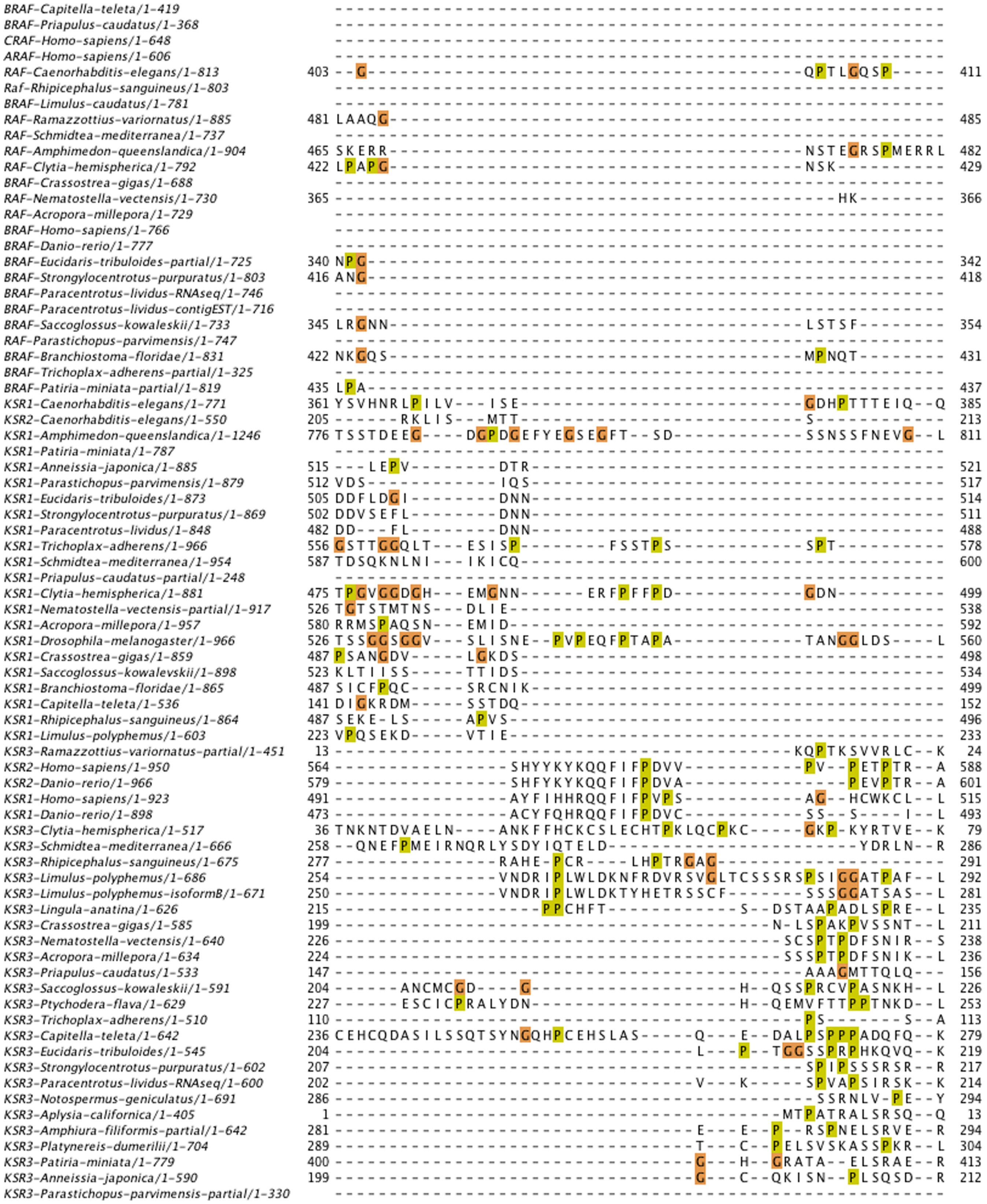
BRAF-Capitella-teleta/1-419
BRAF-Priapulus-caudatus/1-368
CRAF-Homo-sapiens/1-648
ARAF-Homo-sapiens/1-606
RAF-Caenorhabditis-elegans/1-813
Raf-Rhipicephalus-sanguineus/1-803
BRAF-Limulus-caudatus/1-781
RAF-Ramazzottius-variornatus/1-885
RAF-Schmidtea-mediterranea/1-737
RAF-Amphimedon-queenslandica/1-904
RAF-Clytia-hemispherica/1-792
BRAF-Crassostrea-gigas/1-688
RAF-Nematostella-vectensis/1-730
RAF-Acropora-millepora/1-729
BRAF-Homo-sapiens/1-766
BRAF-Danio-rerio/1-777
BRAF-Eucidaris-tribuloides-partial/1-725
BRAF-Strongylocentrotus-purpuratus/1-803
BRAF-Paracentrotus-lividus-RNAseq/1-746
BRAF-Paracentrotus-lividus-contigEST/1-716
BRAF-Saccoglossus-kowaleskii/1-733
RAF-Parastichopus-parvimensis/1-747
BRAF-Branchiostoma-floridae/1-831
BRAF-Trichoplax-adherens-partial/1-325
BRAF-Patiria-miniata-partial/1-819
KSR1-Caenorhabditis-elegans/1-771
KSR2-Caenorhabditis-elegans/1-550
KSR1-Amphimedon-queenslandica/1-1246
KSR1-Patiria-miniata/1-787
KSR1-Anneissia-japonica/1-885
KSR1-Parastichopus-parvimensis/1-879
KSR1-Eucidaris-tribuloides/1-873
KSR1-Strongylocentrotus-purpuratus/1-869
KSR1-Paracentrotus-lividus/1-848
KSR1-Trichoplax-adherens/1-966
KSR1-Schmidtea-mediterranea/1-954
KSR1-Priapulus-caudatus-partial/1-248
KSR1-Clytia-hemispherica/1-881
KSR1-Nematostella-vectensis-partial/1-917
KSR1-Acropora-millepora/1-957
KSR1-Drosophila-melanogaster/1-966
KSR1-Crassostrea-gigas/1-859
KSR1-Saccoglossus-kowalevskii/1-898
KSR1-Branchiostoma-floridae/1-865
KSR1-Capitella-teleta/1-536
KSR1-Rhipicephalus-sanguineus/1-864
KSR1-Limulus-polyphemus/1-603
KSR3-Ramazzottius-variornatus-partial/1-451
KSR2-Homo-sapiens/1-950
KSR2-Danio-rerio/1-966
KSR1-Homo-sapiens/1-923
KSR1-Danio-rerio/1-898
KSR3-Clytia-hemispherica/1-517
KSR3-Schmidtea-mediterranea/1-666
KSR3-Rhipicephalus-sanguineus/1-675
KSR3-Limulus-polyphemus/1-686
KSR3-Limulus-polyphemus-isoformB/1-671
KSR3-Lingula-anatina/1-626
KSR3-Crassostrea-gigas/1-585
KSR3-Nematostella-vectensis/1-640
KSR3-Acropora-millepora/1-634
KSR3-Priapulus-caudatus/1-533
KSR3-Saccoglossus-kowaleskii/1-591
KSR3-Ptychodera-flava/1-629
KSR3-Trichoplax-adherens/1-510
KSR3-Capitella-teleta/1-642
KSR3-Eucidaris-tribuloides/1-545
KSR3-Strongylocentrotus-purpuratus/1-602
KSR3-Paracentrotus-lividus-RNAseq/1-600
KSR3-Notospermus-geniculatus/1-691
KSR3-Aplysia-californica/1-405
KSR3-Amphiura-filiformis-partial/1-642
KSR3-Platynereis-dumerilii/1-704
KSR3-Patiria-miniata/1-779
KSR3-Anneissia-japonica/1-590
KSR3-Parastichopus-parvimensis-partial/1-330

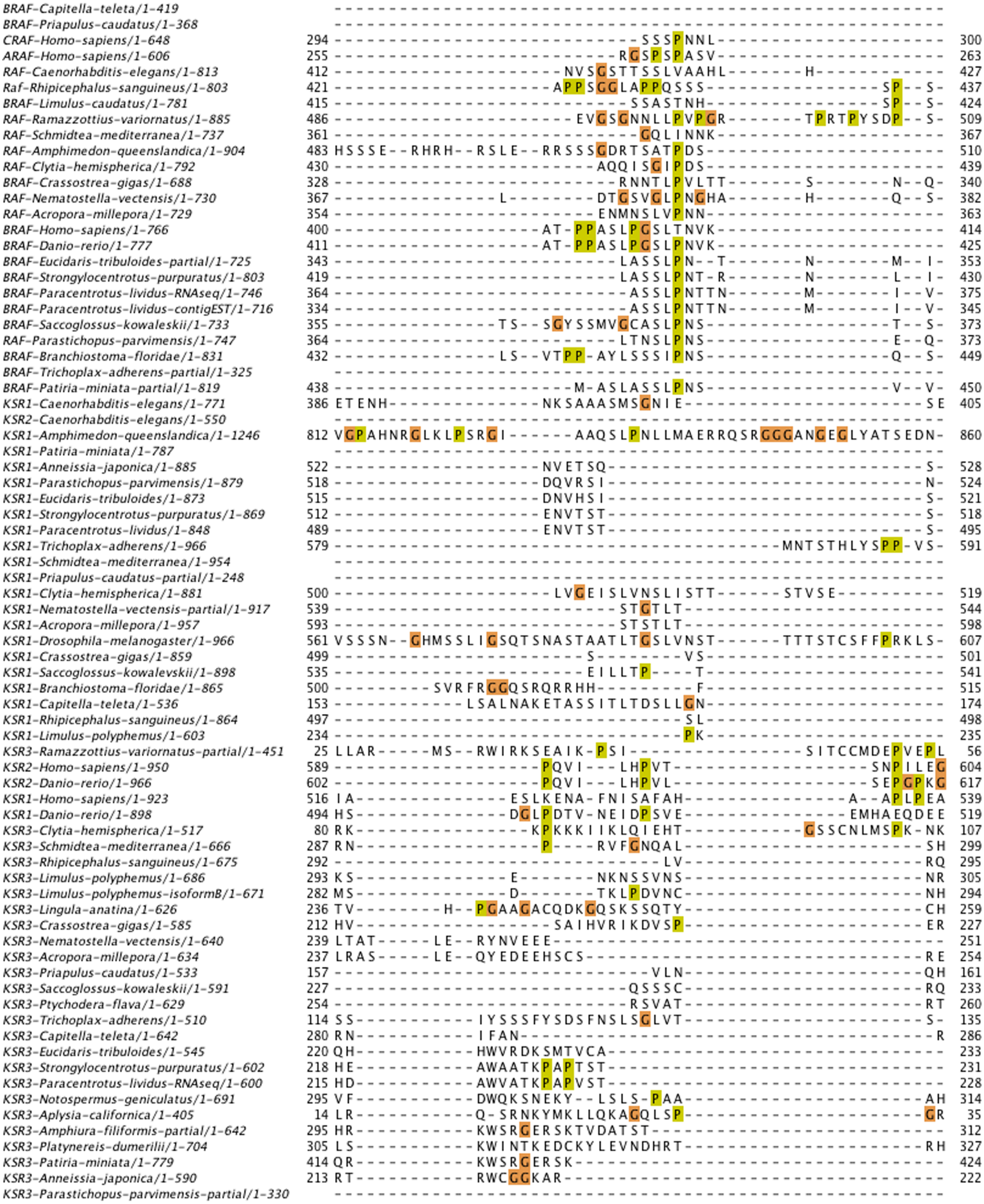

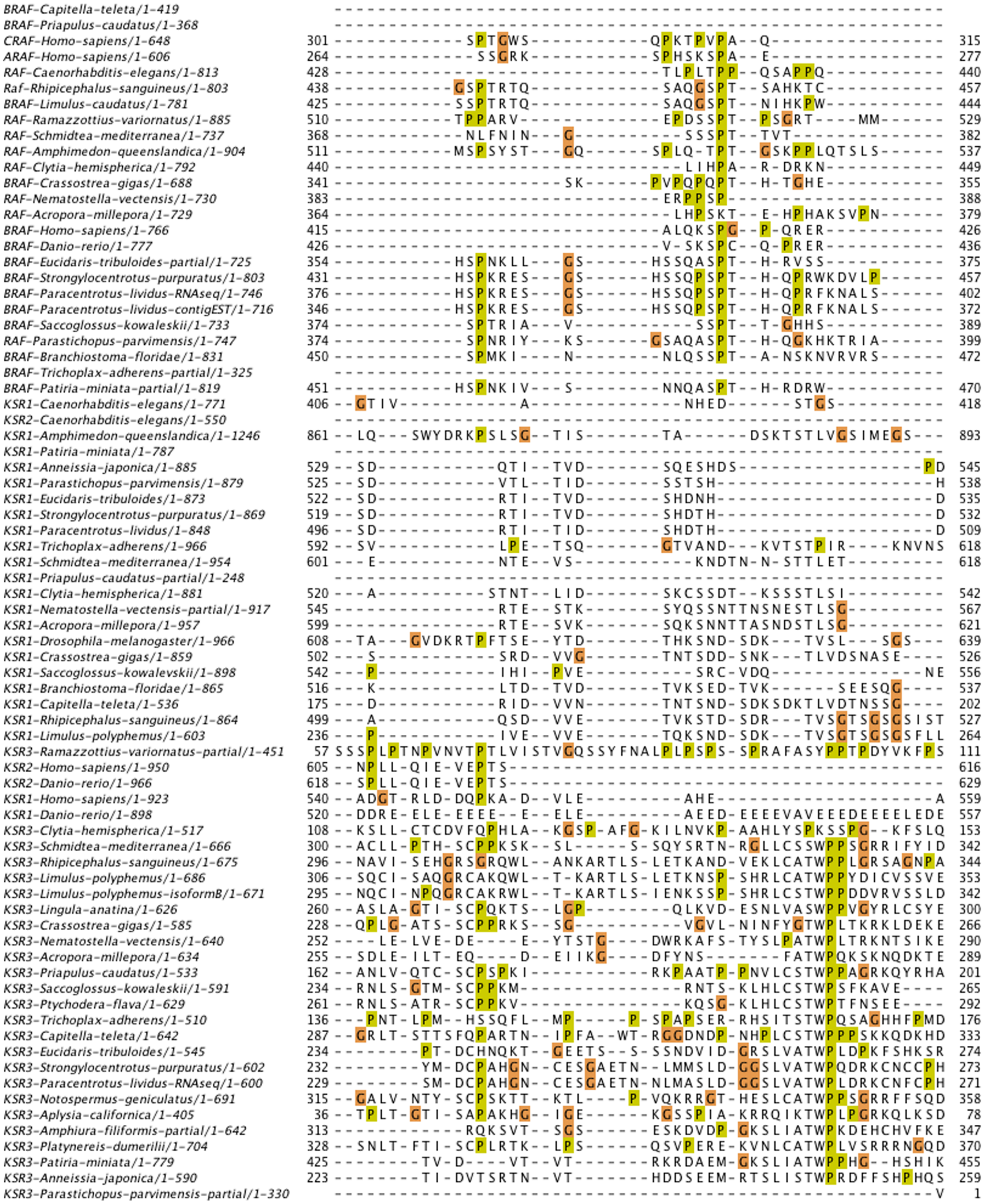
BRAF-Capitella-teleta/1-419
BRAF-Priapulus-caudatus/1-368
CRAF-Homo-sapiens/1-648
ARAF-Homo-sapiens/1-606
RAF-Caenorhabditis-elegans/1-813
Raf-Rhipicephalus-sanguineus/1-803
BRAF-Limulus-caudatus/1-781
RAF-Ramazzottius-variornatus/1-885
RAF-Schmidtea-mediterranea/1-737
RAF-Amphimedon-queenslandica/1-904
RAF-Clytia-hemispherica/1-792
BRAF-Crassostrea-gigas/1-688
RAF-Nematostella-vectensis/1-730
RAF-Acropora-millepora/1-729
BRAF-Homo-sapiens/1-766
BRAF-Danio-rerio/1-777
BRAF-Eucidaris-tribuloides-partial/1-725
BRAF-Strongylocentrotus-purpuratus/1-803
BRAF-Paracentrotus-lividus-RNAseq/1-746
BRAF-Paracentrotus-lividus-contigEST/1-716
BRAF-Saccoglossus-kowaleskii/1-733
RAF-Parastichopus-parvimensis/1-747
BRAF-Branchiostoma-floridae/1-831
BRAF-Trichoplax-adherens-partial/1-325
BRAF-Patiria-miniata-partial/1-819
KSR1-Caenorhabditis-elegans/1-771
KSR2-Caenorhabditis-elegans/1-550
KSR1-Amphimedon-queenslandica/1-1246
KSR1-Patiria-miniata/1-787
KSR1-Anneissia-japonica/1-885
KSR1-Parastichopus-parvimensis/1-879
KSR1-Eucidaris-tribuloides/1-873
KSR1-Strongylocentrotus-purpuratus/1-869
KSR1-Paracentrotus-lividus/1-848
KSR1-Trichoplax-adherens/1-966
KSR1-Schmidtea-mediterranea/1-954
KSR1-Priapulus-caudatus-partial/1-248
KSR1-Clytia-hemispherica/1-881
KSR1-Nematostella-vectensis-partial/1-917
KSR1-Acropora-millepora/1-957
KSR1-Drosophila-melanogaster/1-966
KSR1-Crassostrea-gigas/1-859
KSR1-Saccoglossus-kowalevskii/1-898
KSR1-Branchiostoma-floridae/1-865
KSR1-Capitella-teleta/1-536
KSR1-Rhipicephalus-sanguineus/1-864
KSR1-Limulus-polyphemus/1-603
KSR3-Ramazzottius-variornatus-partial/1-451
KSR2-Homo-sapiens/1-950
KSR2-Danio-rerio/1-966
KSR1-Homo-sapiens/1-923
KSR1-Danio-rerio/1-898
KSR3-Clytia-hemispherica/1-517
KSR3-Schmidtea-mediterranea/1-666
KSR3-Rhipicephalus-sanguineus/1-675
KSR3-Limulus-polyphemus/1-686
KSR3-Limulus-polyphemus-isoformB/1-671
KSR3-Lingula-anatina/1-626
KSR3-Crassostrea-gigas/1-585
KSR3-Nematostella-vectensis/1-640
KSR3-Acropora-millepora/1-634
KSR3-Priapulus-caudatus/1-533
KSR3-Saccoglossus-kowaleskii/1-591
KSR3-Ptychodera-flava/1-629
KSR3-Trichoplax-adherens/1-510
KSR3-Capitella-teleta/1-642
KSR3-Eucidaris-tribuloides/1-545
KSR3-Strongylocentrotus-purpuratus/1-602
KSR3-Paracentrotus-lividus-RNAseq/1-600
KSR3-Notospermus-geniculatus/1-691
KSR3-Aplysia-californica/1-405
KSR3-Amphiura-filiformis-partial/1-642
KSR3-Platynereis-dumerilii/1-704
KSR3-Patiria-miniata/1-779
KSR3-Anneissia-japonica/1-590
KSR3-Parastichopus-parvimensis-partial/1-330

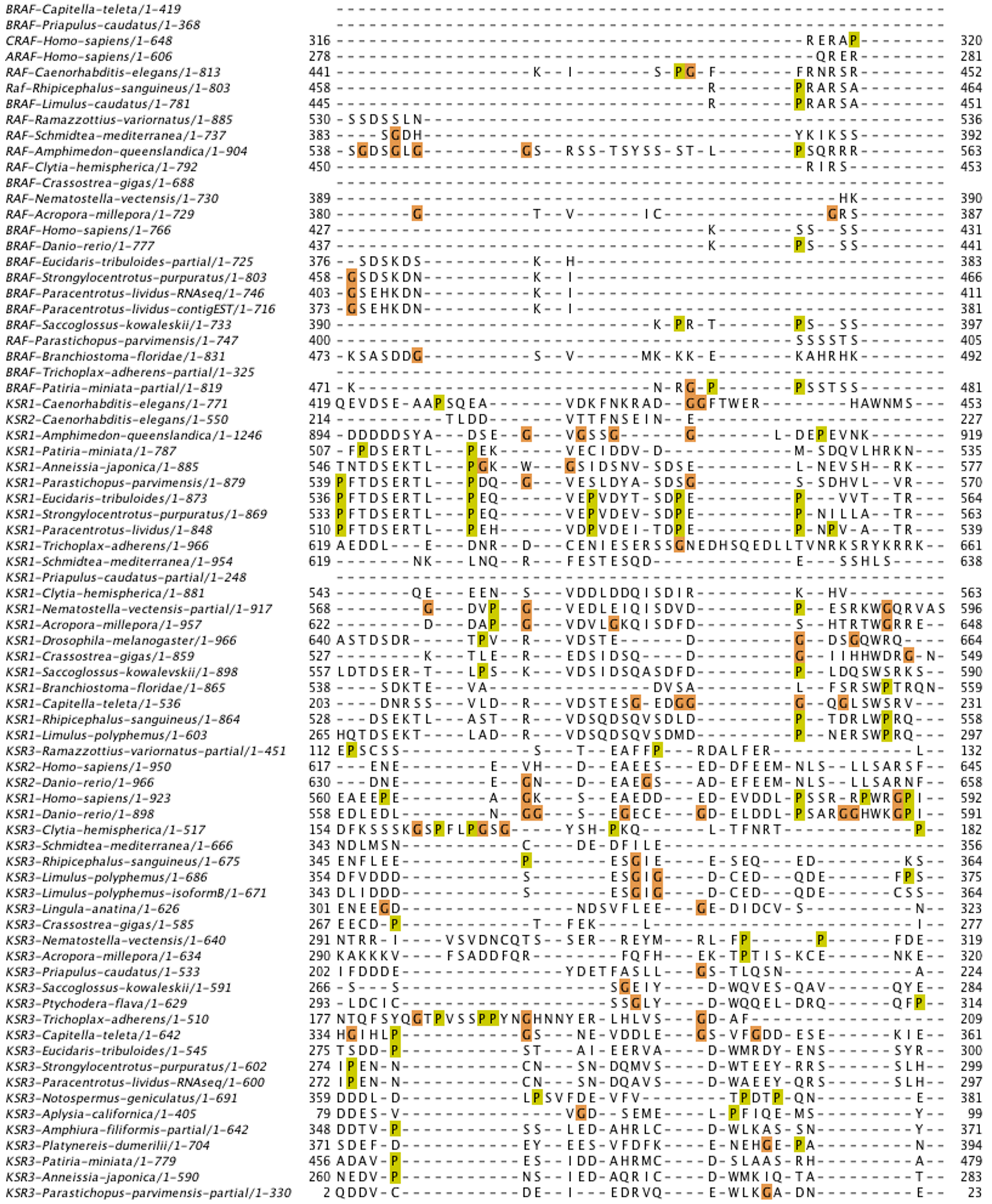

BRAF-Capitella-teleta/1-419
BRAF-Priapulus-caudatus/1-368
CRAF-Homo-sapiens/1-648
ARAF-Homo-sapiens/1-606
RAF-Caenorhabditis-elegans/1-813
Raf-Rhipicephalus-sanguineus/1-803
BRAF-Limulus-caudatus/1-781
RAF-Ramazzottius-variornatus/1-885
RAF-Schmidtea-mediterranea/1-737
RAF-Amphimedon-queenslandica/1-904
RAF-Clytia-hemispherica/1-792
BRAF-Crassostrea-gigas/1-688
RAF-Nematostella-vectensis/1-730
RAF-Acropora-millepora/1-729
BRAF-Homo-sapiens/1-766
BRAF-Danio-rerio/1-777
BRAF-Eucidaris-tribuloides-partial/1-725
BRAF-Strongylocentrotus-purpuratus/1-803
BRAF-Paracentrotus-lividus-RNAseq/1-746
BRAF-Paracentrotus-lividus-contigEST/1-716
BRAF-Saccoglossus-kowaleskii/1-733
RAF-Parastichopus-parvimensis/1-747
BRAF-Branchiostoma-floridae/1-831
BRAF-Trichoplax-adherens-partial/1-325
BRAF-Patiria-miniata-partial/1-819
KSR1-Caenorhabditis-elegans/1-771
KSR2-Caenorhabditis-elegans/1-550
KSR1-Amphimedon-queenslandica/1-1246
KSR1-Patiria-miniata/1-787
KSR1-Anneissia-japonica/1-885
KSR1-Parastichopus-parvimensis/1-879
KSR1-Eucidaris-tribuloides/1-873
KSR1-Strongylocentrotus-purpuratus/1-869
KSR1-Paracentrotus-lividus/1-848
KSR1-Trichoplax-adherens/1-966
KSR1-Schmidtea-mediterranea/1-954
KSR1-Priapulus-caudatus-partial/1-248
KSR1-Clytia-hemispherica/1-881
KSR1-Nematostella-vectensis-partial/1-917
KSR1-Acropora-millepora/1-957
KSR1-Drosophila-melanogaster/1-966
KSR1-Crassostrea-gigas/1-859
KSR1-Saccoglossus-kowalevskii/1-898
KSR1-Branchiostoma-floridae/1-865
KSR1-Capitella-teleta/1-536
KSR1-Rhipicephalus-sanguineus/1-864
KSR1-Limulus-polyphemus/1-603
KSR3-Ramazzottius-variornatus-partial/1-451
KSR2-Homo-sapiens/1-950
KSR2-Danio-rerio/1-966
KSR1-Homo-sapiens/1-923
KSR1-Danio-rerio/1-898
KSR3-Clytia-hemispherica/1-517
KSR3-Schmidtea-mediterranea/1-666
KSR3-Rhipicephalus-sanguineus/1-675
KSR3-Limulus-polyphemus/1-686
KSR3-Limulus-polyphemus-isoformB/1-671
KSR3-Lingula-anatina/1-626
KSR3-Crassostrea-gigas/1-585
KSR3-Nematostella-vectensis/1-640
KSR3-Acropora-millepora/1-634
KSR3-Priapulus-caudatus/1-533
KSR3-Saccoglossus-kowaleskii/1-591
KSR3-Ptychodera-flava/1-629
KSR3-Trichoplax-adherens/1-510
KSR3-Capitella-teleta/1-642
KSR3-Eucidaris-tribuloides/1-545
KSR3-Strongylocentrotus-purpuratus/1-602
KSR3-Paracentrotus-lividus-RNAseq/1-600
KSR3-Notospermus-geniculatus/1-691
KSR3-Aplysia-californica/1-405
KSR3-Amphiura-filiformis-partial/1-642
KSR3-Platynereis-dumerilii/1-704
KSR3-Patiria-miniata/1-779
KSR3-Anneissia-japonica/1-590
KSR3-Parastichopus-parvimensis-partial/1-330

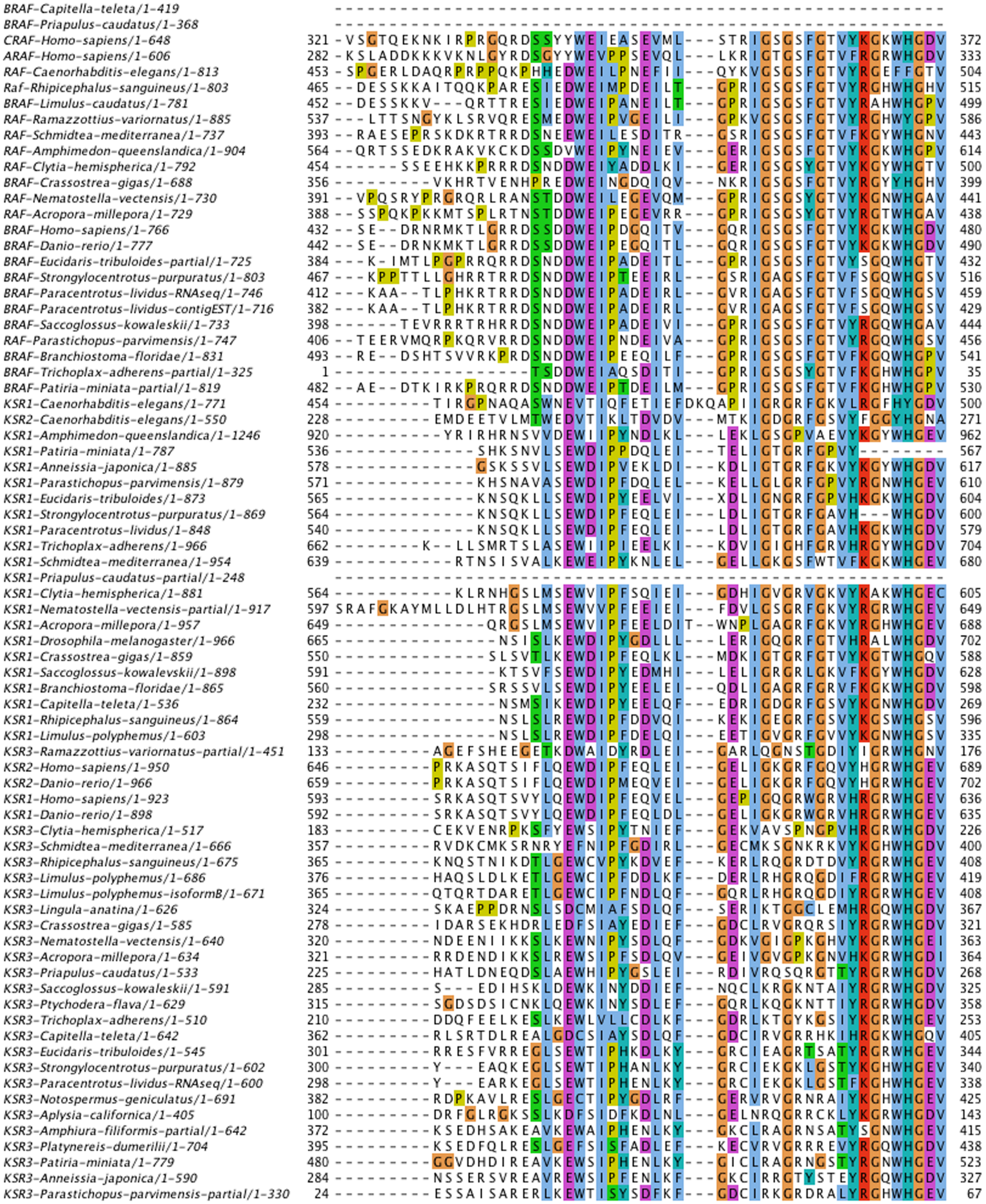
BRAF-Capitella-teleta/1-419
BRAF-Priapulus-caudatus/1-368
CRAF-Homo-sapiens/1-648
ARAF-Homo-sapiens/1-606
RAF-Caenorhabditis-elegans/1-813
Raf-Rhipicephalus-sanguineus/1-803
BRAF-Limulus-caudatus/1-781
RAF-Ramazzottius-variornatus/1-885
RAF-Schmidtea-mediterranea/1-737
RAF-Amphimedon-queenslandica/1-904
RAF-Clytia-hemispherica/1-792
BRAF-Crassostrea-gigas/1-688
RAF-Nematostella-vectensis/1-730
RAF-Acropora-millepora/1-729
BRAF-Homo-sapiens/1-766
BRAF-Danio-rerio/1-777
BRAF-Eucidaris-tribuloides-partial/1-725
BRAF-Strongylocentrotus-purpuratus/1-803
BRAF-Paracentrotus-lividus-RNAseq/1-746
BRAF-Paracentrotus-lividus-contigEST/1-716
BRAF-Saccoglossus-kowaleskii/1-733
RAF-Parastichopus-parvimensis/1-747
BRAF-Branchiostoma-floridae/1-831
BRAF-Trichoplax-adherens-partial/1-325
BRAF-Patiria-miniata-partial/1-819
KSR1-Caenorhabditis-elegans/1-771
KSR2-Caenorhabditis-elegans/1-550
KSR1-Amphimedon-queenslandica/1-1246
KSR1-Patiria-miniata/1-787
KSR1-Anneissia-japonica/1-885
KSR1-Parastichopus-parvimensis/1-879
KSR1-Eucidaris-tribuloides/1-873
KSR1-Strongylocentrotus-purpuratus/1-869
KSR1-Paracentrotus-lividus/1-848
KSR1-Trichoplax-adherens/1-966
KSR1-Schmidtea-mediterranea/1-954
KSR1-Priapulus-caudatus-partial/1-248
KSR1-Clytia-hemispherica/1-881
KSR1-Nematostella-vectensis-partial/1-917
KSR1-Acropora-millepora/1-957
KSR1-Drosophila-melanogaster/1-966
KSR1-Crassostrea-gigas/1-859
KSR1-Saccoglossus-kowalevskii/1-898
KSR1-Branchiostoma-floridae/1-865
KSR1-Capitella-teleta/1-536
KSR1-Rhipicephalus-sanguineus/1-864
KSR1-Limulus-polyphemus/1-603
KSR3-Ramazzottius-variornatus-partial/1-451
KSR2-Homo-sapiens/1-950
KSR2-Danio-rerio/1-966
KSR1-Homo-sapiens/1-923
KSR1-Danio-rerio/1-898
KSR3-Clytia-hemispherica/1-517
KSR3-Schmidtea-mediterranea/1-666
KSR3-Rhipicephalus-sanguineus/1-675
KSR3-Limulus-polyphemus/1-686
KSR3-Limulus-polyphemus-isoformB/1-671
KSR3-Lingula-anatina/1-626
KSR3-Crassostrea-gigas/1-585
KSR3-Nematostella-vectensis/1-640
KSR3-Acropora-millepora/1-634
KSR3-Priapulus-caudatus/1-533
KSR3-Saccoglossus-kowaleskii/1-591
KSR3-Ptychodera-flava/1-629
KSR3-Trichoplax-adherens/1-510
KSR3-Capitella-teleta/1-642
KSR3-Eucidaris-tribuloides/1-545
KSR3-Strongylocentrotus-purpuratus/1-602
KSR3-Paracentrotus-lividus-RNAseq/1-600
KSR3-Notospermus-geniculatus/1-691
KSR3-Aplysia-californica/1-405
KSR3-Amphiura-filiformis-partial/1-642
KSR3-Platynereis-dumerilii/1-704
KSR3-Patiria-miniata/1-779
KSR3-Anneissia-japonica/1-590
KSR3-Parastichopus-parvimensis-partial/1-330

```
BRAF-Capitella-teleta/1-419                          ----------------------------------------------------
BRAF-Priapulus-caudatus/1-368                        ----------------------------------------------------
CRAF-Homo-sapiens/1-648                         373  AVKILKVVD---PTPEQFQAFRNE-VAVLRKTRHVNILLFMGYMTK------DN--   416
ARAF-Homo-sapiens/1-606                         334  AVKVLKVSQ---PTAEQAQAFKNE-MQVLRKTRHVNILLFMGFMTR------PG--   377
RAF-Caenorhabditis-elegans/1-813                505  AIKKLNVVD---PTPSQMAAFKNE-VAVLKKTRHLNVLLFMGWVRE------PE--   548
Raf-Rhipicephalus-sanguineus/1-803              516  ALKKLNVTN---PTPAQLQAFKNE-VSVLRKTRHVSIILFMGCVSK------PQ--   559
BRAF-Limulus-caudatus/1-781                     500  ALKKLNVTD---PTAAQLQAFKNE-VAVLRKTRHVNILLFMGWVSK------PH--   543
RAF-Ramazzottius-variornatus/1-885              587  AIKRLNVND---PTLEQMQAFKNE-VAVLRKTRHNNVLLFMGCVSKCEPGQSPQ--   636
RAF-Schmidtea-mediterranea/1-737                444  AIKELNVSE---PTPLQLKAFKNE-VSMLRKTRHVNILLFMGCIYV------PR--   487
RAF-Amphimedon-queenslandica/1-904              615  AVKKLNVSN---PTEQQMQAFKNE-VAVLMKTRHANILLFMGWTSK------PR--   658
RAF-Clytia-hemispherica/1-792                   501  AVKTLNVKD---PNPQQLLAFQNE-VGVLRKTRHVNVLLFMGCLSS------PN--   544
BRAF-Crassostrea-gigas/1-688                    400  AIKRLNVTA---PTPQQLRAFKNE-VAVLRKTRHTNILLFMGWTSK------PQ--   443
RAF-Nematostella-vectensis/1-730                442  AIKTLNVTD---PTPTQLQAFKNE-VAVLRKTRHVNVLLFMGCMSK------PK--   485
RAF-Acropora-millepora/1-729                    439  AIKTLNVSE---PSPQQLQGFKNE-VAVLRKTRHVNILLFMGCMAK------PE--   482
BRAF-Homo-sapiens/1-766                         481  AVKMLNVTA---PTPQQLQAFKNE-VGVLRKTRHVNILLFMGYSTK------PQ--   524
BRAF-Danio-rerio/1-777                          491  AVKMLNVTA---PTPQQLQAFKNE-VGVLRKTRHVNILLFMGYTTK------PQ--   534
BRAF-Eucidaris-tribuloides-partial/1-725        433  AVKMLNVTD---PTPSQLQAFKNE-VAVLRKTRHANVLLFMGCASK------PQ--   476
BRAF-Strongylocentrotus-purpuratus/1-803        517  AVKRLNVKD---PTPSQLQAFKNE-VAVLRKTRHANILLFMGCTSK------PQ--   560
BRAF-Paracentrotus-lividus-RNAseq/1-746         460  AVKRLNVKD---PTPSQLQAFKNE-VAVLRKTRHANILLFMGCTSK------PQ--   503
BRAF-Paracentrotus-lividus-contigEST/1-716      430  AVKRLNVKD---PTPSQLQAFKNE-VAVLRKTRHANILLFMGCTSK------PQ--   473
BRAF-Saccoglossus-kowaleskii/1-733              445  AVKKLNVTD---PTPSQLQAFKNE-VAVLRKTRHANILLFMGCTSK------PE--   488
RAF-Parastichopus-parvimensis/1-747             457  AIKKLNVKD---PTPSQLQAFKNE-VAVLRKTRHANILLFMGCTSK------PE--   500
BRAF-Branchiostoma-floridae/1-831               542  AVKKLNVKD---PSPAQLQAFRNE-VAVLRKTRHVNILLFMGCVSK------NQ--   585
BRAF-Trichoplax-adherens-partial/1-325           36  AIKKLNVTD---PTPSQLQAFKNE-VTVLRKTRHVNVLLFMGVMSK------PF--    79
BRAF-Patiria-miniata-partial/1-819              531  AVKRLNVTD---PTASQLQAFKNE-VAVLRKTRHANILLFMGCTSK------PQ--   574
KSR1-Caenorhabditis-elegans/1-771               501  AVKVYTMEHISDA-SKKAEEFKLE-VSAYKNTRHDNIALFLGYFMS------DG--   546
KSR2-Caenorhabditis-elegans/1-550               272  AVRFVNMNYLSQE-DRRADVFATEIVSAYKNSRHDHIALFYGYVSD------PVTN   320
KSR1-Amphimedon-queenslandica/1-1246            963  AVKRFVLPN---ATPKQINKFREE-VAVLKKIRHENLALFMGVCLT------TP--  1006
KSR1-Patiria-miniata/1-787                           ----------------------------------------------------
KSR1-Anneissia-japonica/1-885                   618  AVRKLDIDS---NNEEERQAFKKE-VLQLKRTRHDYLVLFMGTCKM------AD--   661
KSR1-Parastichopus-parvimensis/1-879            611  AIKIINIDE---EDKEQLKAFRRE-VQQFKKTRHENVVLFTGACMH------LP--   654
KSR1-Eucidaris-tribuloides/1-873                605  AIKMIDIDF---NDEKQLQAFKGE-VSIFRKTRHDYVVLFMGACMR------PP--   648
KSR1-Strongylocentrotus-purpuratus/1-869        601  AIKMMDIDT---DDEKQLQAFKRE-VSYFRKTRHDFVVLFMGACMR------PP--   644
KSR1-Paracentrotus-lividus/1-848                580  AIKMMDIDT---DDEKQLQAFKRE-VSFFRMTRHDFVVLFMGACMR------PP--   623
KSR1-Trichoplax-adherens/1-966                  705  AIKFVNVSR---LTEEAIESFKLE-VATFRKTRHDNLVLFMGACMK------PP--   748
KSR1-Schmidtea-mediterranea/1-954               681  AVKIMNIDPQ-DENDACMAAFKSE-VGALHKTRHENLVLFMGACME------PP--   726
KSR1-Priapulus-caudatus-partial/1-248                ----------------------------------------------------
KSR1-Clytia-hemispherica/1-881                  606  ALKVLYLEN---PTTEEKNDFKYK-VQVLRRTRHENLVLFMGACME------PA--   649
KSR1-Nematostella-vectensis-partial/1-917       650  AVKMIEIDN---PTEEQLNAFKFQ-VTTFRKTRHDNTVLFMGACMD------PP--   693
KSR1-Acropora-millepora/1-957                   689  AVKIIEIEN---PTEEQLNAFKFQ-VGTFRKTRHENVVLFMGACMD------PP--   732
KSR1-Drosophila-melanogaster/1-966              703  AVKLLNEDYL--QDEHMLETFRSE-VANFKNTRHENLVLFMGACMN------PP--   747
KSR1-Crassostrea-gigas/1-859                    589  AIKMLHMDPDSDNNQAQLSAFKLE-VAMLKNTRHENLLLFMGACMK------PP--   635
KSR1-Saccoglossus-kowalevskii/1-898             629  AIKQFNIDT---DNELQLHSFKQE-VQTFRKTRHENVVLFMGACMN------PP--   672
KSR1-Branchiostoma-floridae/1-865               599  AIKVFNIET---DNDLQLASFKTE-VSMFRKTRHENLVLFMGACMK------LP--   642
KSR1-Capitella-teleta/1-536                     270  AIKMLTVDSQQTDQTSQLQAFKRE-IAMLRKTRHENLVLFMGACMN------PM--   316
KSR1-Rhipicephalus-sanguineus/1-864             597  AVKMLNMDHI--DDRKTLETFKQE-VATFRKTRHENLVLFMGACMK------PP--   641
KSR1-Limulus-polyphemus/1-603                   336  AIKMLNMDDM--DDKKTLETFKQE-VANFRKTRHENLVLFMGACMK------PP--   380
KSR3-Ramazzottius-variornatus-partial/1-451     177  LIYKHN-------AENSVEDFLAD-VENMSKIRHENILLFLGATVD------NE--   216
KSR2-Homo-sapiens/1-950                         690  AIRLIDIER---DNEDQLKAFKRE-VMAYRQTRHENVVLFMGACMS------PP--   733
KSR2-Danio-rerio/1-966                          703  AIRLIDIER---DNEEQLKAFKRE-VMAYRNTRHENVVLFMGACMR------PP--   746
KSR1-Homo-sapiens/1-923                         637  AIRLLEMDG---HNQDHLKLFKKE-VMNYRQTRHENVVLFMGACMN------PP--   680
KSR1-Danio-rerio/1-898                          636  AIRLLEIDG---NNQDHLKLFKKE-VMNYRQTRHENVVLFMGACMA------PP--   679
KSR3-Clytia-hemispherica/1-517                  227  MVYMQA--N---HDENKLDEFLDE-VSLLGLIRHENVVLFMGASIE------PP--   268
KSR3-Schmidtea-mediterranea/1-666               401  SVFMFE--N---LDHDERSRFWEN-VSNLTMIRHENIVLFMGASIE------PP--   442
KSR3-Rhipicephalus-sanguineus/1-675             409  LIYTFR--H---TKEQDVTRFWDE-VGKLSMIRHENIALFMGACAE------PP--   450
KSR3-Limulus-polyphemus/1-686                   420  LIYTVR--Q---QKEAEFRQFLDD-VRQLSMIRHENVVLFMGACIE------PP--   461
KSR3-Limulus-polyphemus-isoformB/1-671          409  LIYTFR--Q---QRQTELCQFLDD-VTQLSMIRHENVVLFMGACID------PP--   450
KSR3-Lingula-anatina/1-626                      368  IIHKYD--P---S---VEDVFWTK-ISMLRMIRHENIALFMGACAE------PP--   406
KSR3-Crassostrea-gigas/1-585                    322  NIHDFH--A---E--SDTSDFWNK-VRKLCRIRHENLVLFMGACLE------EC--   361
KSR3-Nematostella-vectensis/1-640               364  MIHTVK--K---SSKIDLEEFLKE-VSLLSMIRHENVVLFMGACLE------PP--   405
KSR3-Acropora-millepora/1-634                   365  MIHTRR--R---STKKDVEEFLKE-VSVLSMIRHENVVLFMGACLE------PP--   406
KSR3-Priapulus-caudatus/1-533                   269  MVHAFQ--S---SSQLDVDRFWEE-VKVLSMIRHENVALFMGACVE------PP--   310
KSR3-Saccoglossus-kowaleskii/1-591              326  VIHTYH--S---S--RDVNEFLEE-VAILSRIRHENIELFMGVSTE------PS--   365
KSR3-Ptychodera-flava/1-629                     359  IIHTYQ--A---T--RNFNEFLEE-VAVLSRIRHENIELFMGVSLE------PT--   398
KSR3-Trichoplax-adherens/1-510                  254  MIHTRE--N---LNKKEISDLWQE-VSILSMIRHDNISLFMGACFE------PP--   295
KSR3-Capitella-teleta/1-642                     406  MIHTYQ--D---RKEEEVTEFLGE-VSRMSILRHENIALFMGACLE------EP--   447
KSR3-Eucidaris-tribuloides/1-545                345  LIHSRH--N-----VAHLDEFLDE-VSMLSMIRHENIALFVGACVE------PN--   384
KSR3-Strongylocentrotus-purpuratus/1-602        341  LIQTRY--S---K-STDINDFLDE-VAMLSKIRHENIALFMGACVE------PG--   381
KSR3-Paracentrotus-lividus-RNAseq/1-600         339  LIQTRN--A---K-STDINDFLDE-VAMLSKIRHENIALFMGACVE------PG--   379
KSR3-Notospermus-geniculatus/1-691              426  MIHAYE--G---IEEDQVQAFWEE-IAKLSMIRHENIALFMGACIE------PP--   467
KSR3-Aplysia-californica/1-405                  144  IVHVFG--E---LRQKDKDAFWAN-LGKLCRIRHENIILFMAACTS------QP--   185
KSR3-Amphiura-filiformis-partial/1-642          416  LIHTRN--D---V--HDLEEFLEE-VSLLSMIRHENIALFMGACID------RP--   455
KSR3-Platynereis-dumerilii/1-704                439  MIHIYK--G---IEEEEVARFWSE-VSKLSMIRHENVALFMGACIE------HP--   480
KSR3-Patiria-miniata/1-779                      524  LIHTRL--H---V--EDVDAFLEE-VSVLSMIRHENIALFMGACID------PP--   563
KSR3-Anneissia-japonica/1-590                   328  IIHTRD--V---Q---EEEDFLEE-VSMLSMIRHENIALFMGACID------AP--   366
KSR3-Parastichopus-parvimensis-partial/1-330     68  LIHTRT--N---A--ENLEHFLDE-VAMLSMVRHENVALFMGACID------EP--   107
```

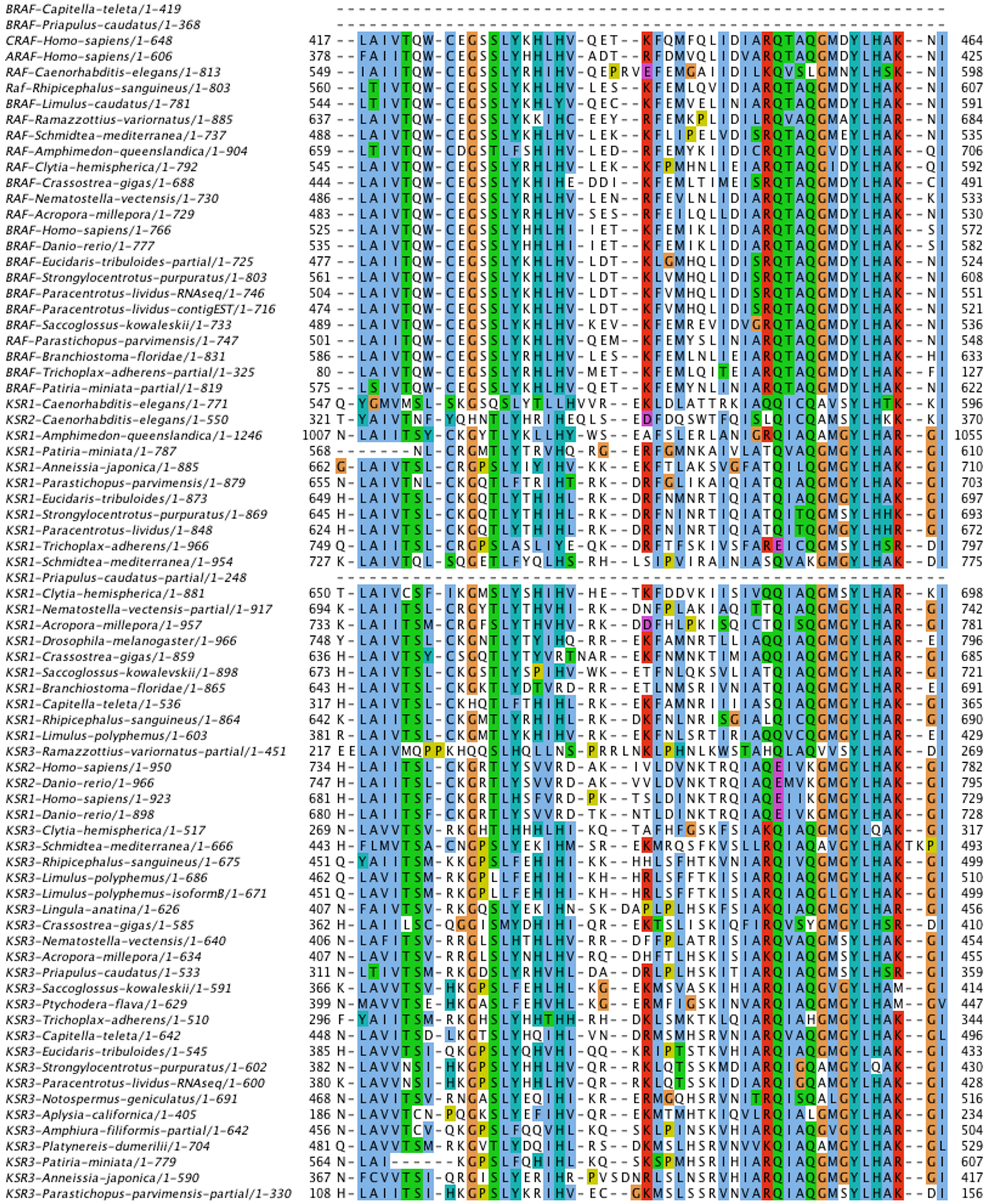
BRAF-Capitella-teleta/1-419
BRAF-Priapulus-caudatus/1-368
CRAF-Homo-sapiens/1-648 417 464
ARAF-Homo-sapiens/1-606 378 425
RAF-Caenorhabditis-elegans/1-813 549 598
Raf-Rhipicephalus-sanguineus/1-803 560 607
BRAF-Limulus-caudatus/1-781 544 591
RAF-Ramazzottius-variornatus/1-885 637 684
RAF-Schmidtea-mediterranea/1-737 488 535
RAF-Amphimedon-queenslandica/1-904 659 706
RAF-Clytia-hemispherica/1-792 545 592
BRAF-Crassostrea-gigas/1-688 444 491
RAF-Nematostella-vectensis/1-730 486 533
RAF-Acropora-millepora/1-729 483 530
BRAF-Homo-sapiens/1-766 525 572
BRAF-Danio-rerio/1-777 535 582
BRAF-Eucidaris-tribuloides-partial/1-725 477 524
BRAF-Strongylocentrotus-purpuratus/1-803 561 608
BRAF-Paracentrotus-lividus-RNAseq/1-746 504 551
BRAF-Paracentrotus-lividus-contigEST/1-716 474 521
BRAF-Saccoglossus-kowaleskii/1-733 489 536
RAF-Parastichopus-parvimensis/1-747 501 548
BRAF-Branchiostoma-floridae/1-831 586 633
BRAF-Trichoplax-adherens-partial/1-325 80 127
BRAF-Patiria-miniata-partial/1-819 575 622
KSR1-Caenorhabditis-elegans/1-771 547 596
KSR2-Caenorhabditis-elegans/1-550 321 370
KSR1-Amphimedon-queenslandica/1-1246 1007 1055
KSR1-Patiria-miniata/1-787 568 610
KSR1-Anneissia-japonica/1-885 662 710
KSR1-Parastichopus-parvimensis/1-879 655 703
KSR1-Eucidaris-tribuloides/1-873 649 697
KSR1-Strongylocentrotus-purpuratus/1-869 645 693
KSR1-Paracentrotus-lividus/1-848 624 672
KSR1-Trichoplax-adherens/1-966 749 797
KSR1-Schmidtea-mediterranea/1-954 727 775
KSR1-Priapulus-caudatus-partial/1-248
KSR1-Clytia-hemispherica/1-881 650 698
KSR1-Nematostella-vectensis-partial/1-917 694 742
KSR1-Acropora-millepora/1-957 733 781
KSR1-Drosophila-melanogaster/1-966 748 796
KSR1-Crassostrea-gigas/1-859 636 685
KSR1-Saccoglossus-kowalevskii/1-898 673 721
KSR1-Branchiostoma-floridae/1-865 643 691
KSR1-Capitella-teleta/1-536 317 365
KSR1-Rhipicephalus-sanguineus/1-864 642 690
KSR1-Limulus-polyphemus/1-603 381 429
KSR3-Ramazzottius-variornatus-partial/1-451 217 269
KSR2-Homo-sapiens/1-950 734 782
KSR2-Danio-rerio/1-966 747 795
KSR1-Homo-sapiens/1-923 681 729
KSR1-Danio-rerio/1-898 680 728
KSR3-Clytia-hemispherica/1-517 269 317
KSR3-Schmidtea-mediterranea/1-666 443 493
KSR3-Rhipicephalus-sanguineus/1-675 451 499
KSR3-Limulus-polyphemus/1-686 462 510
KSR3-Limulus-polyphemus-isoformB/1-671 451 499
KSR3-Lingula-anatina/1-626 407 456
KSR3-Crassostrea-gigas/1-585 362 410
KSR3-Nematostella-vectensis/1-640 406 454
KSR3-Acropora-millepora/1-634 407 455
KSR3-Priapulus-caudatus/1-533 311 359
KSR3-Saccoglossus-kowaleskii/1-591 366 414
KSR3-Ptychodera-flava/1-629 399 447
KSR3-Trichoplax-adherens/1-510 296 344
KSR3-Capitella-teleta/1-642 448 496
KSR3-Eucidaris-tribuloides/1-545 385 433
KSR3-Strongylocentrotus-purpuratus/1-602 382 430
KSR3-Paracentrotus-lividus-RNAseq/1-600 380 428
KSR3-Notospermus-geniculatus/1-691 468 516
KSR3-Aplysia-californica/1-405 186 234
KSR3-Amphiura-filiformis-partial/1-642 456 504
KSR3-Platynereis-dumerilii/1-704 481 529
KSR3-Patiria-miniata/1-779 564 607
KSR3-Anneissia-japonica/1-590 367 417
KSR3-Parastichopus-parvimensis-partial/1-330 108 156

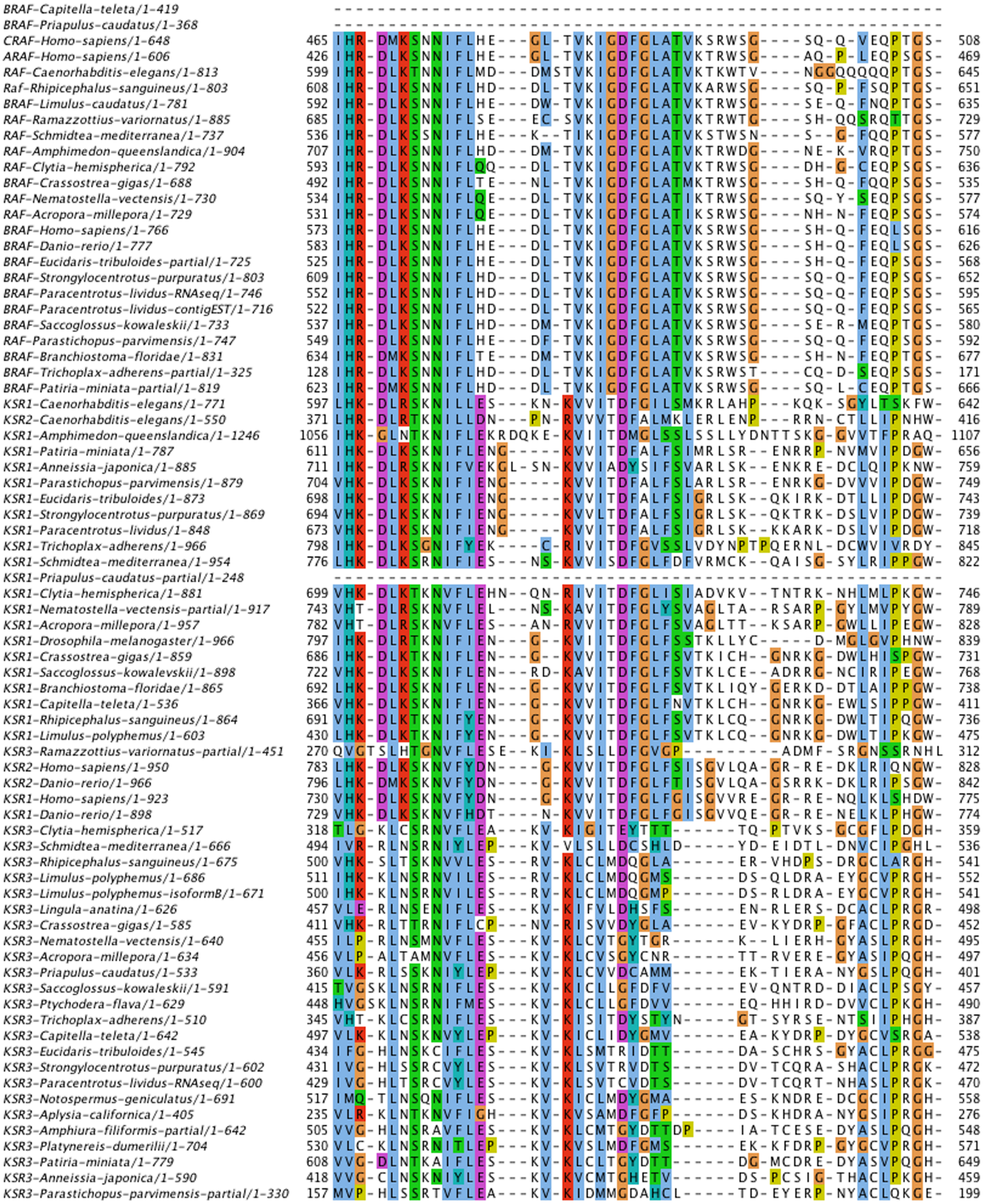
BRAF-Capitella-teleta/1-419
BRAF-Priapulus-caudatus/1-368
CRAF-Homo-sapiens/1-648
ARAF-Homo-sapiens/1-606
RAF-Caenorhabditis-elegans/1-813
Raf-Rhipicephalus-sanguineus/1-803
BRAF-Limulus-caudatus/1-781
RAF-Ramazzottius-variornatus/1-885
RAF-Schmidtea-mediterranea/1-737
RAF-Amphimedon-queenslandica/1-904
RAF-Clytia-hemispherica/1-792
BRAF-Crassostrea-gigas/1-688
RAF-Nematostella-vectensis/1-730
RAF-Acropora-millepora/1-729
BRAF-Homo-sapiens/1-766
BRAF-Danio-rerio/1-777
BRAF-Eucidaris-tribuloides-partial/1-725
BRAF-Strongylocentrotus-purpuratus/1-803
BRAF-Paracentrotus-lividus-RNAseq/1-746
BRAF-Paracentrotus-lividus-contigEST/1-716
BRAF-Saccoglossus-kowaleskii/1-733
RAF-Parastichopus-parvimensis/1-747
BRAF-Branchiostoma-floridae/1-831
BRAF-Trichoplax-adherens-partial/1-325
BRAF-Patiria-miniata-partial/1-819
KSR1-Caenorhabditis-elegans/1-771
KSR2-Caenorhabditis-elegans/1-550
KSR1-Amphimedon-queenslandica/1-1246
KSR1-Patiria-miniata/1-787
KSR1-Anneissia-japonica/1-885
KSR1-Parastichopus-parvimensis/1-879
KSR1-Eucidaris-tribuloides/1-873
KSR1-Strongylocentrotus-purpuratus/1-869
KSR1-Paracentrotus-lividus/1-848
KSR1-Trichoplax-adherens/1-966
KSR1-Schmidtea-mediterranea/1-954
KSR1-Priapulus-caudatus-partial/1-248
KSR1-Clytia-hemispherica/1-881
KSR1-Nematostella-vectensis-partial/1-917
KSR1-Acropora-millepora/1-957
KSR1-Drosophila-melanogaster/1-966
KSR1-Crassostrea-gigas/1-859
KSR1-Saccoglossus-kowalevskii/1-898
KSR1-Branchiostoma-floridae/1-865
KSR1-Capitella-teleta/1-536
KSR1-Rhipicephalus-sanguineus/1-864
KSR1-Limulus-polyphemus/1-603
KSR3-Ramazzottius-variornatus-partial/1-451
KSR2-Homo-sapiens/1-950
KSR2-Danio-rerio/1-966
KSR1-Homo-sapiens/1-923
KSR1-Danio-rerio/1-898
KSR3-Clytia-hemispherica/1-517
KSR3-Schmidtea-mediterranea/1-666
KSR3-Rhipicephalus-sanguineus/1-675
KSR3-Limulus-polyphemus/1-686
KSR3-Limulus-polyphemus-isoformB/1-671
KSR3-Lingula-anatina/1-626
KSR3-Crassostrea-gigas/1-585
KSR3-Nematostella-vectensis/1-640
KSR3-Acropora-millepora/1-634
KSR3-Priapulus-caudatus/1-533
KSR3-Saccoglossus-kowaleskii/1-591
KSR3-Ptychodera-flava/1-629
KSR3-Trichoplax-adherens/1-510
KSR3-Capitella-teleta/1-642
KSR3-Eucidaris-tribuloides/1-545
KSR3-Strongylocentrotus-purpuratus/1-602
KSR3-Paracentrotus-lividus-RNAseq/1-600
KSR3-Notospermus-geniculatus/1-691
KSR3-Aplysia-californica/1-405
KSR3-Amphiura-filiformis-partial/1-642
KSR3-Platynereis-dumerilii/1-704
KSR3-Patiria-miniata/1-779
KSR3-Anneissia-japonica/1-590
KSR3-Parastichopus-parvimensis-partial/1-330

BRAF-Capitella-teleta/1-419		--	
BRAF-Priapulus-caudatus/1-368		--	
CRAF-Homo-sapiens/1-648	509	VLWMAPEVIRMQ--DN---------NPF--SFQSDVYSYGIVLY--ELMTGELPYS	549
ARAF-Homo-sapiens/1-606	470	VLWMAAEVIRMQ--DP---------NPY--SFQSDVYAYGVVLY--ELMTGSLPYS	510
RAF-Caenorhabditis-elegans/1-813	646	ILWMAPEVIRMQ--DD---------NPY--TPQSDVYSFGICMY--EILSSHLPYS	686
Raf-Rhipicephalus-sanguineus/1-803	652	ILWMAPEVIRMK--DP---------NPY--SFQSDVYAFGIVLY--ELITGQLPYS	692
BRAF-Limulus-caudatus/1-781	636	ILWMAPEVIRMK--DS---------HPY--SFQSDVYAFGIVLY--ELIAGQLPYA	676
RAF-Ramazzottius-variornatus/1-885	730	ILWMAPEVIRMDKDDS---------HPF--SSKSDVYSFGVVLY--ELLSGKLPYE	772
RAF-Schmidtea-mediterranea/1-737	578	ILWMAPEVIRMD--SQ---------NPY--TTLSDVYAYGVVLY--ELTSGQLPYR	618
RAF-Amphimedon-queenslandica/1-904	751	ILWMAPEVIRME--EP---------DPY--TFYCDVYSFGVVVY--ELISGQLPYP	791
RAF-Clytia-hemispherica/1-792	637	ILWMAPEVIKMQ--DP---------NPY--TFFSDAYAFGICIY--ELITGSLPYC	677
BRAF-Crassostrea-gigas/1-688	536	ILWMAPEVIRMK--EA---------NPY--TNQSDVYAFGIVLY--ELMTQSLPYS	576
RAF-Nematostella-vectensis/1-730	578	ILWMAAEVIRML--DP---------NPY--SFHSDVYAFGIVLY--ELLSSSLPYA	618
RAF-Acropora-millepora/1-729	575	ILWMAPEVVRMQ--DP---------NPY--SFHSDIYAYGIVLY--ELQTGSLPYP	615
BRAF-Homo-sapiens/1-766	617	ILWMAPEVIRMQ--DK---------NPY--SFQSDVYAFGIVLY--ELMTGQLPYS	657
BRAF-Danio-rerio/1-777	627	ILWMAPEVIRLQ--DK---------NPY--SFQSDVYAFGIVLY--ELMSGALPYS	667
BRAF-Eucidaris-tribuloides-partial/1-725	569	ILWMAPEVIQMR--DP---------NPY--SFQSDVYAFGIVLY--ELITGELPYQ	609
BRAF-Strongylocentrotus-purpuratus/1-803	653	ILWMAPEVIRMR--DT---------NPY--SFQSDVYAFGVVLF--ELVTQSLPYQ	693
BRAF-Paracentrotus-lividus-RNAseq/1-746	596	ILWMAPEVIRMR--DK---------NPY--SLESDVYAFGVVLF--ELVTQSLPYQ	636
BRAF-Paracentrotus-lividus-contigEST/1-716	566	ILWMAPEVIRMR--DK---------NPY--SLESDVYAFGVVLF--ELVTQSLPYQ	606
BRAF-Saccoglossus-kowaleskii/1-733	581	ILWMAPEVIRMQ--DR---------NPY--TPQSDVYAFGICLY--ELATGQLPYS	621
RAF-Parastichopus-parvimensis/1-747	593	ILWMAPEVIRMR--DP---------NPY--SFQSDVFAFGIVLY--ELMTGSLPYS	633
BRAF-Branchiostoma-floridae/1-831	678	ILWMAPEVIRMQ--DP---------NPY--SFQSDVYGFGVVLF--ELVTGQLPYA	718
BRAF-Trichoplax-adherens-partial/1-325	172	ILWMAPEVMRMQ--DV---------TPY--TVRSDIYSFGMVLL--ELTTGELPYS	212
BRAF-Patiria-miniata-partial/1-819	667	ILWMAPEVIRMR--DP---------NPY--SFQSDVYAFGVVLF--ELVSGQLPYS	707
KSR1-Caenorhabditis-elegans/1-771	643	TNYIAPELAMAMRTEYD--EYECDDFPF--SENSDVYAFGCVWF--EMLTGALPYA	692
KSR2-Caenorhabditis-elegans/1-550	417	IDYLSPEIAGNLMIDWRGDVLFQHELPF--SQESDVYSFGTIFF--ELLLRRMPTG	468
KSR1-Amphimedon-queenslandica/1-1246	1108	LYSLAPEIIRKISPTPVVQ-IDQEPYSY--NYATDVYAFGTILY--ELITKRFPFS	1158
KSR1-Patiria-miniata/1-787	657	LCYLAPELITALRVDS--SGSSDRELPF--STTSDIYSFGTIWY--ELLAGCLPFH	706
KSR1-Anneissia-japonica/1-885	760	LCYLAPELIKALKVK-----DIDGELPF--SKASDVYAFGTVWY--ELLAKEFPMA	806
KSR1-Parastichopus-parvimensis/1-879	750	LCYLSPEVITSLAVVP---EKVAMDLPF--TEKSDVYAFGTVWF--ELLTGEWPFH	798
KSR1-Eucidaris-tribuloides/1-873	744	LCYLAPELITSLRINM--DGNTDMELPF--SDKSDIYAFGTVWY--ELLAGEWPFH	793
KSR1-Strongylocentrotus-purpuratus/1-869	740	LCYLAPELITSLKINT--DGTQDMELPF--SEKSDVYSFGTIWY--ELLAGEWPFH	789
KSR1-Paracentrotus-lividus/1-848	719	LCYLAPELMTSLKINT--DGNQDMELPF--SEKSDVHSFGTIWY--ELLAGEWPFH	768
KSR1-Trichoplax-adherens/1-966	846	AVYFAPEIIKKMDLNL----VSFGLSAF--TKASDVFAFGTVLY--EMMVGHWPFS	893
KSR1-Schmidtea-mediterranea/1-954	823	LCYLAPELIRKLNLNSIFSNNSSEDLPF--TKSSDIYAFGTIWY--EILNSKWPFE	874
KSR1-Priapulus-caudatus-partial/1-248		--	
KSR1-Clytia-hemispherica/1-881	747	LCYQAPEIIRHLSAHD----PGCEVLYY--TRESDIYAFGTIWF--ELLAGDWPFA	794
KSR1-Nematostella-vectensis-partial/1-917	790	LYYLAPEVIRTLTPFQ----DAPESKQF--TTKTDVYSFGTVWY--EMIAGGWPWK	837
KSR1-Acropora-millepora/1-957	829	LPYLAPEVIRSLTTQQ----DAPDSSQF--TMATDMYAFGTVWY--EMCIGAWPFR	876
KSR1-Drosophila-melanogaster/1-966	840	LCYLAPELIRALQPEK----PRGECLEF--TPYSDVYSFGTVWY--ELICGEFTFK	887
KSR1-Crassostrea-gigas/1-859	732	LCYLSPEVIRCLHSGR-----SKVDLPF--SEKSDVYAFGTIWY--ELLVGDWPFR	778
KSR1-Saccoglossus-kowalevskii/1-898	769	LCYLAPELITSLKITN---LGLDNELPF--SKSSDVYSFGTVWY--ELLAGEWTFN	817
KSR1-Branchiostoma-floridae/1-865	739	LPYLAPEVITSLRATS---QYTDSDLPF--SQASDVYAFGTVWY--ELLVGDLPFK	787
KSR1-Capitella-teleta/1-536	412	LCYLAPEVIRSLQAFN------HMDLPF--SKASDVYAFGTVWY--ELLCGEWPFH	457
KSR1-Rhipicephalus-sanguineus/1-864	737	LCYLAPEVVRCLRADN---QHDADDLPF--ATASDIYAFGTVWY--ELLCGEWPFR	785
KSR1-Limulus-polyphemus/1-603	476	LCYLAPEVIRSLKAGN---QPDDTDLPF--STASDVYAFGTVWY--ELLYGEWPFH	524
KSR3-Ramazzottius-variornatus-partial/1-451	313	SSFLAPELLCTASDVS---ALGMQQLKAQCSEATDSFAFGSILY--ELFTEQKPFR	363
KSR2-Homo-sapiens/1-950	829	LCHLAPEIIRQLSP-----DTEEDKLPF--SKHSDVFALGTIWY--ELHAREWPFK	875
KSR2-Danio-rerio/1-966	843	LCHLAPELIRQLSP-----ETAENQLPF--SKQSDVFAFGTIWY--ELHAREWPFK	889
KSR1-Homo-sapiens/1-923	776	LCYLAPEIVREMTP-----GKDEDQLPF--SKAADVYAFGTVWY--ELQARDWPLK	822
KSR1-Danio-rerio/1-898	775	ICYLAPEIVRKMSP-----GNNEDRLPF--SNAADVYAFGTIWY--ELQARDWPIT	821
KSR3-Clytia-hemispherica/1-517	360	LAYISPELMRTLKLCG---IYYLTDKPD--TPKADVYAYGTLLY--EIFLGRHPYK	408
KSR3-Schmidtea-mediterranea/1-666	537	VWYAAPEILEMGGKDL---PKIIPEKYF--VQTTDVYSFGMLTY--EIHMGRYPST	585
KSR3-Rhipicephalus-sanguineus/1-675	542	LTYLAPELMRQLKVEP---PNVFSDCEP--SQESDLYAFGTILY--ELIAEAFPFN	590
KSR3-Limulus-polyphemus/1-686	553	LAYFSPEILQNLQIEP---PKVYSTMPY--TQEADIFAFGSILY--ELFAEEFPFS	601
KSR3-Limulus-polyphemus-isoformB/1-671	542	LAYLSPELMRNLQIEP---PKIYSTVSN--TKETDIFAFGSIVY--ELLAEYFPFS	590
KSR3-Lingula-anatina/1-626	499	ATYFAPEILRTMRIEN---QQVKLEQ-N--TKESDTFAFGTVLY--ELFNGRFPHD	546
KSR3-Crassostrea-gigas/1-585	453	LTYIAPEVLCSMSVVP---PMLVPSVPF--TNETDVYAFGTVVY--ELLTGHYPYY	501
KSR3-Nematostella-vectensis/1-640	496	LTYVPPEHMRKISWSE---NELELVQCCNSSVKTNVYVFGTLLY--EIMTGRWPFS	546
KSR3-Acropora-millepora/1-634	498	LTYLAPELMRTLTRKG---CVLSPKECN--TEWSNVYAFSTILY--EIFSGKWPFS	546
KSR3-Priapulus-caudatus/1-533	402	ITYFSPEIMCSLKVEP---PHVTSSGPY--TMASDVYAFGTVLY--ELMHGEYPFR	450
KSR3-Saccoglossus-kowaleskii/1-591	458	LSYMAPELLRTVRVES---SELVTDMQY--NTTTDIFAFGTILY--ELFSGSWPFD	506
KSR3-Ptychodera-flava/1-629	491	LTYMAPEIMRTLQIEN---GDIINDTPY--NTMTDSFAFGTVMY--ELFSGSWPFH	539
KSR3-Trichoplax-adherens/1-510	388	LTYISPELMRTMIVDP---PNFNMSMPY--TYYSDMYAFGTILY--ELVAFEWPFA	436
KSR3-Capitella-teleta/1-642	539	LLYVAPELLRSLRING---SKIEPTAPF--SKATDVYAYGTLLF--EVLSGCFPMR	587
KSR3-Eucidaris-tribuloides/1-545	476	LNYLAPEMLVASRVVP---PELVIEACQ--TQQPTSTPLEQHCLKSSLDIGRLRML	526
KSR3-Strongylocentrotus-purpuratus/1-602	473	LQYLAPELLAKARVVP---PEFITGNEQ--THSTDVYAFGTILF--EIIAGRWPLQ	521
KSR3-Paracentrotus-lividus-RNAseq/1-600	471	LSYLAPELLASSRVEP---PELITSAAQ--THSTDVFAFGTILF--EIIAGRWPLQ	519
KSR3-Notospermus-geniculatus/1-691	559	LTYIAPETLTTVYIDP---PRLVSVHPY--TKATDVFAFGTVMY--ELMSGQFPFH	607
KSR3-Aplysia-californica/1-405	277	LTYIAPEIMCTIRVLP---PRLMPSATY--SPETDVFAFGTVMY--EVFACRFPFH	325
KSR3-Amphiura-filiformis-partial/1-642	549	LTYIAPEILKKMTALP---PSLVTEENY--SIESDVFAFGTILY--ELLSGSWPFA	597
KSR3-Platynereis-dumerilii/1-704	572	LTYTAPEMLRTLRVES---PRLTSKTQH--TTQTDVYAFGTLLF--ELMASRYPFR	620
KSR3-Patiria-miniata/1-779	650	LTYIAPEILRSIRVQP---PYVIEAEPH--TMESDVYAFGTVLY--ELLTSQWPFA	698
KSR3-Anneissia-japonica/1-590	460	ITYLAPELLRRATVTP---PTLTIDSIV--TQECDIYAFGTVLY--ELVSQKWPFR	508
KSR3-Parastichopus-parvimensis-partial/1-330	200	IQYLPPETLRGAAVHP---PNLKYNNAK--TPASDVFAFGTILF--ELLAGRFPFQ	248

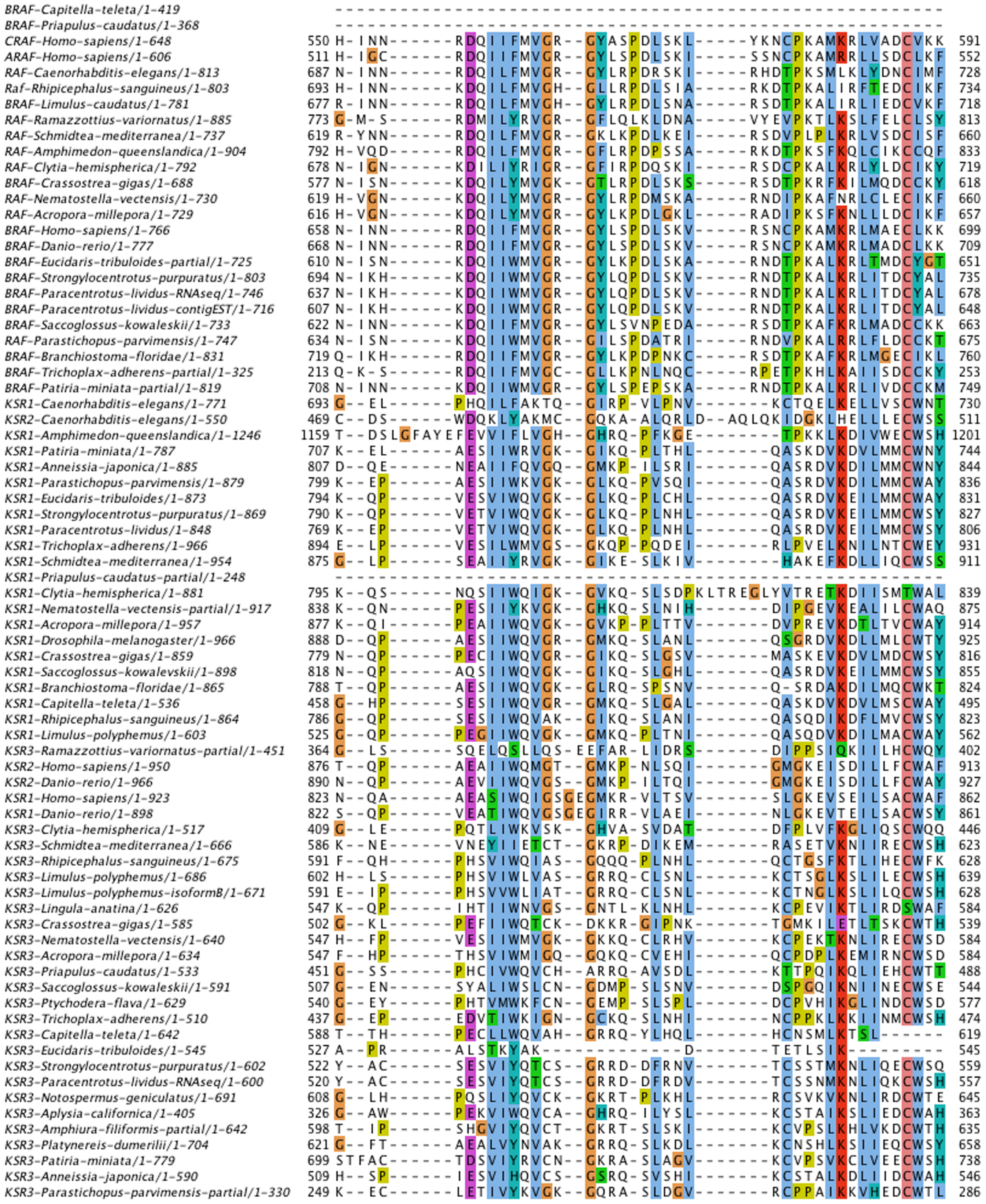
BRAF-Capitella-teleta/1-419
BRAF-Priapulus-caudatus/1-368
CRAF-Homo-sapiens/1-648
ARAF-Homo-sapiens/1-606
RAF-Caenorhabditis-elegans/1-813
Raf-Rhipicephalus-sanguineus/1-803
BRAF-Limulus-caudatus/1-781
RAF-Ramazzottius-variornatus/1-885
RAF-Schmidtea-mediterranea/1-737
RAF-Amphimedon-queenslandica/1-904
RAF-Clytia-hemispherica/1-792
BRAF-Crassostrea-gigas/1-688
RAF-Nematostella-vectensis/1-730
RAF-Acropora-millepora/1-729
BRAF-Homo-sapiens/1-766
BRAF-Danio-rerio/1-777
BRAF-Eucidaris-tribuloides-partial/1-725
BRAF-Strongylocentrotus-purpuratus/1-803
BRAF-Paracentrotus-lividus-RNAseq/1-746
BRAF-Paracentrotus-lividus-contigEST/1-716
BRAF-Saccoglossus-kowaleskii/1-733
RAF-Parastichopus-parvimensis/1-747
BRAF-Branchiostoma-floridae/1-831
BRAF-Trichoplax-adherens-partial/1-325
BRAF-Patiria-miniata-partial/1-819
KSR1-Caenorhabditis-elegans/1-771
KSR2-Caenorhabditis-elegans/1-550
KSR1-Amphimedon-queenslandica/1-1246
KSR1-Patiria-miniata/1-787
KSR1-Anneissia-japonica/1-885
KSR1-Parastichopus-parvimensis/1-879
KSR1-Eucidaris-tribuloides/1-873
KSR1-Strongylocentrotus-purpuratus/1-869
KSR1-Paracentrotus-lividus/1-848
KSR1-Trichoplax-adherens/1-966
KSR1-Schmidtea-mediterranea/1-954
KSR1-Priapulus-caudatus-partial/1-248
KSR1-Clytia-hemispherica/1-881
KSR1-Nematostella-vectensis-partial/1-917
KSR1-Acropora-millepora/1-957
KSR1-Drosophila-melanogaster/1-966
KSR1-Crassostrea-gigas/1-859
KSR1-Saccoglossus-kowalevskii/1-898
KSR1-Branchiostoma-floridae/1-865
KSR1-Capitella-teleta/1-536
KSR1-Rhipicephalus-sanguineus/1-864
KSR1-Limulus-polyphemus/1-603
KSR3-Ramazzottius-variornatus-partial/1-451
KSR2-Homo-sapiens/1-950
KSR2-Danio-rerio/1-966
KSR1-Homo-sapiens/1-923
KSR1-Danio-rerio/1-898
KSR3-Clytia-hemispherica/1-517
KSR3-Schmidtea-mediterranea/1-666
KSR3-Rhipicephalus-sanguineus/1-675
KSR3-Limulus-polyphemus/1-686
KSR3-Limulus-polyphemus-isoformB/1-671
KSR3-Lingula-anatina/1-626
KSR3-Crassostrea-gigas/1-585
KSR3-Nematostella-vectensis/1-640
KSR3-Acropora-millepora/1-634
KSR3-Priapulus-caudatus/1-533
KSR3-Saccoglossus-kowaleskii/1-591
KSR3-Ptychodera-flava/1-629
KSR3-Trichoplax-adherens/1-510
KSR3-Capitella-teleta/1-642
KSR3-Eucidaris-tribuloides/1-545
KSR3-Strongylocentrotus-purpuratus/1-602
KSR3-Paracentrotus-lividus-RNAseq/1-600
KSR3-Notospermus-geniculatus/1-691
KSR3-Aplysia-californica/1-405
KSR3-Amphiura-filiformis-partial/1-642
KSR3-Platynereis-dumerilii/1-704
KSR3-Patiria-miniata/1-779
KSR3-Anneissia-japonica/1-590
KSR3-Parastichopus-parvimensis-partial/1-330

```
BRAF-Capitella-teleta/1-419                             --------------------------------------------------------
BRAF-Priapulus-caudatus/1-368                           --------------------------------------------------------
CRAF-Homo-sapiens/1-648                             592 VKEERPLFPQILSSIELLQHSLP--------KINRSASEP-S---LHRAAHTEDIN  635
ARAF-Homo-sapiens/1-606                             553 QREERPLFPQILATIELLQRSLP--------KIERSASEP-S---LHRTQA-DELP  595
RAF-Caenorhabditis-elegans/1-813                    729 DRNERPVFGEVLERLRDI--ILP--------KLTRSQSAPNV---LHLDSQYSVMD  771
Raf-Rhipicephalus-sanguineus/1-803                  735 NRDERPLFRQMLASLESLMRSLP--------KIHQSTSEP-T---LNRTHLQS---  775
BRAF-Limulus-caudatus/1-781                         719 NREERPLFRQILASLELLARSLP--------KIHRSTSEP-T---LNRTHLQS---  759
RAF-Ramazzottius-variornatus/1-885                  814 KREDRPEFTEIHNQLQHAMERIP--------VIWRSNSEP-T---LNRNHWQQ---  854
RAF-Schmidtea-mediterranea/1-737                    661 ISDCRPPFSQIHSKLDTLWRSLP--------KLQRCTSEPSI----SGSHLQR---  701
RAF-Amphimedon-queenslandica/1-904                  834 EKDDRPLFHQILADLESLSDSMP--------KIKRSNSEPTT---LCYAGLSY---  875
RAF-Clytia-hemispherica/1-792                       720 NRDERPLFKQIVNSLETILMALP--------KITRSTSEP-I---LGRAKPST---  760
BRAF-Crassostrea-gigas/1-688                        619 DREQRPLFPQILSVLESLERSLP--------KVHRSSSEP-T---LNL--SQS---  657
RAF-Nematostella-vectensis/1-730                    661 NRDERPLFPQILASLEAIHRALP--------KIQRSTSEPWN---IG-AKLTS---  701
RAF-Acropora-millepora/1-729                        658 NRDERPLFTKVLASLESLIRSMP--------KIHRSASEPCS---LNRAGRMM---  699
BRAF-Homo-sapiens/1-766                             700 KRDERPLFPQILASIELLARSLP--------KIHRSASEP-S---LNRAGFQT---  740
BRAF-Danio-rerio/1-777                              710 KREERPLFPQILASIELLARSLP--------KIHRSASEP-S---LNRAGFQT---  750
BRAF-Eucidaris-tribuloides-partial/1-725            652 -KDERPLFPVVLAALENLARQLP--------KIHRSASEPSS---LNRARLHS---  692
BRAF-Strongylocentrotus-purpuratus/1-803            736 NRDERPLFPVVLAALESLARQLP--------KIHRSASEPSS---LNRARLHS---  777
BRAF-Paracentrotus-lividus-RNAseq/1-746             679 NRDERPLFPVILASLEMLARQLP--------KIHRSASEPSS---LNRARLHS---  720
BRAF-Paracentrotus-lividus-contigEST/1-716          649 NRDERPLFPVILASLEMLARQLP--------KIHRSASEPSS---LNRARLHS---  690
BRAF-Saccoglossus-kowaleskii/1-733                  664 NRDDRPLFPQVLASLEHLARSLP--------KLHRSASEPVS---LNRTRLQS---  705
RAF-Parastichopus-parvimensis/1-747                 676 QRDERPLFPQILAALEGLAKSLP--------KIHKSSSEP-S---MNRARFNS---  716
BRAF-Branchiostoma-floridae/1-831                   761 NRDERPLFPQILASLDSLVRSLP--------KITRSASEP-S---LNRAGFNS---  801
BRAF-Trichoplax-adherens-partial/1-325              254 SRDERPDFAQVLGVLENLAQDIP--------KIHRSMSEP-M---INRPD------  291
BRAF-Patiria-miniata-partial/1-819                  750 NRDERPLFPQILASLEGLARLLP--------KVHRSHSEP-S---MNRARFQT---  790
KSR1-Caenorhabditis-elegans/1-771                   731 APQDRPTLTDINLKLTALPKKPR---------VNRSPSFPVM---MKSYEST----  770
KSR2-Caenorhabditis-elegans/1-550                   512 QPEKRPSFQQIVKRITVQMPRKESNKQKRRSTAHENPLF-----------------  550
KSR1-Amphimedon-queenslandica/1-1246               1202 QPQDRPPFSDLQERLARLCRKHT--------PITRSPSHPIN---LSKSVDNL--- 1243
KSR1-Patiria-miniata/1-787                          745 NPEKRPEFTDIIKTLNRLPKKRG--------GLHRSPSHPLH---LSRSAESV---  786
KSR1-Anneissia-japonica/1-885                       845 TPSARPSFSALTKTLDRLPKR----------RIMRSPSTPLH---LSRSAESV---  884
KSR1-Parastichopus-parvimensis/1-879                837 YKESRPSFSNIKRTLLRLPRKRP--------GLHRSPSHPLH---LSRSAESI---  878
KSR1-Eucidaris-tribuloides/1-873                    832 TPESRPTFPQIKKTIIRLPKK-K--------GLHRSPSHPLH---LSRSAESV---  872
KSR1-Strongylocentrotus-purpuratus/1-869            828 SPDSRPIFQHIKKTILRLPKK-K--------GLHRSPSHPLH---LSRSAESV---  868
KSR1-Paracentrotus-lividus/1-848                    807 SPESRPMFQHIKKTILRLPKK-K--------GLHRSPSHPLH---LSRSAEPV---  847
KSR1-Trichoplax-adherens/1-966                      932 DPEKRIAFTNLLKALN--KLPER--------YLRRSPSCPSNSLT-----------  966
KSR1-Schmidtea-mediterranea/1-954                   912 KADNRPAFTNIVQILE--KLP----------KLQRSPSYPVKSVSVVRSHDNL---  952
KSR1-Priapulus-caudatus-partial/1-248                   --------------------------------------------------------
KSR1-Clytia-hemispherica/1-881                      840 DPENRPQFSALLKAFE--RLPKK--------RLIRSPSQPTY---LTRTANAL---  879
KSR1-Nematostella-vectensis-partial/1-917           876 NPESRPTFSQLLKTIDRLPKK-H--------RLHRSPSQPCT---VGRPAEAL---  916
KSR1-Acropora-millepora/1-957                       915 DPEKRPTFSNLLRTLERLPKQRQ--------RLRRSPSQPCT---VGRGTEAV---  956
KSR1-Drosophila-melanogaster/1-966                  926 EKEHRPQFARLLSLLE--HLPKK--------RLARSPSHPVN---LSRSAESV---  965
KSR1-Crassostrea-gigas/1-859                        817 QPDDRPQFRALLKLVE--KLPKK--------RLVRSPSHPVH---LSRSVDSV---  856
KSR1-Saccoglossus-kowalevskii/1-898                 856 HPESRPTFGELMKALDRLRLPKK--------RLARSPSHPLH---LSRSAESV---  897
KSR1-Branchiostoma-floridae/1-865                   825 QPDKRPQFSDLTQTLD--RLPRK--------RLTRSPSTPLH---LSRSAESI---  864
KSR1-Capitella-teleta/1-536                         496 SVDERPNYSRLLKTFE--RLPRK--------RLARCPSHPIQ---LSRSAESV---  535
KSR1-Rhipicephalus-sanguineus/1-864                 824 KPQDRPAFAKLQVQLQ--RLPKK--------RLARSPSHPTH---LSRSAESA---  863
KSR1-Limulus-polyphemus/1-603                       563 KPVERPHFGNIQSTLL--KLPKK--------RLARSPSHPIH---LSRSAESV---  602
KSR3-Ramazzottius-variornatus-partial/1-451         403 LPEDRPTFAHILTSLKCKTFLHS----------HHSTSQPDL---LDVIGTSRSKS  445
KSR2-Homo-sapiens/1-950                             914 EQEERPTFTKLMDMLEKLPKRNR----------R--LSHPGH---FWKSAEL----  950
KSR2-Danio-rerio/1-966                              928 EQEERPSFSKLMELLEKLPKRNR----------R--LSHPVH---FWKSAEYVK--  966
KSR1-Homo-sapiens/1-923                             863 DLQERPSFSLLMDMLEKLPKLNR----------R--LSHPGH---FWKSADINSSK  903
KSR1-Danio-rerio/1-898                              862 DSRNRPAFTHLADMLEKLPKLNR----------R--LSHPGH---FWKSAEL----  898
KSR3-Clytia-hemispherica/1-517                      447 RIEKRPEFQEIINNMKKINIPRSLQK-------KLSLSDSGA---INKEQQKQ---  489
KSR3-Schmidtea-mediterranea/1-666                   624 DPLARPEFSDISRELMKRDTTRK----------RHSSSVPGN---LHQDNS-----  661
KSR3-Rhipicephalus-sanguineus/1-675                 629 APEARPSFVMIGRQLQENVTLHR----------KHSSSEPDR---LHHSGL-----  666
KSR3-Limulus-polyphemus/1-686                       640 SPSDRPGVVYIIKQLQESVSLHK----------RHSSSEPER---LHRMGT-----  677
KSR3-Limulus-polyphemus-isoformB/1-671              629 TPSDRPDVAYIIKQLQESFSLHK----------RHSSSEPER---LHRLG------  665
KSR3-Lingula-anatina/1-626                          585 LPSSRPGFVDINKILQTVVHLKK----------RHSSSEPDS---IHKIGV-----  622
KSR3-Crassostrea-gigas/1-585                        540 NQAKRPSFQDISKTLHKNANLHK----------QHSVSEPDI---SGRFGT-----  577
KSR3-Nematostella-vectensis/1-640                   585 NSEDRPDFAKIVQFFQEHNISHECSNK----RITGSHSEPEK---IHMVGR-----  628
KSR3-Acropora-millepora/1-634                       585 VQSERPGFSRIADFLQQQNVPPEVSRK----RIS--FSEPEK---MNFIGR-----  626
KSR3-Priapulus-caudatus/1-533                       489 M-QHRPNFKSILRKLQHGVPAGM--------HRRHSTSQPEG---LHRAGL-----  527
KSR3-Saccoglossus-kowaleskii/1-591                  545 HPLDRPLITEVVKELQHNVPLHK---------K-HSQSEPDQ---LNRL-------  580
KSR3-Ptychodera-flava/1-629                         578 EPLDRPKFTDIAKELQHNVSLHK---------K-HSQSNPEN---LNRIGR-----  615
KSR3-Trichoplax-adherens/1-510                      475 NIAQRPQFVDMAKSLQNNPIEGL---------ILAE-TIDYV---VNYC-------  510
KSR3-Capitella-teleta/1-642                         620 ---------------------HK----------RHSTSNPEK---LNKIGL-----  636
KSR3-Eucidaris-tribuloides/1-545                        --------------------------------------------------------
KSR3-Strongylocentrotus-purpuratus/1-602            560 NPNERPSFQDIVKELKKNVSLGN---------MKHSLSEPDN---LNRLGT-----  598
KSR3-Paracentrotus-lividus-RNAseq/1-600             558 EASERPLFPDIVKELKKNVSLGN---------MKHSLSEPDN---LNRLGT-----  596
KSR3-Notospermus-geniculatus/1-691                  646 SPKYRPDFAKINRELNENGALHK---------R-HSSSEPER---MNRVGL-----  683
KSR3-Aplysia-californica/1-405                      364 EPLYRPEFRELCKDLQRHSPLHK---------K-HSSSEPER---LNRATE-----  401
KSR3-Amphiura-filiformis-partial/1-642              636 DKTSRPT-------------------------------------------------  642
KSR3-Platynereis-dumerilii/1-704                    659 SPDDRPAFSNINKELQHNSALHK---------I-HSSSVPER---LNRSGI-----  696
KSR3-Patiria-miniata/1-779                          739 VPNVRPTFQEIVEELNQHAALVN---------MKHSLSEPDL---LNRIGR-----  777
KSR3-Anneissia-japonica/1-590                       547 DHLERPKFSSIMKDLQDNAALVN---------MNRSRSEPEN---LNRLGL-----  585
KSR3-Parastichopus-parvimensis-partial/1-330        287 KPEGRPSFSSILARLSQNVALVN---------RRHSLSEADL------KGR-----  322
```

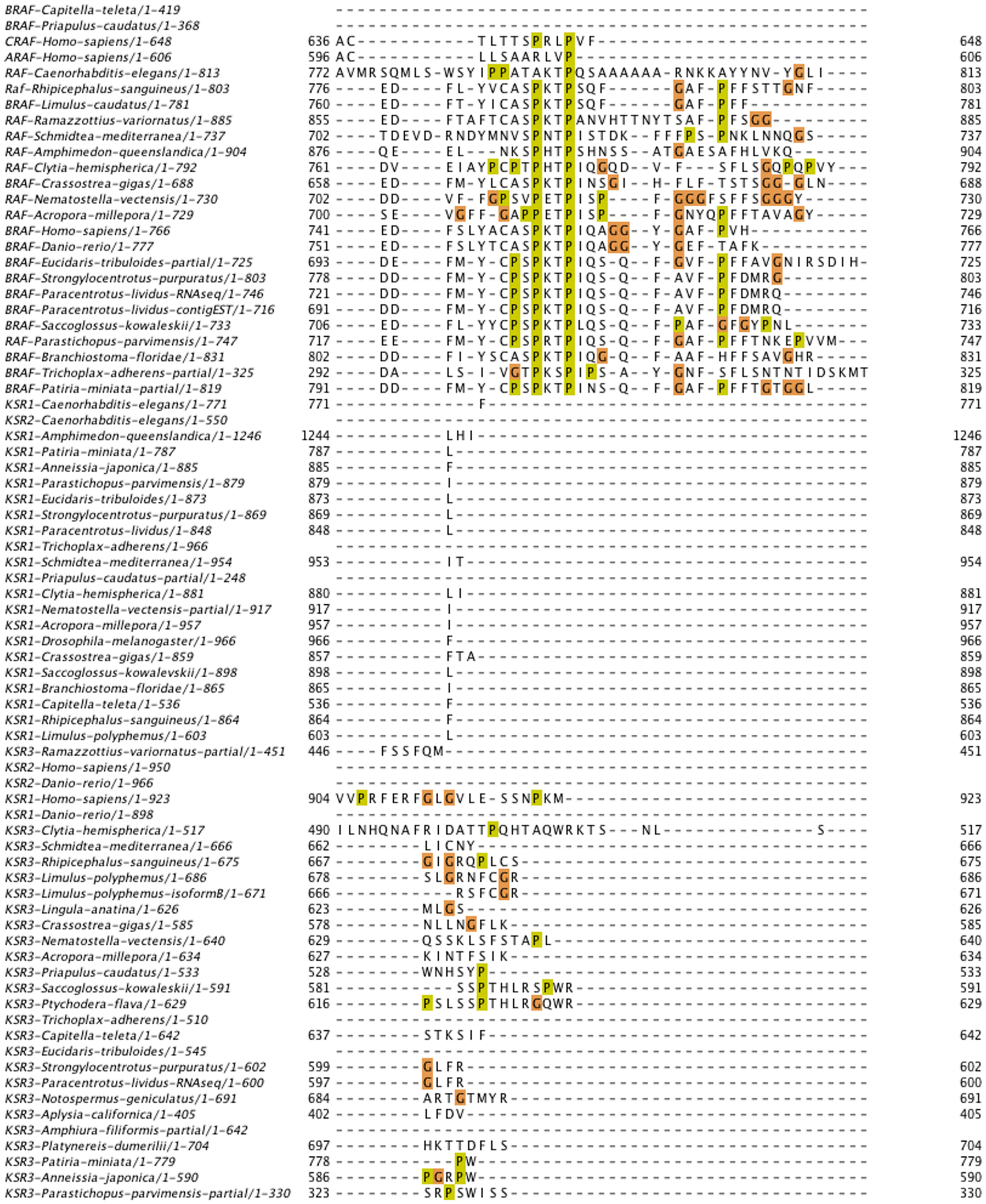
BRAF-Capitella-teleta/1-419
BRAF-Priapulus-caudatus/1-368
CRAF-Homo-sapiens/1-648 636 648
ARAF-Homo-sapiens/1-606 596 606
RAF-Caenorhabditis-elegans/1-813 772 813
Raf-Rhipicephalus-sanguineus/1-803 776 803
BRAF-Limulus-caudatus/1-781 760 781
RAF-Ramazzottius-variornatus/1-885 855 885
RAF-Schmidtea-mediterranea/1-737 702 737
RAF-Amphimedon-queenslandica/1-904 876 904
RAF-Clytia-hemispherica/1-792 761 792
BRAF-Crassostrea-gigas/1-688 658 688
RAF-Nematostella-vectensis/1-730 702 730
RAF-Acropora-millepora/1-729 700 729
BRAF-Homo-sapiens/1-766 741 766
BRAF-Danio-rerio/1-777 751 777
BRAF-Eucidaris-tribuloides-partial/1-725 693 725
BRAF-Strongylocentrotus-purpuratus/1-803 778 803
BRAF-Paracentrotus-lividus-RNAseq/1-746 721 746
BRAF-Paracentrotus-lividus-contigEST/1-716 691 716
BRAF-Saccoglossus-kowaleskii/1-733 706 733
RAF-Parastichopus-parvimensis/1-747 717 747
BRAF-Branchiostoma-floridae/1-831 802 831
BRAF-Trichoplax-adherens-partial/1-325 292 325
BRAF-Patiria-miniata-partial/1-819 791 819
KSR1-Caenorhabditis-elegans/1-771 771 771
KSR2-Caenorhabditis-elegans/1-550
KSR1-Amphimedon-queenslandica/1-1246 1244 1246
KSR1-Patiria-miniata/1-787 787 787
KSR1-Anneissia-japonica/1-885 885 885
KSR1-Parastichopus-parvimensis/1-879 879 879
KSR1-Eucidaris-tribuloides/1-873 873 873
KSR1-Strongylocentrotus-purpuratus/1-869 869 869
KSR1-Paracentrotus-lividus/1-848 848 848
KSR1-Trichoplax-adherens/1-966
KSR1-Schmidtea-mediterranea/1-954 953 954
KSR1-Priapulus-caudatus-partial/1-248
KSR1-Clytia-hemispherica/1-881 880 881
KSR1-Nematostella-vectensis-partial/1-917 917 917
KSR1-Acropora-millepora/1-957 957 957
KSR1-Drosophila-melanogaster/1-966 966 966
KSR1-Crassostrea-gigas/1-859 857 859
KSR1-Saccoglossus-kowalevskii/1-898 898 898
KSR1-Branchiostoma-floridae/1-865 865 865
KSR1-Capitella-teleta/1-536 536 536
KSR1-Rhipicephalus-sanguineus/1-864 864 864
KSR1-Limulus-polyphemus/1-603 603 603
KSR3-Ramazzottius-variornatus-partial/1-451 446 451
KSR2-Homo-sapiens/1-950
KSR2-Danio-rerio/1-966
KSR1-Homo-sapiens/1-923 904 923
KSR1-Danio-rerio/1-898
KSR3-Clytia-hemispherica/1-517 490 517
KSR3-Schmidtea-mediterranea/1-666 662 666
KSR3-Rhipicephalus-sanguineus/1-675 667 675
KSR3-Limulus-polyphemus/1-686 678 686
KSR3-Limulus-polyphemus-isoformB/1-671 666 671
KSR3-Lingula-anatina/1-626 623 626
KSR3-Crassostrea-gigas/1-585 578 585
KSR3-Nematostella-vectensis/1-640 629 640
KSR3-Acropora-millepora/1-634 627 634
KSR3-Priapulus-caudatus/1-533 528 533
KSR3-Saccoglossus-kowaleskii/1-591 581 591
KSR3-Ptychodera-flava/1-629 616 629
KSR3-Trichoplax-adherens/1-510
KSR3-Capitella-teleta/1-642 637 642
KSR3-Eucidaris-tribuloides/1-545
KSR3-Strongylocentrotus-purpuratus/1-602 599 602
KSR3-Paracentrotus-lividus-RNAseq/1-600 597 600
KSR3-Notospermus-geniculatus/1-691 684 691
KSR3-Aplysia-californica/1-405 402 405
KSR3-Amphiura-filiformis-partial/1-642
KSR3-Platynereis-dumerilii/1-704 697 704
KSR3-Patiria-miniata/1-779 778 779
KSR3-Anneissia-japonica/1-590 586 590
KSR3-Parastichopus-parvimensis-partial/1-330 323 330
